# STAT1 drives the immune landscape of murine Toll-like receptor 9-induced liver inflammation

**DOI:** 10.1016/j.jhepr.2025.101668

**Published:** 2025-11-06

**Authors:** Amber De Visscher, Jarne Beliën, Eline Bernaerts, Marte Vandeput, Bert Malengier-Devlies, Fran Prenen, Hanne Meers, Liliana Sokol, Tania Mitera, Nele Berghmans, Seray Anak, Olivier Govaere, Philippe Van den Steen, Jochen Lamote, Niels Vandamme, Anna Bujko, Charlotte L. Scott, Carine H. Wouters, Patrick Matthys

**Affiliations:** 1 Laboratory of Immunobiology, Department of Microbiology, Immunology, and Transplantation, Rega Institute for Medical Research, University of Leuven, Leuven, Belgium; 2 Laboratory for Neuroimmunology, Department of Neurosciences, Leuven Brain Institute, KU Leuven, Leuven, Belgium; 3 Centre for Reproductive Health, Institute for Regeneration and Repair, University of Edinburgh, Edinburgh, United Kingdom; 4 Laboratory of Immunoparasitology, Department of Microbiology, Immunology, and Transplantation, Rega Institute for Medical Research, KU Leuven, Leuven, Belgium; 5 Laboratory of Molecular Immunology, Department of Microbiology, Immunology, and Transplantation, Rega Institute for Medical Research, KU Leuven, Leuven, Belgium; 6 Translational Cell and Tissue Research unit, Department of Imaging & Pathology, KU Leuven, Leuven, Belgium; 7 VIB Flow Core Leuven, VIB Technologies, Leuven, Belgium; 8 VIB-KU Leuven Center for Cancer Biology, Department of Oncology, Biomedical Science Group, KU Leuven, Leuven, Belgium; 9 VIB Single Cell Core, VIB, Leuven-Ghent, Belgium; 10 Laboratory of Myeloid Cell Biology in Tissue Damage and Inflammation, VIB-UGent Centre for Inflammation Research, Technologiepark-Zwijnaarde, Ghent, Belgium; 11 Department of Biomedical Molecular Biology, Faculty of Sciences, Ghent University, Ghent, Belgium

**Keywords:** CpG-induced liver inflammation, IFN-α/β, IFN-γ, scRNA-seq, MAS, PSC

## Abstract

**Background & Aims:**

Persistent activation of Toll-like receptor 9 (TLR9) has been implicated in eliciting a cytokine storm syndrome, leading to systemic and hepatic inflammation in mice and humans. This study investigates the unexplored role of STAT1, a transcription factor in pathogen-driven immune responses, in mediating TLR9-induced liver inflammation.

**Methods:**

We compared clinical, histological, and laboratory characteristics (in total nine parameters) of TLR9-induced liver inflammation between wild-type (WT) mice and STAT1-deficient (*Stat1*^*-/-*^) mice (n = 3–31 mice/condition depending on the parameter measured) and explored their hepatic immune landscape using single-cell CITE-sequencing (total of 36,585 CD45^+^ liver cells from four to eight mice/condition). Findings were validated by flow cytometry, treatment with biologicals, *ex vivo* cell culture, and exploration of publicly available patient datasets.

**Results:**

*Stat1*^*-/-*^ mice are protected against TLR9-induced inflammation as they do not develop the typical features seen in WT counterparts (*p* <0.05–0.0001, depending on the parameter). This protection is associated with the absence of hepatic cycling CD38^+^CD8^+^ T cells, type 2 conventional dendritic cells, and monocytes transitioning into inflammatory macrophages. These cell populations exhibit elevated STAT1 expression and type I and II interferon (IFN) signatures, resembling immune profiles of patients with cytokine storm syndromes and liver inflammation. *Ex vivo*, type I and II IFNs induce the phenotype of cycling T cells and transitioning monocytes through STAT1 signaling. *In vivo*, simultaneous treatment with anti-type I and II IFN antibodies in CpG-injected WT mice provide protection against systemic and liver inflammation (*p* <0.05–0.001 for five mice/condition).

**Conclusions:**

Type I and II IFN-induced STAT1 activation drives TLR9-induced liver inflammation, and support further exploration of JAK1/2 inhibitors, which indirectly inhibit STAT1 activity, in patients with cytokine storm syndromes and other inflammatory liver disorders.

**Impact and implications:**

Our study reveals that interferon-induced STAT1 signaling is a central mediator of both systemic and hepatic inflammation during a TLR9-induced cytokine storm. Based on these findings, we support the therapeutic use of JAK1/2 inhibitors, such as ruxolitinib and baricitinib, which indirectly suppress STAT1 activity, in patients with cytokine storm syndromes and inflammatory liver disorders to alleviate both systemic and hepatic symptoms. Notably, our data also highlight the promise of direct STAT1 inhibition as a more and potentially refined approach for intervention.

## Highlights

• Type I and II IFN-STAT1 signaling drives TLR9-induced systemic/liver inflammation.
• Liver inflammation consists of cycling T cells, transitioning monocytes, and cDC2s.
• IFNs promote the phenotype of cycling T cells and transitioning monocytes.
• Upregulated IFN-STAT1 signaling in patients with systemic liver pathology.

## Graphical abstract

**Figure:**
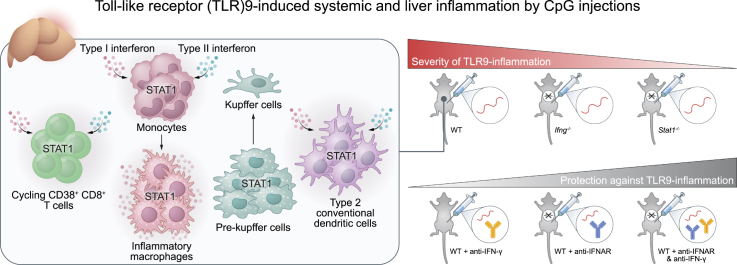

## Introduction

Over the past decade, interest in cytokine storm syndromes has intensified, particularly because of the emergence of a cytokine storm in a subset of patients with COVID-19.^1^ These syndromes arise from the failure to control and terminate an immune response, leading to T cell and macrophage (Mϕ) hyperactivation, which fuels a cytokine storm.^2^ Patients present with systemic inflammation, characterized by fever, pancytopenia, hepatosplenomegaly, coagulopathy, and liver dysfunction. Without adequate treatment, this progresses into multi-organ failure and death.^2^ Persistent activation of Toll-like receptors (TLRs), especially TLR9, has been implicated in initiating and perpetuating a cytokine storm.^3^^,^^4^ Correspondingly, a sterile, well-characterized murine model based on repetitive TLR9 triggering by cytosine-phosphate-guanine (CpG) injections (developed by Behrens *et al.*^5^) phenocopies this inflammation, including liver inflammation, without the interference of infectious agents. However, sterile TLR9-induced liver inflammation remains poorly characterized.

Within the TLR9-induced cytokine storm, type II interferon (IFN-γ) has been established as a key cytokine.^5^^,^^6^ Binding to its receptor activates Janus kinase (JAK)1 and JAK2, which subsequently activate Signal transducer and activator of transcription (STAT)1.^7^ Notably, levels of total and phosphorylated STAT1 (pSTAT1) are elevated in livers of patients with cytokine storm syndrome,^8^ implicating the JAK1/2–STAT1 axis in the disease pathogenesis. Clinical studies also demonstrated the efficacy of JAK1/2 inhibitors (*e.g.* ruxolitinib and baricitinib, which indirectly inhibit STAT1 activation) in patients with cytokine storm syndrome.[9], [10], [11] However, the precise mechanism driving remission remains incompletely understood.

In this context, Albeituni *et al.*^12^ showed that ruxolitinib confers superior protection compared to IFN-γ neutralization alone in a TLR9-induced murine model, suggesting the involvement of other mechanisms, for example IFN-γ-independent STAT1 signaling. Despite these insights,[9], [10], [11], [12] the specific contribution of STAT1 and the cytokines driving its activation in cytokine storm syndromes remains unclear.

Here, we demonstrate that both type I and II IFN-induced STAT1 activation drives murine TLR9-induced systemic, and in particular, liver inflammation. In-depth transcriptomic profiling identified a distinct inflammatory landscape composed of IFN-γ-producing cycling CD8^+^ effector T cells, type 2 conventional dendritic cells (cDC2s), and inflammatory monocytes differentiating into Mϕs.

## Materials and methods

### Mice and *in vivo* injections

Origin and housing of mice is detailed in the Supplementary Materials and methods. The model was induced in 8–10-week-old mice by intraperitoneal (i.p.) injections of CpG (50 μg, ODN1826, IDT) every 2 days as described.^5^ PBS-injected age/sex-matched mice served as controls. Our study examined male and female animals (similar findings are reported for both). The number of animals used depends on the measured parameter (n = 3–31 mice/condition). For blocking type I IFNs, mice were i.p.-injected with anti-interferon-α/β receptor (IFNAR) antibody (1,000 μg/ml MAR1-5A3, Leinco Technologies, St. Louis, MO, USA) kindly provided by Dr. Kai Dallmeier and Lara Kelchtermans (Laboratory of Virology & Antiviral Research, REGA institute, KU Leuven, Belgium) and for blocking type II IFNs, anti-IFN-γ antibody (F3, in-house made, 1,000 μg/ml) starting 1 day before CpG injection and repeated at days 3 and 7. Experiments were approved by the Ethics Committee of KU Leuven (P223/2017 and P104/2021).

### Isolation of liver non-parenchymal cells

Mice were euthanized by an i.p. injection of dolethal (0.2 mg, Vétoquinol, Niel, Belgium) and organs were dissected. Livers were fragmented in RPMI 1640 (Gibco, Avantor, Leuven, Belgium) with Collagenase D (2 mg/ml, Sigma, Hoeilaart, Belgium), DNase I (0.2 mg/ml, Sigma), and 2% heat-inactivated FBS using the GentleMACS dissociator (Miltenyi Biotec, Leiden, The Netherlands). Fragmented tissue was incubated for 30 min at 37 °C and filtered through a 70 μm cell strainer. Leukocytes were isolated via density centrifugation with 37.5% Percoll (GE Healthcare), as described.^13^ For *in vivo* isolation, mice were sedated/euthanized by an i.p. injection of dolethal (0.50 mg), and cells were isolated by *ex vivo* liver perfusion as described.^14^

### Blood analysis and ALT measurement

Blood samples were obtained by submandibular bleeding with heparin (500 units/ml, LEO pharma, Lier Belgium) for complete blood cell analysis (Advia 2120i hematology system, Siemens) or without heparin for alanine aminotransferase (ALT) measurement (ALT/GPT activity kit, ThermoFisher Scientific Diagnostics, Merelbeke, Belgium).

### Histology of liver sections

Liver tissue was fixed in 10% formalin (VWR), gradually dehydrated, and embedded in paraffin. Sections (5 μm) were stained with H&E Staining Kit (4501565630, Abcam, Cambridge, UK). Liver sections were blindly scored (explained in the Supplementary Materials and methods).

### RNA extraction and RT-qPCR

Liver tissue was mechanically homogenized in RLT buffer from the RNeasy Mini Kit (Qiagen, Venlo, The Netherlands) and RNA was extracted according to the manufacturer’s guidelines. For each sample, cDNA was synthesized from 1,000 ng RNA using the High-capacity cDNA reverse transcription Kit (Life Technologies, Merelbeke, Belgium). Quantitative reverse transcription-polymerase chain reaction (qRT-PCR) was performed in duplicate with primer and probe sets from Integrated DNA Technologies using an ABI Prism 7500 Sequence Detection System (Life Technologies). Data were normalized to the 18S ribosomal RNA levels. The fold change was compared to the average of PBS-injected wild-type (WT) mice and calculated using the 2^−ΔΔCT^ method. Primers are listed in Table S1.

### Protein extraction and Western blot

Protein extraction, SDS-PAGE, and protein transfer are described in the Supplementary Materials and methods. Membranes were blocked with 5% non-fat dry milk (BioRad) diluted in tris-buffered saline Tween (TBST) buffer for 1 h at room temperature, and incubated with rabbit anti-mouse STAT1 or pSTAT1 (Cell Signaling Technologies, Leiden, The Netherlands) or mouse anti-mouse horseradish peroxidase (HRP)-tubulin (Thermo Fisher Scientific)] overnight at 4 °C. After washing, membranes were incubated with donkey anti-rabbit HRP secondary antibody (Jackson ImmunoResearch, Cambridgeshire, United Kingdom). Membranes were exposed to a chemiluminescent substrate (SuperSignal west pico PLUS chemiluminescent substrate or SuperSignal west femto maximum sensitivity substrate, Thermo Fisher Scientific). Protein bands were captured by the Fusion Solo S (Vilber Lourmat). Between detection of pSTAT1 and STAT1, membranes were stripped with stripping buffer (0.1 M glycine, pH 2.8). Protein band intensities were measured with ImageJ. Values obtained for STAT and pSTAT were first normalized against the housekeeping protein tubulin. Outliers in the pSTAT/STAT ratio were identified by the ROUT method (Q = 1%) and excluded from the analysis.

### *Ex vivo* cell culture

Liver cells isolated from CpG-injected WT mice through *ex vivo* digestion were cultured (10^6^ cells/ml) for 24 h with IFN-α (1,000 U/ml, Pbl), IFN-β (100 ng/ml, R&D), IFN-γ (150 ng/ml, PeproTech, Life Technologies), and/or baricitinib-phosphate (20 μM, MedChemExpress) in RPMI 1640 medium (Gibco) containing 10% fetal bovine serum.

### Flow cytometry

Cells were incubated with FcR blocking reagent (1:100, Miltenyi Biotec), anti-FcγRIV antibody^15^ (1:100, 9E9, BioLegend), and ZombieAqua516 (1:1,000, BioLegend). Cells were stained at 4 °C during 25 min in FACS buffer (PBS with 2% FBS and 2 mM EDTA) with 25% brilliant stain buffer (BD Biosciences, Erembodegem, Belgium), and 10% rat serum (ThermoFisher Scientific). For cytokine detection, samples were incubated with GolgiStop™ and GolgiPlug™ protein transport inhibitors (1:1,000, BD Biosciences) for 1 h at 37 °C, and staining was performed with the Cytofix/Cytoperm™ Fixation/Permeabilisation kit (BD Biosciences). For staining of STAT1, cells were fixed with 1X BD Phosflow™ Lyse/Fix solution (BD Biosciences) for 10 min at 37 °C and permeabilized with BD Phosflow™ Perm III. The antibodies are listed in Table S2. Analysis was performed on a BD LSR Fortessa X20 with DIVA software. Data were analyzed using FlowJo (version 10.9). The flowAI plugin was used to perform an automatic quality control on the flow cytometry data which detects low-quality events by evaluating the flow rate, signal acquisition, and dynamic range, and these were removed. A representative gating strategy is depicted in Figs S6B, S7A, and S8A and C.

### Single-cell transcriptomics

For FACS and CITE-seq antibody labelling, 1–2 × 10^6^ liver cells of a pool from four to eight mice (1:1 male:female) were stained with fluorochrome-labelled antibodies (Table S2), Tru-Stain FcX Block (BioLegend, Amsterdam, The Netherlands), and oligo-conjugated antibodies (195 TotalSeq-C antibodies and 4 TotalSeq-C isotype controls, Table S3). Cell sorting was performed with BD FACS Aria Fusion. Sorted populations (69,000 CD45^+^ living cells enriched with 1,000 CD3^-^CD19^-^CD45^+^F4/80^+^ living cells) were loaded onto a Chromium GemCode Single Cell Instrument for the formation of single-cell suspensions, followed by preparation of scRNA-seq libraries according to manufacturer’s guidelines (Chromium Next GEM Single Cell 5′ Kit version 2, 1000244, and library construction kit, 1000190, 10 × Genomics). Sequencing was performed with Illumina NovaSeq flow cell at the VIB Nucleomics core. Libraries were pooled at an 85:15 ratio for 5′ gene expression and cell surface protein sequencing. The R pipeline used for subsequent analysis of the CITE-seq data and exploration of publicly available human datasets is detailed in the Supplementary Materials and methods.

### Statistical analysis

GraphPad Prism (version 10.1.0, GraphPad Software, San Diego, CA, USA) was used for data analysis and graphing. Outliers were identified by the ROUT method (Q = 1%) and excluded from analysis. Before statistical testing, normality was checked by the Shapiro–Wilk test. To compare two unpaired groups, the parametric Student’s *t* test or non-parametric Mann–Whitney *U* test was used in case the data were normally or not normally distributed, respectively. Comparison of three or more unpaired groups was performed using one-way ANOVA followed by the Šídák's multiple-comparisons test or the non-parametric Kruskal–Wallis test followed by the Dunn’s multiple-comparisons test if the data were normally or not normally distributed, respectively. For the comparison of three or more paired groups, one-way ANOVA with Geisser–Greenhouse correction followed by a Šídák's multiple-comparisons test or a Friedman test followed by Dunn’s multiple-comparison test was performed if the data were normally or not normally distributed, respectively. Regarding transcriptomics, statistical tests were performed in R (version 4.2.2, R Foundation for Statistical Computing, Vienna, Austria) and is detailed in the Supplementary Materials and methods.

## Results

### STAT1 deficiency provides enhanced protection against clinical features of TLR9-induced liver inflammation compared to IFN-γ deficiency

Given the established role of abnormal TLR signaling in cytokine storm syndromes,^3^^,^^4^ we utilized a well-characterized TLR9 model in which mice receive five i.p. injections with CpG oligonucleotides over 10 days. This induces systemic inflammation, manifesting as pancytopenia, hepatosplenomegaly, and liver inflammation, without overt liver injury^5^ (normal ALT levels, Fig. S1).

**Fig. 1:**
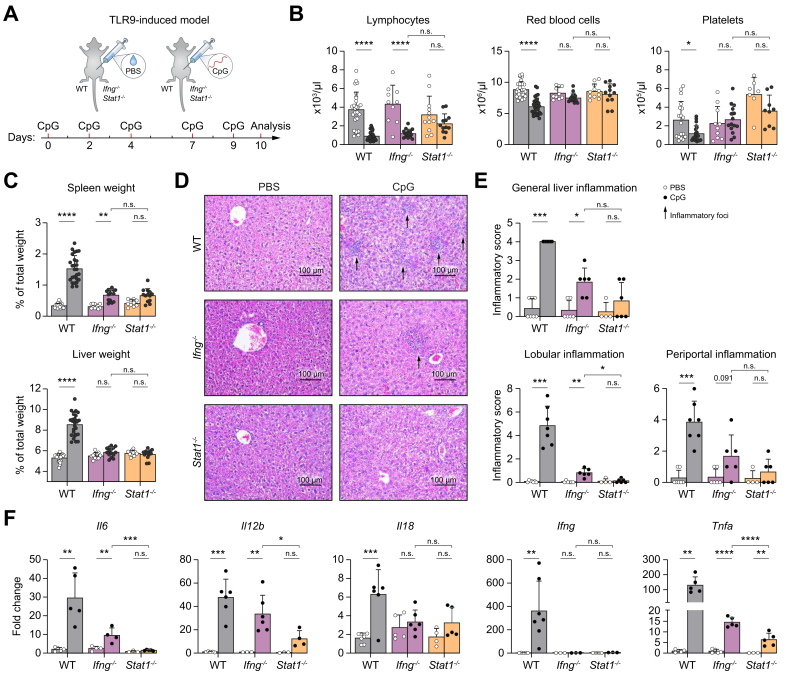
STAT1 deficiency provides enhanced protection against TLR9-induced clinical features compared with IFN-γ deficiency. (A) Experimental setup. (B) Blood counts. (C) Organ/body weight. (D) Representative liver sections with H&E staining and (E) quantification of liver inflammation. (F) Cytokine expression by qRT-PCR in livers. Bars (mean) and error bars (standard deviation). ns *p*>0.05, ∗*p* <0.05, ∗∗*p* <0.01, ∗∗∗*p* <0.001, ∗∗∗∗*p* <0.0001 (Student’s *t* test [WT in C, F – *Il6/Il12b/Tnfa/Il18*], Mann–Whitney *U* test [WT in B, E, F – *Ifng*], Šídák's multiple-comparisons test [*Ifng*^*-/-*^, *Stat1*^*-/-*^ in B, C – liver, F – *Il6*/*Il12*/*Tnfa*], Dunn’s multiple-comparisons test [*Ifng*^*-/-*^, *Stat1*^*-/-*^ in C – spleen, E, F – *Il18*, *Ifng*]). CpG, cytosine-phosphate-guanine; IFN, interferon; qRT-PCR, quantitative reverse transcription-polymerase chain reaction; STAT1, signal transducer and activator of transcription 1; TLR, Toll-like receptor; WT, wild type.

To determine the contribution of STAT1 signaling induced by cytokines other than IFN-γ, we compared TLR9-induced clinical features of *Ifng*^*-/-*^ mice with S*tat1*^*-/-*^ mice (Fig. 1A). While *Ifng*^*-/-*^ mice show protection against the development of anemia, thrombocytopenia, and hepatomegaly, they still present with lymphopenia and splenomegaly. In contrast, *Stat1*^*-/-*^ mice do not develop any of these pathological features (Fig. 1B and C).

STAT1 deficiency provides enhanced protection against liver pathology compared with IFN-γ deficiency, specifically regarding the lobular inflammation (Fig, 1D and E). Correspondingly, the inflammatory cytokines *Il6*, *Il12b*, and *Tnfa*, but not *Il18*, are significantly lower in livers of *Stat1*^*-/-*^ mice compared with *Ifng*^*-/-*^ mice. Notably, *Stat1*^*-/-*^ mice show no expression of *Ifng* upon TLR9 triggering (Fig. 1F).

In conclusion, although IFN-γ deficiency provides partial protection against TLR9-induced inflammation, STAT1 deficiency yields a more complete protection, particularly against liver inflammation.

### Upregulation of STAT1 and changes in hepatic T cell, dendritic cell, and monocyte/macrophage populations during TLR9-induced liver inflammation

**Fig. 2:**
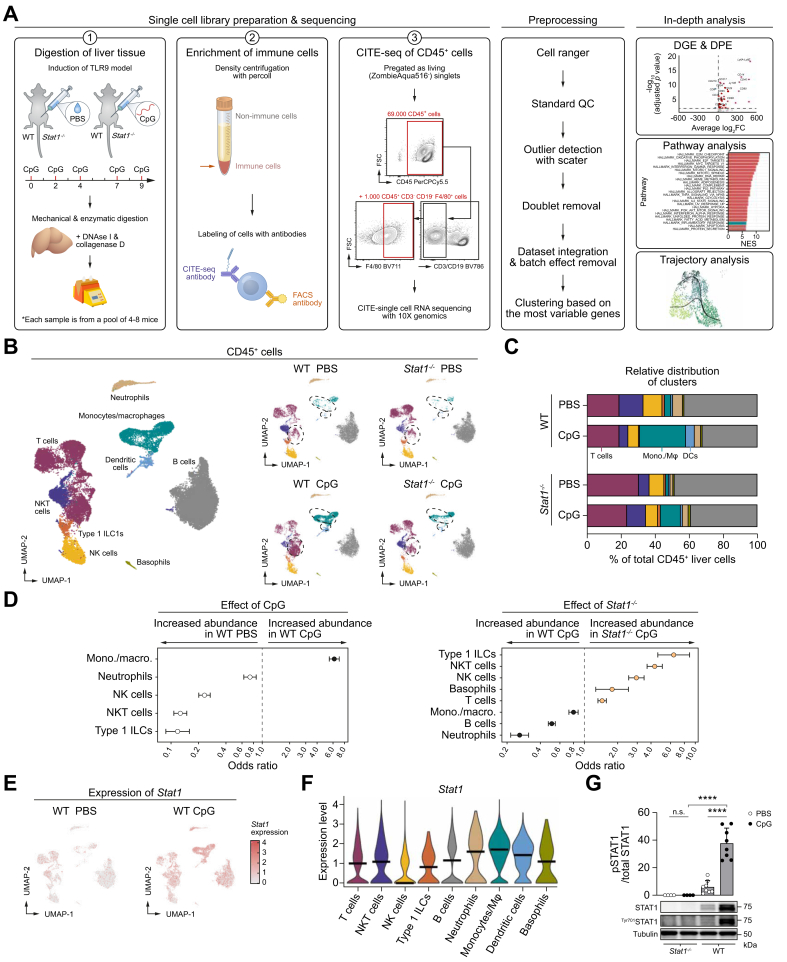
Upregulation of STAT1 and changes in T cells, DCs, and monocytes/macrophages in TLR9-mediated liver inflammation. (A) Experimental setup. (B) Integrated/separate datasets from scRNA-seq. Clusters discussed in the results are circled. (C) Cluster/total CD45^+^ liver cells. Colors correspond with clusters in panel B. (D) Odds ratio calculated by MASC with condition (WT PBS *vs.* WT CpG) or genotype (WT CpG *vs. Stat1*^*-/-*^ CpG) as contrast of interest (*p* <0.05). (E) *Stat1* expression. (F) *Stat1* expression in clusters of WT CpG. Colors correspond with clusters in panel B. Horizontal lines (median). (G) Ratio phosphorylated-STAT1 (^Tyr701^STAT1) to total STAT1 in liver lysate (calculations as described in methods) with a representative Western blot image. All uncropped Western blots are shown in Fig. S2D and S13. ns, *p* >0.05, ∗∗∗∗*p* <0.0001 (Šídák's multiple-comparisons test [in G]). CpG, cytosine-phosphate-guanine; STAT1, signal transducer and activator of transcription 1; TLR, Toll-like receptor; WT, wild type.

To explore the immune landscape of TLR9-induced liver inflammation and the contribution of STAT1 signaling, we performed CITE-seq on the liver from PBS-injected WT (WT PBS) and *Stat1*^*-/-*^ mice (*Stat1*^*-/-*^ PBS) and CpG-injected WT (WT CpG) and *Stat1*^*-/-*^ mice (*Stat1*^*-/-*^ CpG). Of each condition, a pool of cells (four to eight mice) was stained with FACS and CITE-seq antibodies. Sorted CD45^+^ liver cells were slightly enriched with F4/80^+^ cells (as described in the Materials and methods) to ensure a good resolution of the monocyte/Mϕ compartment (Fig. 2A). A total of 9,904, 8,708, 8,069, and 9,904 cells of WT PBS, WT CpG, *Stat1*^*-/-*^ PBS, and *Stat1*^*-/-*^ CpG, respectively, passed quality control and were included in the analysis (Fig. 2B). Validation of the annotation of the subclusters are described in Fig. S2A and B.

CpG triggering in WT mice results in a dramatic increase in the monocyte/Mϕ cluster, the dendritic cell (DC) cluster, and a subset of cells within the T cell cluster. In contrast, *Stat1*^*-/-*^ mice are protected to some extent from those changes, maintaining an immune landscape that closely resembles that of naive mice (WT and *Stat1*^*-/-*^ PBS) (Fig. 2B and C). Differential composition analysis demonstrated that the increased abundance of the monocyte/Mϕ cluster in WT CpG is significant compared to WT PBS, as well as the decreased abundance of this cluster in *Stat1*^*-/-*^ CpG compared with WT CpG (Fig. 2D).

Furthermore, differential gene expression (DGE) analysis showed that *Stat1* is significantly upregulated in all immune populations of WT CpG compared with WT *PBS* (Fig. 2E and Fig. S2C), with the highest expression in CpG-induced myeloid cells compared with other CpG-induced populations (Fig. 2E and F). Using Western blot on snap-frozen liver tissue, we confirmed the phosphorylation of STAT1 (pSTAT1) during TLR9 triggering in WT mice (Fig. 2G, and Figs S2D and S13). On average, a 6.3-fold increase in the pSTAT1/STAT1 ratio was found in CpG WT compared to PBS WT mice (average ratios for eight mice/group ± SE were 37.7 ± 11.1 and 6.0 ± 4.7, respectively).

Taken together, this identifies STAT1 as a potential driver of the expansion of leukocyte populations during CpG-induced inflammation.

### STAT1 is associated with expansion of hepatic cycling T cells in TLR9-induced liver inflammation

**Fig. 3:**
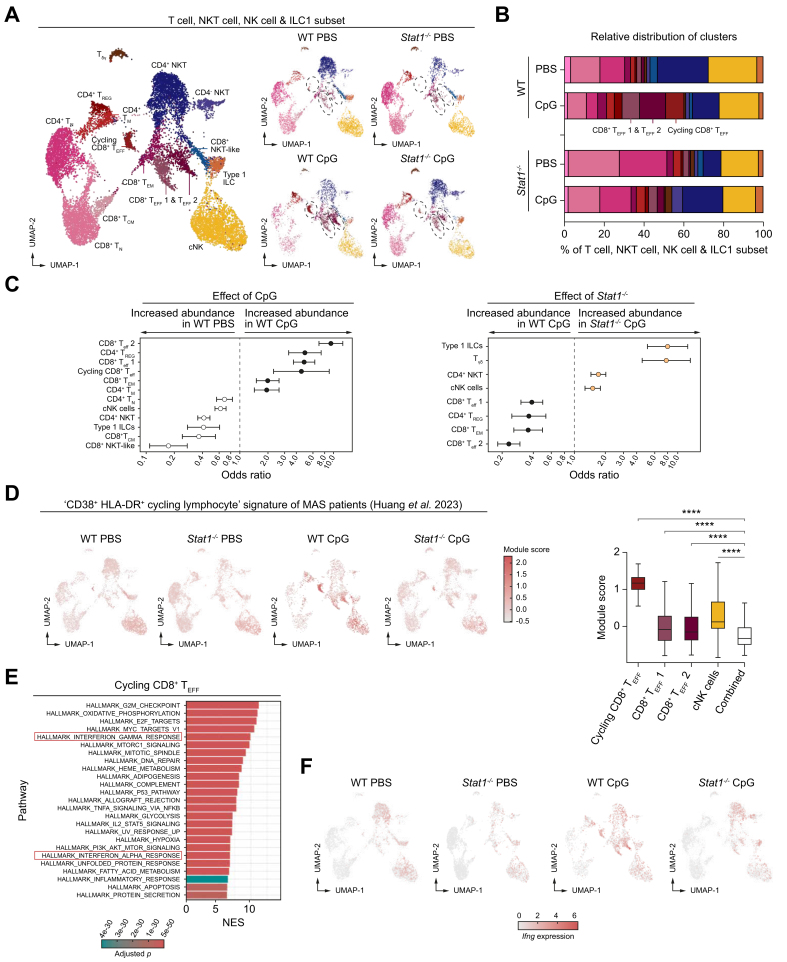
STAT1 is associated with the expansion of cycling T cells in TLR9-mediated liver inflammation. Experimental setup is shown in Fig. 2A. (A) Lymphoid subclustering of integrated/separate datasets. Clusters discussed in the results are circled. (B) Cluster/total lymphocytes. Colors of clusters shown in panel A. (C) Odds ratios calculated by MASC with treatment (WT PBS *vs.* WT CpG) or genotype (WT CpG *vs. Stat1*^*-/-*^ CpG) as contrast of interest (*p* <0.05). (D) Module score ‘CD38^+^HLA-DR^+^ cycling lymphocyte-signature’ patients with MAS (Huang *et al.*^16^). Whiskers (1.5× interquartile range). Combined (remaining clusters not shown individually). (E) GSEA from WT CpG (*p*_*corrected*_ <0.05). (F) *Ifng* expression. CpG, cytosine-phosphate-guanine; GSEA, gene set enrichment analysis; ILC, innate lymphoid cells; MAS, macrophage activation syndrome; NKT, natural killer T cells, STAT1, signal transducer and activator of transcription 1; T_CM_, central memory T cells, T_EFF_, effector T cells; T_EM_, effector memory T cells; T_N_, naive T cells; TLR, Toll-like receptor; T_REG_, regulatory T cells; WT, wild type.

Subclustering of the lymphocytes revealed that the CpG-induced T cell compartment consists mainly of a cycling CD8^+^ effector T (T_eff_) cell population and two non-cycling CD8^+^ T_eff_ cell populations (Fig. 3A and B). Differential composition analysis confirmed the increased presence of all three CD8^+^ T_eff_ cell populations in WT CpG compared to WT PBS. Validation of the annotation of the subclusters is shown in Fig. S3A and B.

A recent single-cell transcriptomics study from Huang *et al.*^16^ identified the presence of peripheral CD38^+^ HLA-DR^+^ cycling lymphocytes, comprised of CD4^+^ T cells, CD8^+^ T cells, and cNK cells, in patients with macrophage activation syndrome (MAS), a cytokine storm syndrome which develops in the context of rheumatic disorders. Evaluation of this ‘cycling lymphocyte’ signature in our murine model demonstrated a clear enrichment in CpG-induced cycling CD8^+^ T_eff_ cells and, to some extent, in two non-cycling CD8^+^ T_eff_ cell clusters and cNK cell clusters (Fig. 3D). DGE analysis showed that the CD8^+^ T_eff_ cell population 1 displays a quiescent cell state (*Klf3*), while the CD8^+^ T_eff_ cell population 2 displays an inflammatory (*Ccl3* and *Ccl4*) and exhausted phenotype (*Rgs1*, *Rgs16*, *Nr4a1*, and *Nr4a2*) (Fig. S3C). To explore the signaling pathways driving the development of cycling CD8^+^ T_eff_ cells, gene set enrichment analysis (GSEA) was performed. In addition to proliferation pathways, we found a type I IFN (IFN-α) and type II IFN (IFN-γ) signature (Fig. 3E), two cytokines that activate STAT1.^17^ Expression of type I IFNs was not detectable in the CITE-seq data, however, type II IFNs (*Ifng*) are clearly produced by multiple lymphocyte subsets in WT CpG, particularly by CD8^+^ T_eff_ cells (Fig. 3F). In both cycling and non-cycling CD8^+^ T cells, we found enriched expression of *Il10*, a cytokine that plays a protective role in TLR9-induced inflammation^5^ (Fig. S3D).

In conclusion, TLR9-triggering results in the expansion of type I and II IFN-induced cycling CD8^+^ T_eff_ cells, reminiscent of cycling CD38^+^HLA-DR^+^ CD8^+^ T_eff_ cells found in blood of patients with cytokine storm syndrome (MAS).

### STAT1 is associated with the expansion of hepatic transitioning monocytes and type 2 cDCs in TLR9-induced liver inflammation

**Fig. 4:**
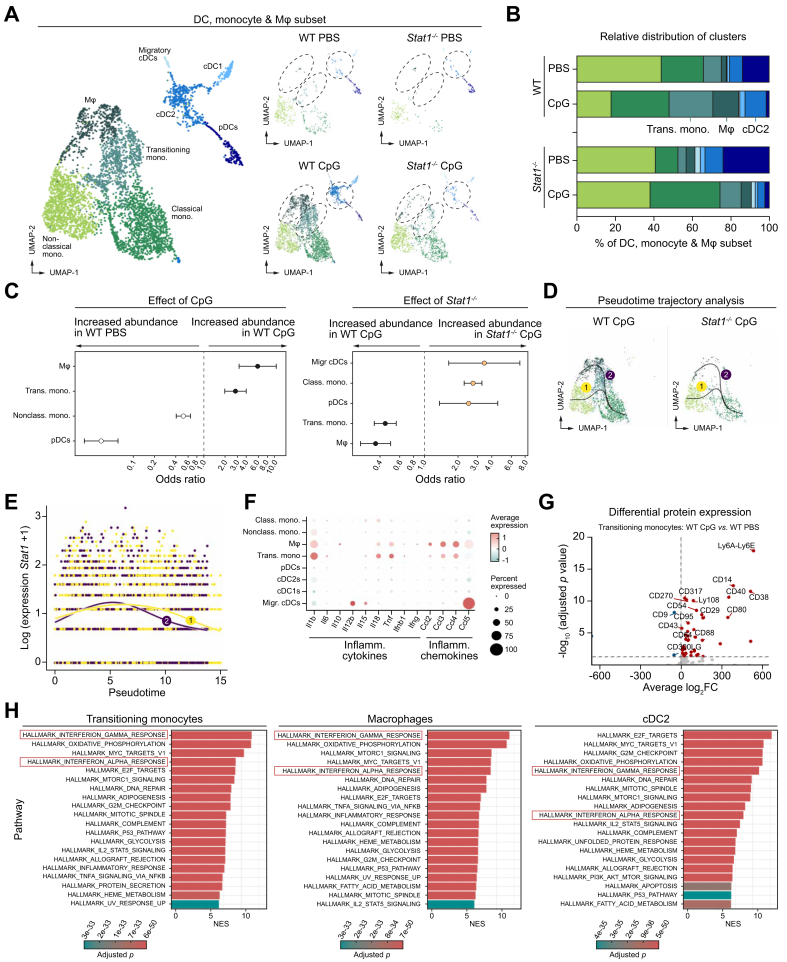
STAT1 is associated with expansion of transitioning monocytes and cDC2s in TLR9-challenged livers. Experimental setup is shown in Fig. 2A. (A) Myeloid subclustering of integrated/separate datasets. Clusters discussed in the results are circled. (B) Cluster/total myeloid cells. Colors of clusters shown in panel A. (C) Odds ratios calculated by MASC with condition (WT PBS *vs.* WT CpG) or genotype (WT CpG *vs. Stat1*^*-/-*^ CpG) as contrast of interest (*p* <0.05). (D) Pseudotime trajectory analysis with (E) *Stat1* expression. (F) Cytokine/chemokine expression in WT CpG. (G) Differential protein expression from transitioning monocytes (WT PBS *vs.* WT CpG). (H) GSEA from WT CpG (*p*_*corrected*_ <0.05). CpG, cytosine-phosphate-guanine; cDC; class, classical; conventional dendritic cell; pDC, plasmacytoid dendritic cells, GSEA, gene set enrichment analysis; Mϕ, macrophage; migr, migratory; STAT1, signal transducer and activator of transcription 1; TLR, Toll-like receptor; Trans; transitioning; WT, wild type.

Subclustering of the myeloid compartment revealed the presence of Mϕs, conventional type 2 DCs (cDC2), and transitioning monocytes upon TLR9 triggering (Fig. 4A and B). Differential composition analysis confirmed the enrichment of the monocyte and Mϕ populations in WT CpG compared to WT PBS, but could not confirm this for the cDC2s. Similarly, the monocyte and Mϕ populations are significantly reduced in *Stat1*^*-/-*^ CpG compared with WT CpG, but this could not be supported for cDC2s (Fig. 4C). Validation of the annotation of the subclusters is depicted in Fig. S4A and B. Of note, we could not identify a Kupffer cell (KC) population. The latter can be attributed to the use of collagenase D, a rather mild collagenase, in our *ex vivo* cell isolation protocol, whereas isolation of KCs requires the harsher collagenase A.^18^

As the transitioning monocyte cluster is suggestive of an intermediate cell state between monocytes and Mϕs, we performed a pseudotime trajectory analysis. Two distinct lineages are identified with the classical monocyte cluster set as starting point. Lineage 2 shows the differentiation of classical monocytes into Mϕs in WT CpG, while this differentiation is blocked in *Stat1*^*-/-*^ CpG (Fig. 4D). Expression of *Stat1* is also significantly associated with lineage 2 (Wald test = 51.02, *p* = 8.20 × 10^-05^) (Fig. 4E), suggesting that STAT1 drives the monocyte-to-Mϕ differentiation.

As TLR9 triggering is associated with a cytokine storm, we evaluated the expression of inflammatory cytokines and chemokines. Transitioning monocytes and Mϕs from WT CpG display increased expression of several inflammatory cytokines (*Il1b*, *Il18*, and *Tnf*) and chemokines (*Ccl2*, *Ccl3*, and *Ccl4*) (Fig. 4F). Differential protein expression (DPE) analysis of this cluster between WT CpG and WT PBS further revealed the upregulation of CD317, indicative of a type I or II IFN response,^19^ and Ly6A-Ly6E (SCA-1), a well-established marker of murine hematopoietic stem cells,^20^ suggestive of extramedullary monocytopoiesis. Additionally, proteins indicative of an activated phenotype (CD49a, CD54, and CD80) are upregulated (Fig. 4G). Moreover, GSEA of the three TLR9-associated myeloid populations demonstrated a similar type I and II IFN signature as observed in the CD8^+^ T_eff_ cell populations (Fig. 4H), suggesting that both cytokines drive STAT1 activation during monocyte-to-Mϕ differentiation.

Overall, our analysis suggests that type I and II IFN-induced activation of STAT1 is driving the expansion of inflammatory transitioning monocytes during TLR9-induced liver inflammation.

### STAT1 deficiency provides enhanced protection against the expansion of hepatic immune cell populations during TLR9-mediated liver inflammation in comparison to IFN-γ deficiency

The CITE-seq data predicted a type I and II IFN signature in multiple hepatic leukocyte populations associated with TLR9-induced liver inflammation. At first, we confirmed that both type I (*Irf7*, *Siglec1*, *and Mx1*) and II (*Cxcl9*, *Cxcl10*, and *Irf1*) IFN-stimulated genes (ISGs) are expressed during TLR9 triggering in total liver lysate using reverse transcription PCR (Fig. S5). Moreover, type I ISGs are still induced in CpG-injected *Ifng*^*-/-*^ mice, along with a mild but significant induction of *Cxcl9*. The latter is often considered a classical type II ISG, however, it has been demonstrated that type I IFNs can induce *Cxcl9* expression in the absence of *Ifng*.^21^ Expression of both type I and II ISGs is completely absent in CpG-injected *Stat1*^*-/-*^ mice.

We also validated the CITE-seq-identified hepatic populations associated with TLR9 triggering using flow cytometry in WT, *Ifng*^*-/-*^, and *Stat1*^*-/-*^ mice. Because the *ex vivo* isolation protocol fails to isolate KCs, we used an alternative isolation protocol involving *ex vivo* liver perfusion with collagenase A^18^ for the validation by flow cytometry (Fig. S6A). Based on the results of the CITE-seq data and literature,^18^ cycling CD8^+^ T cells were identified as CD45^+^CD19^-^NK1.1^-^F4/80^-^CD3^+^CD8^+^Ki67^+^CD38^+^ cells (Figs S6B and S7A and B), transitioning monocytes as CD45^+^CD3^-^CD19^-^NK1.1^-^ Ly6C^+^F4/80^+^ (Figs S6B and S8A,B), and cDC2s as CD45^+^CD3^-^CD19^-^NK1.1^-^Ly6C^-^F4/80^-^CD11c^+^CD172a^+^XCR1^-^ (Figs S6B and S8C and D).

**Fig. 5:**
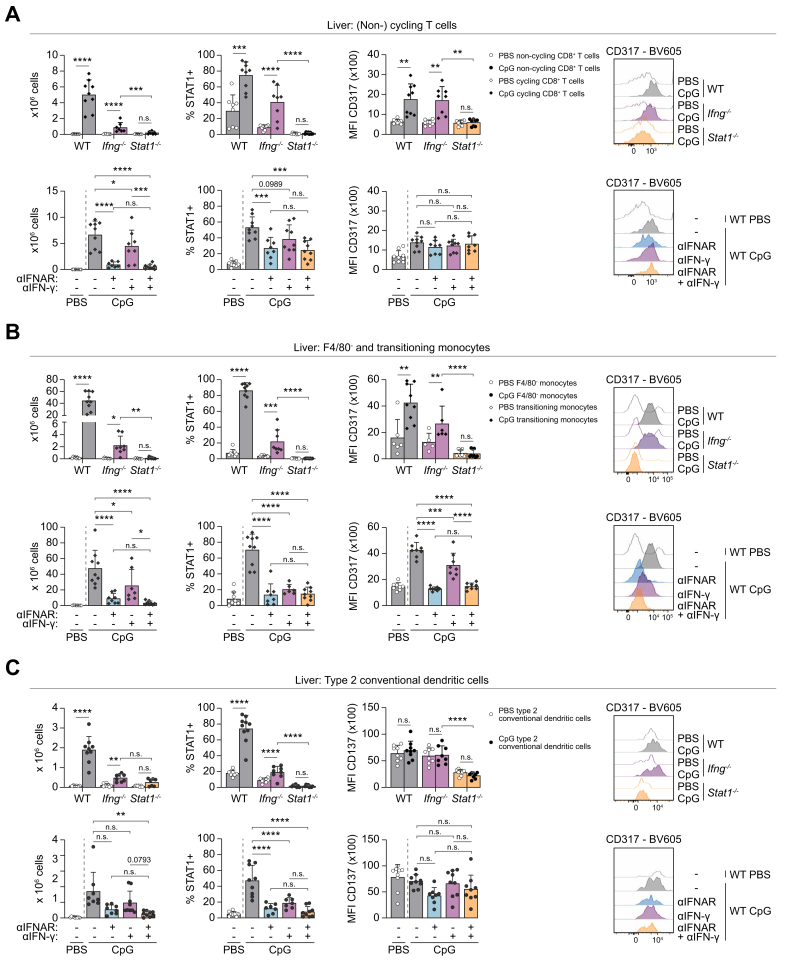
Type I and II interferons drive development of hepatic TLR9-associated leukocyte populations. Experimental setup and gating strategy are shown in Fig. S6. Absolute number of cells, %STAT1^+^ and MFI CD317 expression with representative histograms of (A) (non-) cycling T cells, (B) F4/80^-^ transitioning monocytes, and (C) cDC2. Bars (mean) and error bars (standard deviation). ns *p* >0.05, ∗*p* <0.05, ∗∗*p* <0.01, ∗∗∗*p* <0.001, ∗∗∗∗*p* <0.0001 (Student’s *t* test [WT in A, B – counts, C], Mann–Whitney *U* test (WT in B – STAT1/CD317), Šídák's multiple-comparisons test [*Ifng*^*-/-*^, *Stat1*^*-/-*^ in A – counts/STAT1, B – STAT1, C – STAT1/CD317; treatment in A, B, C – counts/STAT1], Dunn’s multiple-comparisons test [*Ifng*^*-/-*^, *Stat1*^*-/-*^ in A – CD317, B – counts/CD317, C – counts; treatment in C – counts]). CpG, cytosine-phosphate-guanine; DC, dendritic cell; STAT1, signal transducer and activator of transcription 1; TLR, Toll-like receptor; WT, wild type.

We confirmed the expansion of all three CpG-induced populations in WT mice and their absence in *Stat1*^*-/-*^ mice, while there is a limited but still significant increase in *Ifng*^*-/-*^ mice. Likewise, expression of STAT1 is highly induced in these cells from both WT and *Ifng*^*-/-*^ mice, but less potently in *Ifng*^*-/-*^ mice compared with WT mice. This is associated with an increased expression of CD317,^19^^,^^22^ confirming that CpG-induced cells underwent IFN stimulation in both WT and *Ifng*^*-/-*^ mice, while all IFN signaling is blocked in *Stat1*^*-/-*^ mice. Cycling T cells and transitioning monocytes are not found in PBS-injected mice or CpG-injected *Stat1*^*-/-*^ mice. Therefore, comparisons were made with the F4/80^-^ monocytes and the non-cycling T cells for transitioning monocytes and cycling T cells, respectively. Also, for cDC2s, CD317 expression could not validate the effect of IFN as it is expressed on DCs during homeostasis^19^^,^^22^ (Fig. 5A–C and Figs S7A, S8A and C, and S9A–C). Moreover, hepatic cycling T cells and cNK cells are sources of type II IFNs (IFN-γ) (Fig. S9), as observed in the transcriptome data (Fig. 3F).

**Fig. 6:**
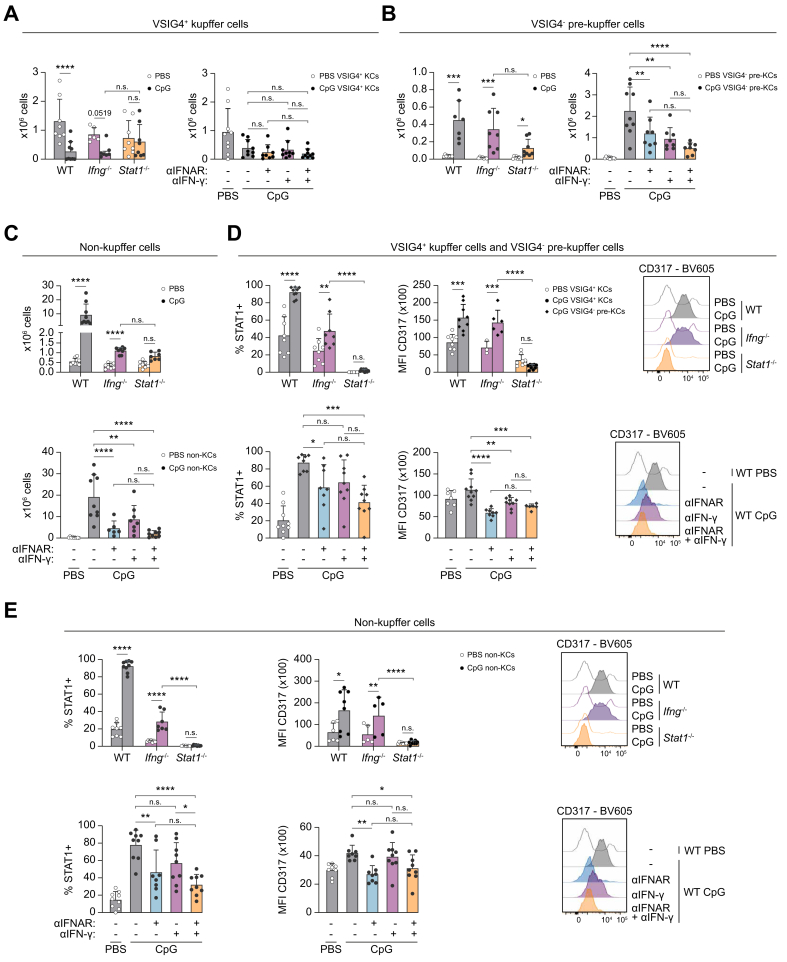
Loss of Kupffer cells and expansion of non-Kupffer cells in TLR9-induced liver inflammation. Experimental setup and gating strategy are shown in Fig. S6A and B. Absolute numbers of (A) VSIG4^+^ KCs, (B) VSIG4^-^ pre-KCs, and (C) non-KCs. (D&E) %STAT1^+^ cells and MFI CD317 expression with representative histograms of (D) VSIG4^+^ KCs and VSIG4^-^ pre-KCs, and (E) non-KCs. Bars (mean) and error bars (standard deviation). ns *p* >0.05, ∗*p* <0.05, ∗∗*p* <0.01, ∗∗∗*p* <0.001, ∗∗∗∗*p* <0.0001 (Student’s *t* test [WT in B, D, E], Mann–Whitney *U* test [WT in A, C], Šídák's multiple-comparisons test [*Ifng*^*-/-*^, *Stat1*^*-/-*^ in A, C–E; treatment in B–E], Dunn’s multiple-comparisons test [*Ifng*^*-/-*^, *Stat1*^*-/-*^ in A-B; treatment in A, C]). CpG, cytosine-phosphate-guanine; KC, Kupffer cell; STAT1, signal transducer and activator of transcription 1; TLR, Toll-like receptor; WT, wild type.

Because KCs could not be studied in the CITE-seq data, we investigated KCs (CD45^+^CD3^-^CD19^-^NK1.1^-^Ly6C^-^F4/80^+^VSIG4^+^CLEC2^+^) in this flow cytometry dataset (Fig. S6A and B). Surprisingly, VSIG4^+^ KCs are severely depleted upon CpG triggering (Fig. 6A), while a population reminiscent of pre-KCs (CD45^+^CD3^-^CD19^-^NK1.1^-^Ly6C^-^F4/80^+^VSIG4^-^CLEC2^+^) is induced (Fig. 6B). Pre-KCs exhibit elevated expression of STAT1 and CD317 in both WT and *Ifng*^*-/-*^ mice, while *Stat1*^*-/-*^ mice are protected from these changes (Fig. 6D and Fig. S11A). Non-KC Mϕs (CD45^+^CD3^-^CD19^-^NK1.1^-^Ly6C^-^F4/80^+^VSIG4^-^CLEC2^-^) expressing STAT1 and CD317, corresponding to the identified Mϕs in the CITE-seq data, are still present in *Ifng*^*-/-*^ mice, but completely absent in *Stat1*^*-/-*^ mice (Fig. 6C and E and Fig. S11B).

**Fig. 7:**
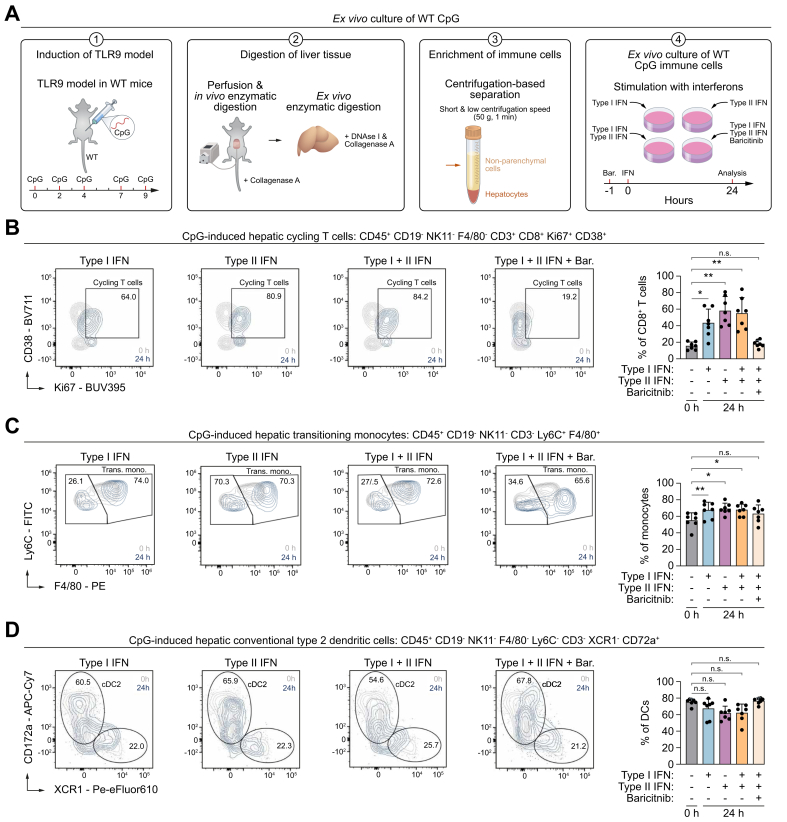
IFN-induced STAT1 signaling promotes the phenotype of cycling T cells and transitioning monocytes *ex vivo*. (A) Experimental setup: isolation of liver cells from CpG-injected WT mice followed by *ex vivo* stimulation with IFNs and/or baricitinib. (B–D) Representative flow plots and percentages of indicated populations. Grey plots (CpG-induced cells, no *ex vivo* stimulation) and blue plots (CpG-induced cells, 24 h *ex vivo* stimulation with IFNs and/or baricitinib). Symbols (single culture) bars (mean), and error bars (standard deviation). ns *p* >0.05, ∗*p* <0.05, ∗∗*p* <0.01, ∗∗∗*p* <0.001 (Šídák's multiple-comparisons test [cycling T cells in B, transitioning monocytes in C], Dunn’s multiple-comparisons test [cDCs in D]). Bar, baricitinib; cDC, conventional dendritic cell; CpG, cytosine-phosphate-guanine; IFN, interferon; STAT1, signal transducer and activator of transcription 1; WT, wild type.

To investigate whether type I and II IFNs stimulate the phenotype of the TLR9-associated hepatic leukocytes, cells from CpG-injected WT mice were cultured and stimulated with IFNs for 24 h (Fig. 7A). Using baricitinib, we also evaluated their dependence on STAT1 signaling. Both type I or II IFNs are able to promote the ‘cycling’ phenotype of CD8^+^ T cells and the ‘transitioning’ phenotype of monocytes *ex vivo* (Fig. 7B and C). However, in case of the cDC2s, IFNs do not promote their phenotype, suggestive of their indirect effect on cDC2s *in vivo* (Fig. 7D).

Taken together, using flow cytometry, we validated the expansion of cycling T cells and transitioning monocytes, which are both stimulated by type I and II IFNs and STAT1 signaling, as well as the expansion of cDC2s during TLR9 triggering in WT mice.

### Pharmacological targeting of both type I and II interferons prevents clinical features of TLR9-induced inflammation and the expansion of hepatic leukocyte populations

To confirm that both type I and II IFNs drive TLR9-associated features, we treated mice with blocking anti-IFNAR antibodies and a neutralizing anti-IFN-γ antibody (Fig. S6A).

**Fig. 8:**
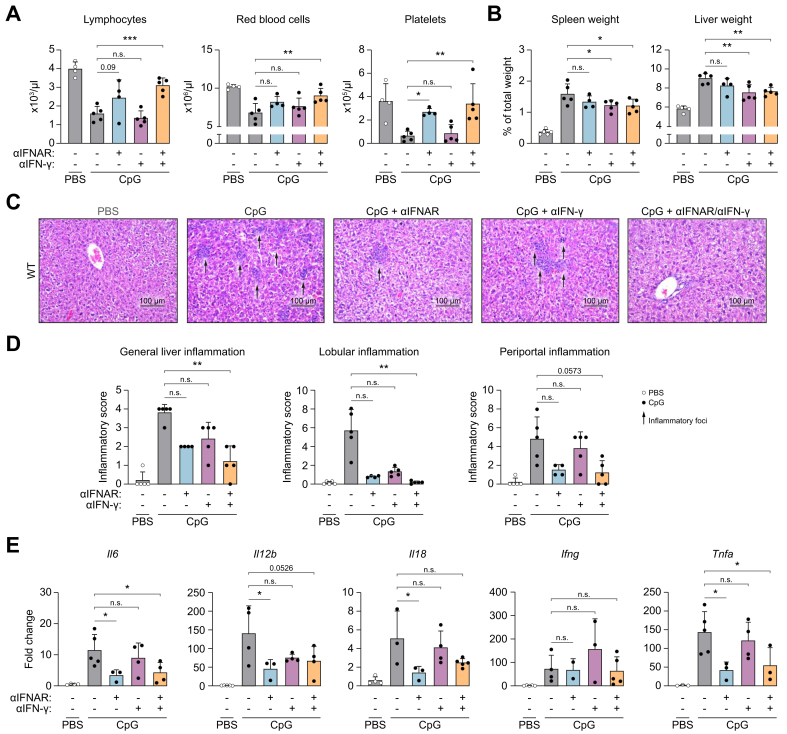
Pharmacological targeting of type I and II interferons prohibits TLR9-induced clinical features. Mice were treated with αIFNAR, αIFN-γ or both, as described in the Materials and methods and shown in experimental setup in Fig. S6A. (A) Blood counts. (B) Percentages of organ *vs.* body weight. (C) Representative liver sections with H&E staining and (C) quantification of liver inflammation. (E) Fold change expression total liver lysate. Bars (mean) and error bars (standard deviation). ns *p* >0.05, ∗*p* <0.05, ∗∗*p* <0.01, ∗∗∗*p* <0.001 [Šídák's multiple-comparisons test (A, B, E), Dunn’s multiple-comparisons test (D, E – Ifng)]. CpG, cytosine-phosphate-guanine; IFN, interferon; IFNAR, interferon-α/β receptor; TLR, Toll-like receptor.

CpG-injected WT mice receiving double treatment, show improvement in pancytopenia (Fig. 8A), hepatosplenomegaly (Fig. 8B), and liver inflammation (Fig. 8C and D), with reduced expression of inflammatory cytokines (*Il6*, *Il12b*, and *Tnfa*) (Fig. 8E) compared with untreated mice. In contrast, anti-IFN-γ treatment alone significantly improves hepatosplenomegaly (Fig. 8B–D), while single treatment with anti-IFNAR only improves thrombocytopenia and minimizes the expression of inflammatory cytokines (*Il6*, *Il12b*, and *Tnfa*).

Similarly, expansion of hepatic leukocyte populations associated with TLR9 triggering is more inhibited in CpG-injected WT mice with the combined treatment, compared with anti-IFN-γ antibody, hence mimicking the *Stat1*^*-/-*^ mice (Fig. 5, Fig. 6A–E and Figs S7A, S8A and C, S9A–C, and S11A–C).

In conclusion, simultaneous pharmacological inhibition of type I and II IFNs in CpG-injected WT mice phenocopies the protective phenotype of *Stat1*^*-/-*^ mice against TLR9-associated clinical features and hepatic immune populations.

### Type I and II IFN signature in blood and liver from patients with a TLR9-implicated pathology

To confirm the clinical relevance of our findings, we explored publicly available datasets of patients with a cytokine storm syndrome (MAS)^16^ and liver inflammation (primary sclerosing cholangitis [PSC], which is characterized by chronic inflammation and irreparable damage to the bile ducts).^23^ Importantly, both disorders are characterized by a TLR9-implicated pathology.^2^^,^^5^^,^^24^^,^^25^ In peripheral blood mononuclear cells (PBMCs) of patients with MAS, RNA-seq showed significantly enriched type I and II IFN signatures (Fig. S12A). Additionally, in liver non-parenchymal cells from patients with PSC, expression of type I and II ISGs, and STAT1 are significantly enriched in several leukocyte populations, including the cycling and CD8^+^ T cells, plasmacytoid dendritic cell (pDCs), monocytes, and KCs (Fig. S12B).

In conclusion, type I and II IFN signatures in blood and liver from patients with a TLR9-implicated pathology validate the clinical relevance of our findings with the murine TLR9 model.

## Discussion

Persistent TLR triggering initiates and perturbates a cytokine storm syndrome, a severe complication associated with infections, malignancies, and autoinflammatory disorders. Using a well-characterized model of TLR9-induced inflammation, our analysis demonstrated that type I and II IFN-induced STAT1 signaling drives the TLR9-associated liver inflammatory landscape, characterized by the presence of cycling CD8^+^ T cells, transitioning monocytes, and cDC2s.

The role of type I IFNs in TLR9-induced liver inflammation has been very scarcely investigated. Canna *et al.*^26^ demonstrated that *Ifnar*^*-/-*^ mice are partially protected from TLR9-associated clinical features, including mildly but significantly reduced liver inflammation compared with WT mice. As CpG-injected *Ifnar*^*-/-*^ mice still exhibited increased levels of type II IFNs,^26^ this may drive the remaining liver inflammation, which is in line with our findings using the anti-IFNAR antibody treatment. Correspondingly, Huang *et al.*^16^ recently identified both type I and II IFN signatures in PBMCs of patients with MAS. Additionally, in a publicly available dataset of hepatic leukocyte populations of patients with liver inflammation, we identified both type I and II IFN signatures, which further underscores the clinical relevance of our findings. It has been shown that type I IFNs prime the type II IFN-induced signaling pathway by upregulating the expression of STAT1.^27^ In agreement with this, we found increased STAT1 expression in all three TLR9-associated hepatic leukocyte populations of CpG-injected *Ifng*^*-/-*^ mice, in which type I IFN signaling is intact. Furthermore, Weaver *et al.*^28^ have shown that injecting healthy WT mice with solely type II IFNs could not recapitulate the clinical features of TLR9-induced inflammation. Instead, the interplay between type II IFNs and TLR9 signaling was required to induce systemic and hepatic inflammation.^28^ Based on our observations, we assume that type I IFNs induced by TLR9 triggering is particularly required. Interestingly, IFNAR and IFN-γ deficiency have been shown to protect against hepatic inflammation in other disease contexts, such as experimental severe malaria, and PBMCs of patients with malaria also exhibit a high type I IFN signature.^29^

We identified that TLR9-induced liver inflammation is dominated by a monocyte population differentiating into Mϕs, which we termed transitioning monocytes. STAT1 is significantly associated with their differentiation, which is in accordance with the established role of STAT1 in Mϕ differentiation and function.[30], [31], [32] Interestingly, blood monocytes of patients with cytokine storm syndrome (MAS) display a similar increase in basal levels of pSTAT1, and their monocytes are hyperresponsive to type II IFN stimulation, resulting in even higher pSTAT1 levels.^33^ Whether this type II IFN hyperresponsiveness is mediated by type I IFN priming was not explored by the authors. Verweyen *et al.*^34^ showed that TLR-induced IL-18 production from monocytes is strongly enhanced when primed with type I IFNs. In line with this, we found that anti-IFNAR, but not anti-IFN-γ treatment, inhibits IL-18 production in the liver upon TLR9 triggering. Furthermore, our transcriptome data showed that the CpG-induced transitioning monocytes and Mϕs both display high expression of *Il18*. In addition, we found increased expression of *Tnf* and, to a lesser extent, of *Il6*, which is reminiscent of the interleukin (IL)-6^+^ and TNF-α^+^ Mϕs found in liver biopsies of patients with cytokine storm syndrome (MAS).^35^

Regarding the origin of monocyte expansion, Weaver *et al.* showed that a CCR2-independent mechanism of extramedullary monocytopoiesis drives their expansion during TLR9 triggering.^36^ With both our cell isolation protocols, we show monocyte expansion, indicating that they are not merely enriched in the blood but also expand within the liver tissue itself. Additionally, we found the increased expression of Sca-1, a marker of murine hematopoietic stem cells,^20^ on the transitioning monocytes, further supporting the hypothesis of TLR9-induced extramedullary monocytopoiesis. In the same study, it was shown that inflammatory monocyte expansion is not altered in *Ifng*^*-/-*^ and *Ifnar*^*-/-*^ mice during TLR9 triggering,^36^ while we found their significant reduction in *Ifng*^*-/-*^ and *Stat1*^*-/-*^ mice or with anti-IFN-γ and anti-IFNAR treatment in WT mice.^36^ Nevertheless, inflammatory monocytes were defined as CCR2^+^Ly6C^hi^ cells, only representing a subset of monocytes, whereas we defined our transitioning monocytes as Ly6C^+^F4/80^+^ based on our CITE-seq results.

With our *ex vivo* digestion protocol, we found that liver-resident KCs are depleted during TLR9-induced liver inflammation. Instead, another KC population is induced, expressing CLEC2 but not VSIG4, and therefore reminiscent of pre-moKCs, which are recruited to replenish the KC pool.^37^ This so-called ‘Mϕ disappearance reaction’ has been described in a variety of inflammatory liver disorders.[38], [39], [40], [41], [42] Interestingly, type I IFNs have been implicated in delaying KC replenishment during viral hepatitis.^38^

Recent patient studies described a peripheral CD38^+^HLA-DR^+^CD8^+^ T cell population,^43^^,^^44^ with increased expression of genes implicated in cell proliferation in patients with cytokine storm syndrome (MAS).^16^ Likewise, we found that TLR9-associated hepatic CD8^+^ T cells express CD38 and display an overall gene signature that strongly resembles those of patient cells. With our CITE-seq data, we found that cycling and non-cycling T cells, as well as natural killer (NK) cells, are the main IFN-γ-producing cells. Additionally, Rood *et al.*^45^ identified a unique dual IL-10- and IFN-γ-producing CD8^+^ T_eff_ cell population with high turnover in livers of CpG-injected WT mice. Similarly, we found *Il10* expression in the TLR9-associated (non-)cycling CD8^+^ T_eff_ cell populations, suggesting that they correspond to this population. Interestingly, induction of IL-10 was shown to be antigen-independent but partially type II IFN-dependent.^45^ Whether type I IFNs are the remaining factor mediating their expansion was not evaluated by the authors. However, Huang *et al.*^16^ demonstrated the importance of type I IFNs, as *in vitro* stimulation of healthy donor CD8^+^ T cells with IFN-α2 resulted in the generation of CD38^+^HLA-DR^+^CD8^+^ T cells.

Data on cDCs in TLR9-induced liver inflammation are scarce, and their role in patients with cytokine storm syndrome remains unclear. Behrens *et al.*^5^ suggested a role for cDCs in the early production of type II IFNs, with late-phase production taken over by NK and T cells, as we confirm here. Within the DC population, we identified that cDC2s are particularly expanding during TLR9 triggering. Interestingly, cDC2s have been described to provoke a proinflammatory environment and aid in the recruitment of Mϕs into the liver.^46^ Additionally, STAT1 gain-of-function has been shown to skew DCs to a proinflammatory phenotype and causes them to lose their tolerogenic properties.^47^ Therefore, the excessive STAT1 activation during TLR9 triggering may induce such an inflammatory phenotype.

In summary, we show that type I and II IFN-induced STAT1 activation drives TLR9-associated features, particularly liver inflammation, which is characterized by IFN-γ-producing cycling T cells, cDC2s, and monocytes transitioning into inflammatory Mϕs. Our findings further support the therapeutic use of JAK1/2 inhibition in patients with refractory cytokine storm syndromes. Moreover, we highlight the potential of novel therapeutic strategies beyond type II IFNs neutralization such as targeting type I IFNs or safely inhibiting STAT1. Given that STAT1 acts downstream from JAKs, its inhibition may offer a more refined approach with potentially fewer off-target effects than broad JAK blockade. Nonetheless, further preclinical research on these therapies is warranted to ensure their safe application in the clinic.

## Abbreviations

ALT, alanine aminotransferase; cDC, conventional dendritic cell; CpG, cytosine-phosphate-guanine; DC, dendritic cell; DGE, differential gene expression; DPE, differential protein expression; GSEA, gene set enrichment analysis; HRP, horseradish peroxidase; i.p., intraperitoneal; IFN, interferon; IFNAR, interferon-α/β receptor; ISG, IFN-stimulated gene; JAK, Janus kinase; KC, Kupffer cell; MAS, macrophage activation syndrome; Mϕ, macrophage; NK, natural killer; PBMCs, peripheral blood mononuclear cells; pDC, plasmacytoid dendritic cell; PSC, primary sclerosing cholangitis; pSTAT1, phosphorylated signal transducer and activator of transcription 1; qRT-PCR, quantitative reverse transcription-polymerase chain reaction; STAT1, signal transducer and activator of transcription 1; TBST, tris-buffered saline Tween; T_eff_, effector T; TLR, Toll-like receptor; WT, wild-type.

## Authors’ contributions

Conceptualization: ADV, JB, BM-D, FP, CHW, PM. Resources: PM. Methodology: ADV, JB, EB, MV, SA, OG, JL, NV, AB, CLS. Investigation: ADV, JB, EB, MV, FP, HM, LS, TM, NB, SA, OG, JL, NV. Formal analysis: ADV. Supervision: CHW, PM. Project administration: PM. Writing – original draft: ADV. Writing – review and editing: JB, EB, MV, BM-D, FP, PVS, AB, CLS, CHW, PM. Funding acquisition: ADV, JB, EB, CHW, PM.

## Data availability

All data that support the main findings in this study are available in the manuscript or the supplementary materials. Raw data supporting the conclusions in this manuscript will be made available by the authors, without undue reservation, to any qualified researcher. The corresponding author (PM) can be contacted via e-mail (patrick.matthys@kuleuven.be) and will provide files by written request. The raw transcriptomics data has been uploaded to CBI’s Gene Expression Omnibus (GEO) repository under the accession number GSE287998.

## Financial support

This work was supported by grants from the 10.13039/501100003130Research Foundation Flanders (FWO) (G0A3218N) and KU Leuven (C1 grants no. C16/17/010 and C14/23/143). ADV, JB, and EB received an FWO fellowship for fundamental research (11K0722N and 11K0724N [to ADV], 11A0523N and 11A0525N [to JB], 11H9123N and 11H9125N [to EB]).

## Conflicts of interest

CW has received consultancy fees (Novartis, Sobi, and UCB) and unrestricted grants from Novartis, Roche, GlaxoSmithKline, and Pfizer paid to her institution. The authors have no additional financial interests.

Please refer to the accompanying ICMJE disclosure forms for further details.

## References

[1] Vanderbeke L., Van Mol P., Van Herck Y. (2021). Monocyte-driven atypical cytokine storm and aberrant neutrophil activation as key mediators of COVID-19 disease severity. Nat Commun.

[2] Brisse E., Wouters C.H., Matthys P. (2016). Advances in the pathogenesis of primary and secondary haemophagocytic lymphohistiocytosis: differences and similarities. Br J Haematol.

[3] Yanagimachi M., Naruto T., Miyamae T. (2011). Association of IRF5 polymorphisms with susceptibility to macrophage activation syndrome in patients with juvenile idiopathic arthritis. J Rheumatol.

[4] Fall N., Barnes M., Thornton S. (2007). Gene expression profiling of peripheral blood from patients with untreated new-onset systemic juvenile idiopathic arthritis reveals molecular heterogeneity that may predict macrophage activation syndrome. Arthritis Rheumatol.

[5] Behrens E.M., Canna S.W., Slade K. (2011). Repeated TLR9 stimulation results in macrophage activation syndrome-like disease in mice. J Clin Invest.

[6] De Benedetti F., Grom A.A., Brogan P.A. (2023). Efficacy and safety of emapalumab in macrophage activation syndrome. Ann Rheum Dis.

[7] Hu X., li J., Fu M. (2021). The JAK/STAT signaling pathway: from bench to clinic. Signal Transduct Target Ther.

[8] Prencipe G., Bracaglia C., Caiello I. (2019). The interferon-gamma pathway is selectively up-regulated in the liver of patients with secondary hemophagocytic lymphohistiocytosis. PLoS One.

[9] Song Z., Yao H., Jin Y. (2024). Ruxolitinib as a salvage therapy in adult-onset macrophage activation syndrome: insights from eight cases. Ann Rheum Dis.

[10] Liao J., Tang Q., Xie X. (2024). The efficacy and safety of JAK inhibitors in patients with adult-onset Still’s disease: a meta-analysis and systematic review. Int Immunopharmacol.

[11] Levy O., Apel A., Alhdor H. (2023). Ruxolitinib for refractory macrophage activation syndrome complicating adult-onset Still’s disease. Eur J Rheumatol.

[12] Albeituni S., Verbist K.C., Tedrick P.E. (2019). Mechanisms of action of ruxolitinib in murine models of hemophagocytic lymphohistiocytosis. Blood.

[13] De Visscher A., Vandeput M., Vandenhaute J. (2024). Liver type 1 innate lymphoid cells undergo apoptosis in murine models of macrophage activation syndrome and are dispensable for disease. Eur J Immunol.

[14] Scott C.L., Zheng F., De Baetselier P. (2016). Bone marrow-derived monocytes give rise to self-renewing and fully differentiated Kupffer cells. Nat Commun.

[15] De Visscher A., Vandeput M., Malengier-Devlies B. (2025). Upregulation of Fcγ receptor IV on activated monocytes and macrophages causes nonspecific binding of the PK136 Anti-NK1.1 antibody in murine models of Toll-like receptor-induced inflammation. Scand J Immunol.

[16] Huang Z., Brodeur K.E., Chen L. (2023). Type I interferon signature and cycling lymphocytes in macrophage activation syndrome. J Clin Invest.

[17] Villarino A.V., Kanno Y., O’Shea J.J. (2017). Mechanisms and consequences of Jak-STAT signaling in the immune system. Nat Immunol.

[18] Guilliams M., Bonnardel J., Haest B. (2022). Spatial proteogenomics reveals distinct and evolutionarily conserved hepatic macrophage niches. Cell.

[19] Blasius A.L., Giurisato E., Cella M. (2006). Bone marrow stromal cell antigen 2 is a specific marker of type I IFN-producing cells in the naive mouse, but a promiscuous cell surface antigen following IFN stimulation. J Immunol.

[20] Holmes C., Stanford W.L. (2007). Concise review: stem cell antigen-1: expression, function, and enigma. Stem Cells.

[21] Mahalingam S., Chaudhri G., Tan C.L. (2001). Transcription of the interferon γ (IFN-γ)-inducible chemokine Mig in IFN-γ-deficient mice. J Biol Chem.

[22] Swiecki M., Colonna M. (2010). Unraveling the functions of plasmacytoid dendritic cells during viral infections, autoimmunity, and tolerance. Immunol Rev.

[23] Andrews T.S., Nakib D., Perciani C.T. (2024). Single-cell, single-nucleus, and spatial transcriptomics characterization of the immunological landscape in the healthy and PSC human liver. J Hepatol.

[24] Matsushita H., Miyake Y., Takaki A. (2015). TLR4, TLR9, and NLRP3 in biliary epithelial cells of primary sclerosing cholangitis: relationship with clinical characteristics. J Gastroenterol Hepatol.

[25] Karrar A., Broomé U., Södergren T. (2007). Biliary epithelial cell antibodies link adaptive and innate immune responses in primary sclerosing cholangitis. Gastroenterology.

[26] Canna S.W., Wrobel J., Chu N. (2013). Interferon-γ mediates anemia but is dispensable for fulminant Toll-like receptor 9-induced macrophage activation syndrome and hemophagocytosis in mice. Arthritis Rheum.

[27] Gough D.J., Messina N.L., Hii L. (2010). Functional crosstalk between type I and II interferon through the regulated expression of STAT1. PLoS Biol.

[28] Weaver L.K., Chu N., Behrens E.M. (2019). Brief Report: interferon-γ–mediated immunopathology potentiated by Toll-like receptor 9 activation in a murine model of macrophage activation syndrome. Arthritis Rheumatol.

[29] Dooley N.L., Chabikwa T.G., Pava Z. (2023). Single cell transcriptomics shows that malaria promotes unique regulatory responses across multiple immune cell subsets. Nat Commun.

[30] Lawrence T., Natoli G. (2011). Transcriptional regulation of macrophage polarization: enabling diversity with identity. Nat Rev Immunol.

[31] Wang N., Liang H., Zen K. (2014). Molecular mechanisms that influence the macrophage M1-M2 polarization balance. Front Immunol.

[32] Coccia E.M., Russo N Del, Stellacci E. (1999). STAT1 activation during monocyte to macrophage maturation: role of adhesion molecules. Int Immunol.

[33] Pascarella A., Bracaglia C., Caiello I. (2021). Monocytes from patients with macrophage activation syndrome and secondary hemophagocytic lymphohistiocytosis are hyperresponsive to interferon gamma. Front Immunol.

[34] Verweyen E., Holzinger D., Weinhage T. (2020). Synergistic signaling of TLR and IFNα/β facilitates escape of IL-18 expression from endotoxin tolerance. Am J Respir Crit Care Med.

[35] Billiau A.D., Roskams T., Van Damme-Lombaerts R. (2005). Macrophage activation syndrome: characteristic findings on liver biopsy illustrating the key role of activated, IFN-γ-producing lymphocytes and IL-6- and TNF-α-producing macrophages. Blood.

[36] Weaver L.K., Chu N., Behrens E.M. (2016). TLR9-mediated inflammation drives a Ccr2-independent peripheral monocytosis through enhanced extramedullary monocytopoiesis. Proc Natl Acad Sci USA.

[37] Tran S., Baba I., Poupel L. (2020). Impaired Kupffer cell self-renewal alters the liver response to lipid overload during non-alcoholic steatohepatitis. Immunity.

[38] Borst K., Frenz T., Spanier J. (2018). Type I interferon receptor signaling delays Kupffer cell replenishment during acute fulminant viral hepatitis. J Hepatol.

[39] Blériot C., Dupuis T., Jouvion G. (2015). Liver-resident macrophage necroptosis orchestrates type 1 microbicidal inflammation and type-2-mediated tissue repair during bacterial infection. Immunity.

[40] Louwe P.A., Badiola Gomez L., Webster H. (2021). Recruited macrophages that colonize the post-inflammatory peritoneal niche convert into functionally divergent resident cells. Nat Commun.

[41] Devisscher L., Scott C.L., Lefere S. (2017). Non-alcoholic steatohepatitis induces transient changes within the liver macrophage pool. Cell Immunol.

[42] Zigmond E., Samia-Grinberg S., Pasmanik-Chor M. (2014). Infiltrating monocyte-derived macrophages and resident Kupffer cells display different ontogeny and functions in acute liver injury. J Immunol.

[43] De Matteis A., Colucci M., Rossi M. (2020). Expansion of CD4dimCD8+ T cells characterizes macrophage activation syndrome and other secondary HLH. Blood.

[44] Chaturvedi V., Marsh R.A., Zoref-Lorenz A. (2021). T-cell activation profiles distinguish hemophagocytic lymphohistiocytosis and early sepsis. Blood.

[45] Rood JE, Canna SW, Weaver LK, *et al.* IL-10 distinguishes a unique population of activated, effector-like CD8+ T cells in murine acute liver inflammation. J Leukoc Biol;101:1037–1044. 10.1189/jlb.3A0916-221RR PMC5346180 28034913

[46] Méndez-Sánchez N., Córdova-Gallardo J., Barranco-Fragoso B. (2021). Hepatic dendritic cells in the development and progression of metabolic steatohepatitis. Front Immunol.

[47] Parackova Z., Zentsova I., Vrabcova P. (2023). Aberrant tolerogenic functions and proinflammatory skew of dendritic cells in STAT1 gain-of-function patients may contribute to autoimmunity and fungal susceptibility. Clin Immunol.

