# Supplementary material for: STAT1 drives the immune landscape of murine Toll-like receptor 9-induced liver inflammation
Source: JHEP Rep. 2025 Nov 6;8(2):101668. doi: 10.1016/j.jhepr.2025.101668 (PMC12814853; doi:10.1016/j.jhepr.2025.101668)
Supplement: Multimedia component 1 [file mmc1.docx]

**STAT1 drives the immune landscape of murine Toll-like receptor 9- induced liver inflammation**

Amber De Visscher, Jarne Beliën, Eline Bernaerts, Marte Vandeput, Bert Malengier-Devlies, Fran Prenen, Hanne Meers, Liliana Sokol, Tania Mitera, Nele Berghmans, Seray Anak, Olivier Govaere, Philippe Van den Steen, Jochen Lamote, Niels Vandamme, Anna Bujko, Charlotte L. Scott, Carine H. Wouters, Patrick Matthys

**Table of contents**

Methods……………………………………………………………………………………2

Supplementary figures…………………………………………………………………...4

Supplementary tables…………………………………………………………………..18

Supplementary references……………………………………………………………..23

#

# ****Methods****

## **Mice**

WT C57BL/6 mice were purchased from Janvier Laboratories or bred at the Animal Centre of the Rega Institute, KU Leuven. *Ifng^-/-^* C57BL/6 mice were purchased from The Jackson Laboratory, and *Stat1^-/-^* C57BL/6 mice were kindly provided by Dr. Andrew Brown and Dr. Andy Wullaert (VIB-University of Ghent), and further bred at the Animal Centre of the Rega Institute, KU Leuven.

## **Scoring of liver inflammation**

Liver sections were blindly score and the following hepatic inflammatory scores were defined: general liver inflammation (0; no inflammation, 1; sporadic inflammation, 2; mild inflammation, 3; moderate inflammation and 4; severe inflammation), lobular inflammation (average number of inflammatory foci/mm^2^ counted in 10x 1mm^2^ squares and periportal inflammation (number of inflamed hepatic portal veins out of 10).

## **Protein extraction and western blot**

For protein extraction, tissue or cells were lysed with RIPA buffer (TCI Chemicals) containing protease (Roche) and phosphatase inhibitor cocktails (Sigma-Aldrich). Of each sample, 25 µg of protein was resuspended in Pierce™ reducing loading buffer (Thermo Fisher Scientific) and incubated for 10 min at 80°C. Samples were separated in 4-12% Tris-Glycine gels (precast Novex Tris-Glycine gels, ThermoFisher Scientific). Proteins in gels were transferred onto PVDF membranes using the Trans-Blot Turbo Transfer system with associated materials and protocols.

## **Single-cell transcriptomics**

### *Preprocessing of sequencing data and quality control*

The Cell Ranger toolkit (v6.0.0) was used for demultiplexing, alignment to the GRCm38.99 mouse reference genome, and decoding of the CITE-seq barcodes. Before integration, the four samples went separately through the QC pipeline in R (v4.2.2). Genes present in fewer than five cells were removed, and only high-quality cells, defined as cells expressing at least 200 genes, were retained. Additionally, thresholds for the minimum and maximum unique molecular identifier (UMI) content per cell and the maximum mitochondrial RNA content were determined automatically using the Scater package with default parameters (v1.26.1). Cells that did not meet these criteria were filtered out. The corrected gene count matrices, antibody-derived tags (ADT) count matrices, and relevant metadata were used to create a single cell experiment (sce) object with the SingleCellExperiment package (v1.20.1). Doublets were identified via scDblFinder (v1.12.0) with default parameters and removed from the sce objects, which were then converted into Seurat objects with the Seurat package (v4.4.0) for further analysis. The count matrices of each Seurat object were normalized and scaled using SCTransform (v0.4.1) with default parameters, and mitochondrial content was regressed out.

### *Data integration, batch effect removal, and cluster annotation*

To avoid sample-specific clusters, we integrated the gene count matrices of the individual samples using Seurat integration (v4.4.0) with default parameters, treating each sample as a batch. This process was repeated for the ADT count matrix. For downstream subclustering, the desired cell cluster(s) were selected and put into a new Seurat object, and the above-mentioned procedure was repeated. To cluster the cells, we ran RunPCA(), FindNeighbours(), and FindClusters(). The clusters were visualized on a Uniform Manifold Approximation and Projection (UMAP) plot. Optimal cluster resolution was determined with guidance from the clustree (v0.5.1) package. Differential gene expression (DGE) analysis and differential protein expression (DPE) were performed using FindAllmarkers() with default parameters to identify the most differentially expressed genes and proteins in each cluster. To annotate the clusters, we checked the expression of canonical marker genes and proteins (**Supplementary Figure S1A-B**). Furthermore, we used SingleR (v2.0.0) for automated annotation based on ImmGen reference, which consists of microarray profiles of pure mouse immune cells from the Celldex package (v1.8.0), to sanity check our manual annotations.

### *Correction for the enrichment of F4/80+ cells*

To show the correct percentages without enrichment in **Figure 2C** and **Figure 4B***,* we diminished the total counts of the F4/80^+^ cells (‘Transitioning monocytes’ and ‘Macrophages’) with 1,4% (1/70). Based on these corrected counts, the corresponding percentages were calculated.

### *Downstream analyses*

Differential composition analysis was performed using the mixed-effects association testing for single cells (MASC) package (0.1.0-alpha). The Odds ratio (OR) was calculated using a general linearized mixed-effects model with condition as the contrast of interest, cell cycle phase as a random-effect covariate, and the number of genes detected as a fixed-effect covariate. To perform pseudotime trajectory analysis on the myeloid compartment, we used Slingshot (v2.6.0). Since non-monocyte/Mφ clusters (“Migratory dendritic cells”, “Type 1 conventional dendritic cells”, “Type 2 conventional dendritic cells”, and “Plasmacytoid dendritic cells”) might interfere with the trajectories, we subsetted our data to only include monocyte/Mφ clusters for the trajectory analysis. Additionally, we carried out DGE analysis along the inferred trajectories with fitGAM(), assoRes(), and startVsEndTest() from the TradeSeq package (v1.12.0). The module score for the ‘CD38^+^HLA-DR^+^ cycling lymphocyte’ signature of patients with macrophage activation syndrome (MAS), a cytokine storm syndrome developing in the context of rheumatic disorders, from Huang *et al*.^16^ was calculated with AddModuleScore() using the top 10 enriched genes: *Cenpf*, *Gapdh*, *Gzma*, *Hmgb2*, *Hmgn2*, *Mki67*, *Pclaf*, *Stmn1*, *Tuba1b,* and *Tyms*.

## **Statistics**

Differential gene/protein expression data were tested for statistical significance using a Wilcoxon rank sum test with Bonferroni correction. Proteins were highlighted if *P*<0.05 with a minimal increase (red) or decrease (blue) of log2(FC)=0.322 (**Fig. 4G**). Differential expression of module scores across clusters was demonstrated with a Kruskal-Wallis test followed by a Dunn’s test for multiple comparisons with Benjamini-Hochberg correction. For GSEA, the wilcoxauc function (Presto) was used to calculate the Area Under the (receiver operator) Curve (AUC) values as an alternative to adjusted p-values. For the DGE analyses across trajectories, statistical significance was evaluated using a Wald test with Benjamini-Hochberg correction for multiple testing. To determine significant changes in the composition of the ORs as calculated by MASC, a general linearized mixed-effects model with Bonferroni correction was used.

## **Publicly available human datasets**

The GSEA of the RNA-seq data set of PBMCs from MAS and HC patients was republished with permission from the authors (Fig. 1C of Huang *et al*.^1^). To evaluate the expression of type I and II IFN signatures in PSC patients from the dataset of Andrews *et al.*^2^, we plotted the expression of established type I^3^ and II^4^ IFN gene sets using their online visualization tool [(https://macparlandlab.shinyapps.io/shiny_sc/](https://kuleuven-my.sharepoint.com/personal/amber_devisscher_kuleuven_be/Documents/Personal/7.%20Papers/3.%20Type%20I%20and%20II%20Interferons%20drive%20inflammation%20through%20STAT1%20in%20a%20murine%20model%20of%20macrophage%20activation%20syndrome/5.%20Submission%20J%20Hep%20Rep/Revision/(https:/macparlandlab.shinyapps.io/shiny_sc/)).

# ****Supplementary Figures****

## **Suppl. Fig. S1**

**
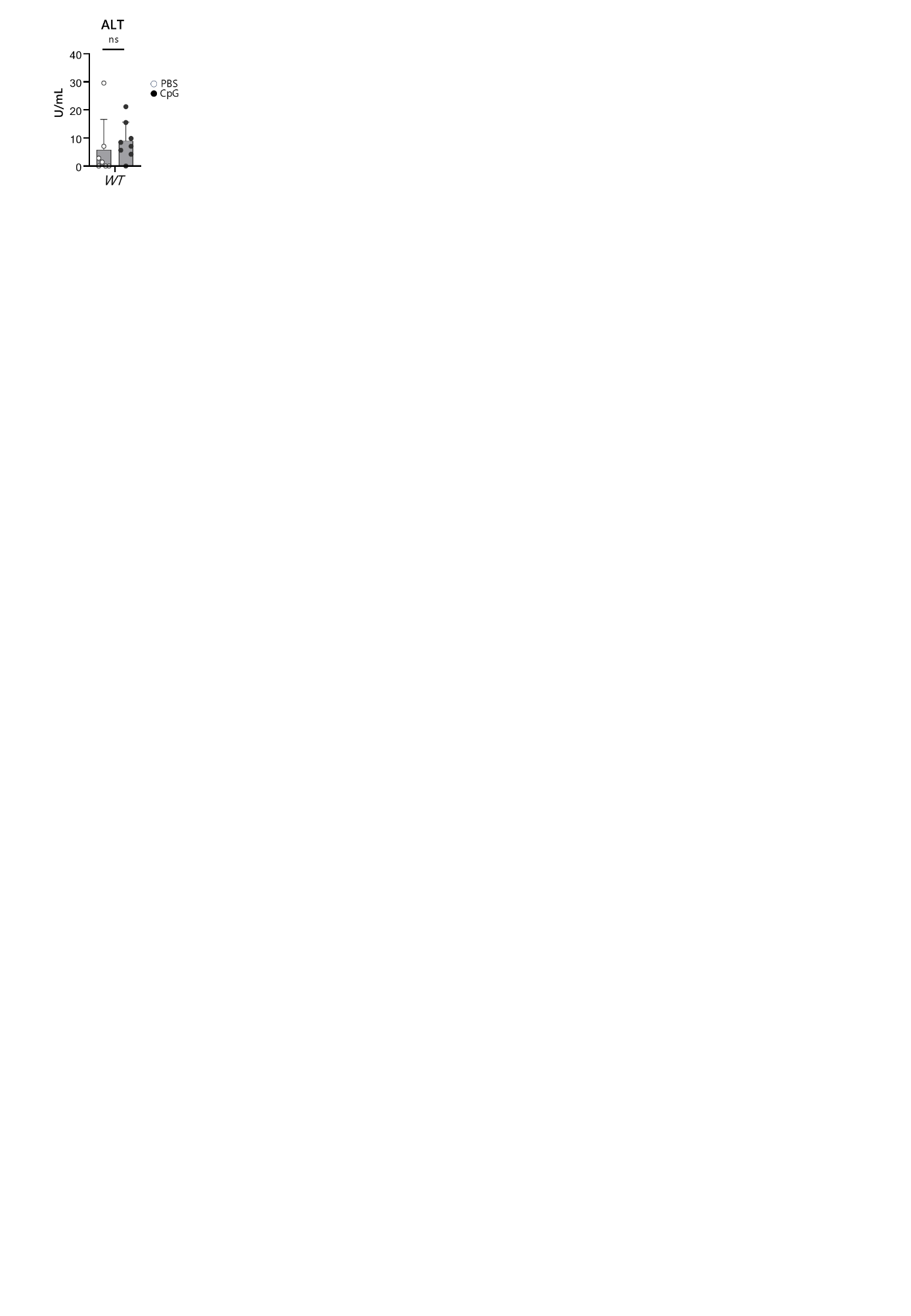
Suppl. Fig. S1. TLR9 triggering represents a model for liver inflammation without hepatotoxicity.** ALT levels in serum. Bars (mean) and error bars (standard deviation). ns *P*>0.05 [Mann-Whitney U-test].

## **Suppl. Fig. S2**

**
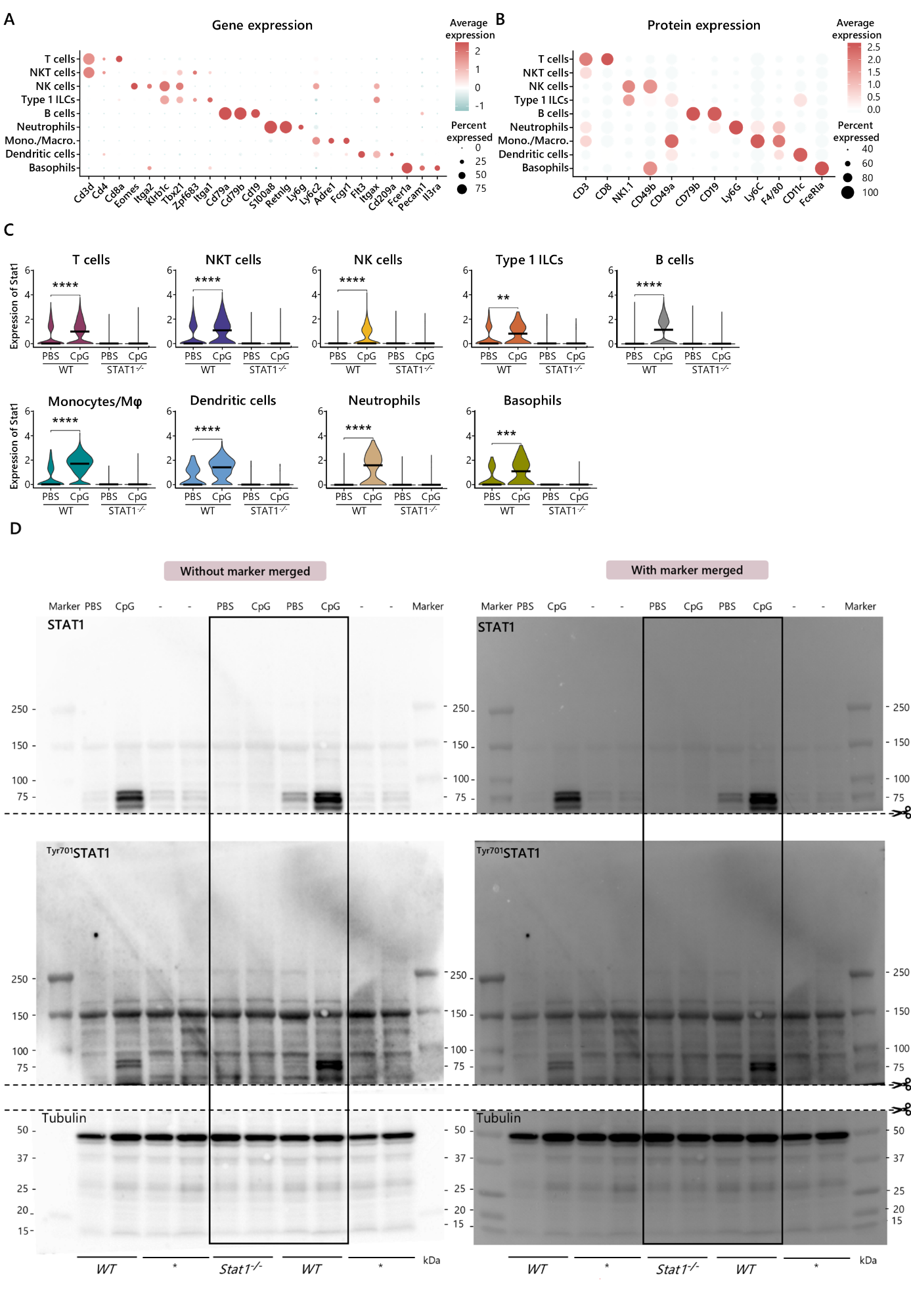
**

**Suppl. Fig. S2. Validation of the general cluster annotation and visualization of *Stat1* expression in the different clusters.** Experimental setup (**Fig. 2A**). Expression of canonical (A) genes and (B) protein markers of all clusters of the integrated dataset. (C) *Stat1* expression. Colors (clusters **Fig. 2B**). ***P*<0.01, ****P*<0.001 and *****P*<0.0001 [Kruskal-Wallis test followed by a Dunn’s test for multiple comparisons with Benjamini-Hochberg correction (C)]. (D) Representative uncropped western blots of pSTAT1, STAT1, and Tubulin (un)merged with the marker (ladder). The results shown in **Fig. 2G** are boxed. *; Samples that are not relevant for this project.

## **Suppl. Fig. S3**

**
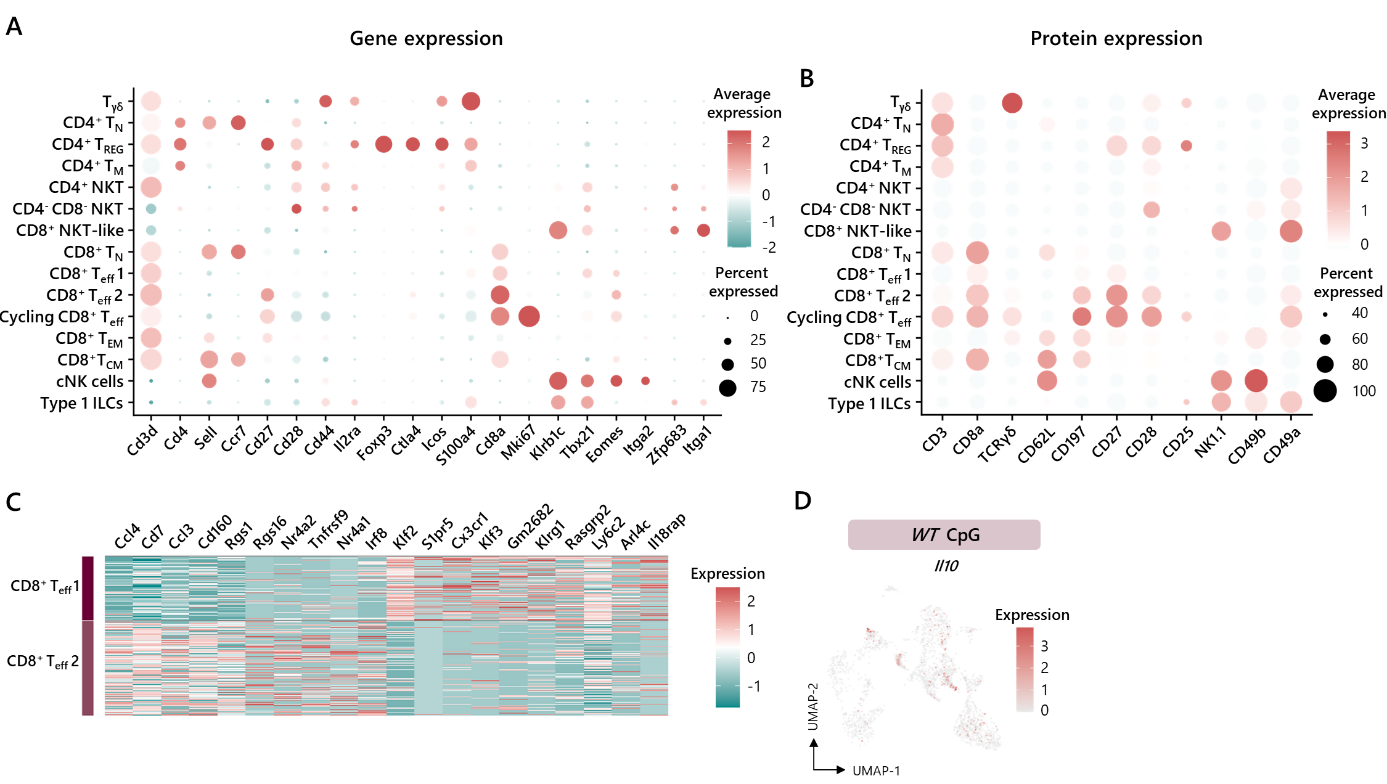
Suppl. Fig. S3. Validation of the lymphoid cluster annotation and DGE of the two different TLR9-associated CD8^+^ effector T cells.** Experimental setup (**Fig. 2A**). Expression of canonical (A) genes and (B) protein markers of all clusters of the lymphoid subclustering of the integrated dataset. (C) Top ten differentially expressed genes of CD8^+^ T_eff_ 1 versus CD8^+^ T_eff_ 2 of the lymphoid subclustering in *WT* CpG. Colors (clusters Fig. 2B) and color scale (average log-normalized expression). (D) *Il10* expression.

## **Suppl. Fig. S4**

**
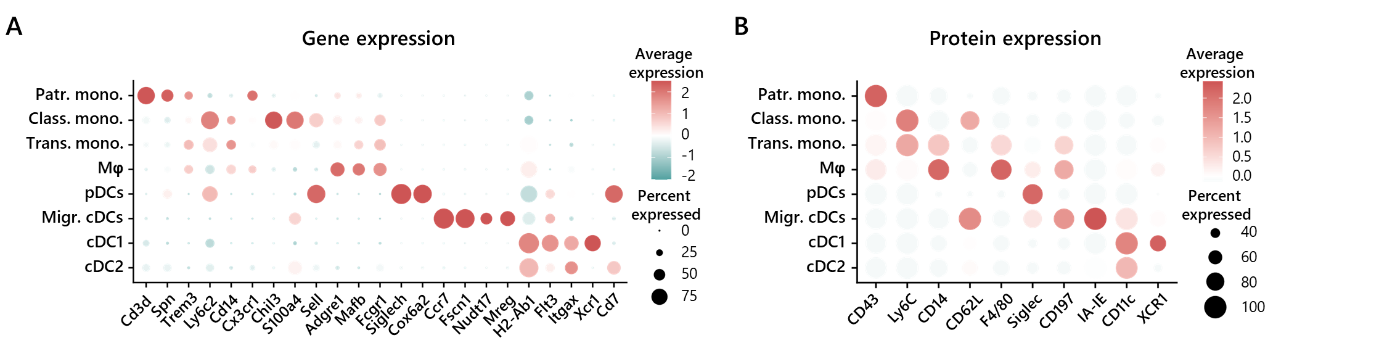
Suppl. Fig. S4. Validation of the myeloid cluster annotation.** Experimental setup (**Fig. 2A**). Expression of canonical (A) genes and (B) protein markers of all clusters of the myeloid subclustering of the integrated dataset. Dot size (percentage of expressing cells) and color scale (average log-normalized expression).

## **Suppl. Fig. S5**

**
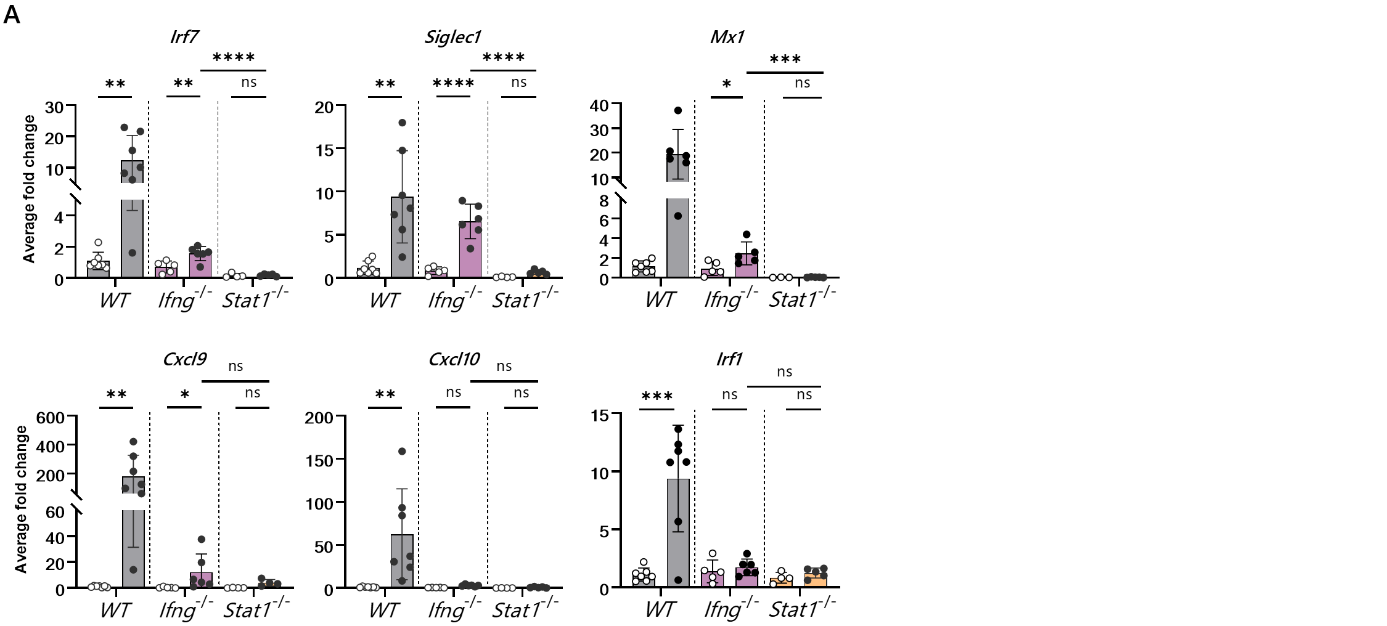
Suppl. Fig. S5. Induction of type I and type II IFN-stimulated genes during TLR9-mediated liver inflammation.** Experimental setup (**Fig. 1A**). Fold change in expression of interferon-stimulated genes in total liver lysate. Bars (mean) and error bars (standard deviation). ns *P*>0.05, **P*<0.05, ***P*<0.01, *****P*<0.0001 [Student’s t-test (*WT* in *Siglec1*, *Mx1*, Cxcl9, iRf1 ), Mann-Whitney U-test (*WT* in *Irf7*, *Cxcl10*), Šídák's multiple-comparisons test (*Ifng^-/-^*, *Stat1^-/-^* in *Irf7*, *Siglec1*, *Mx1*, *Cxcl10*, *Irf1*), Dunn’s multiple-comparisons test (*Ifng^-/-^*, *Stat1^-/-^* in *Cxcl9*)].

## **Suppl. Fig. S6**

**
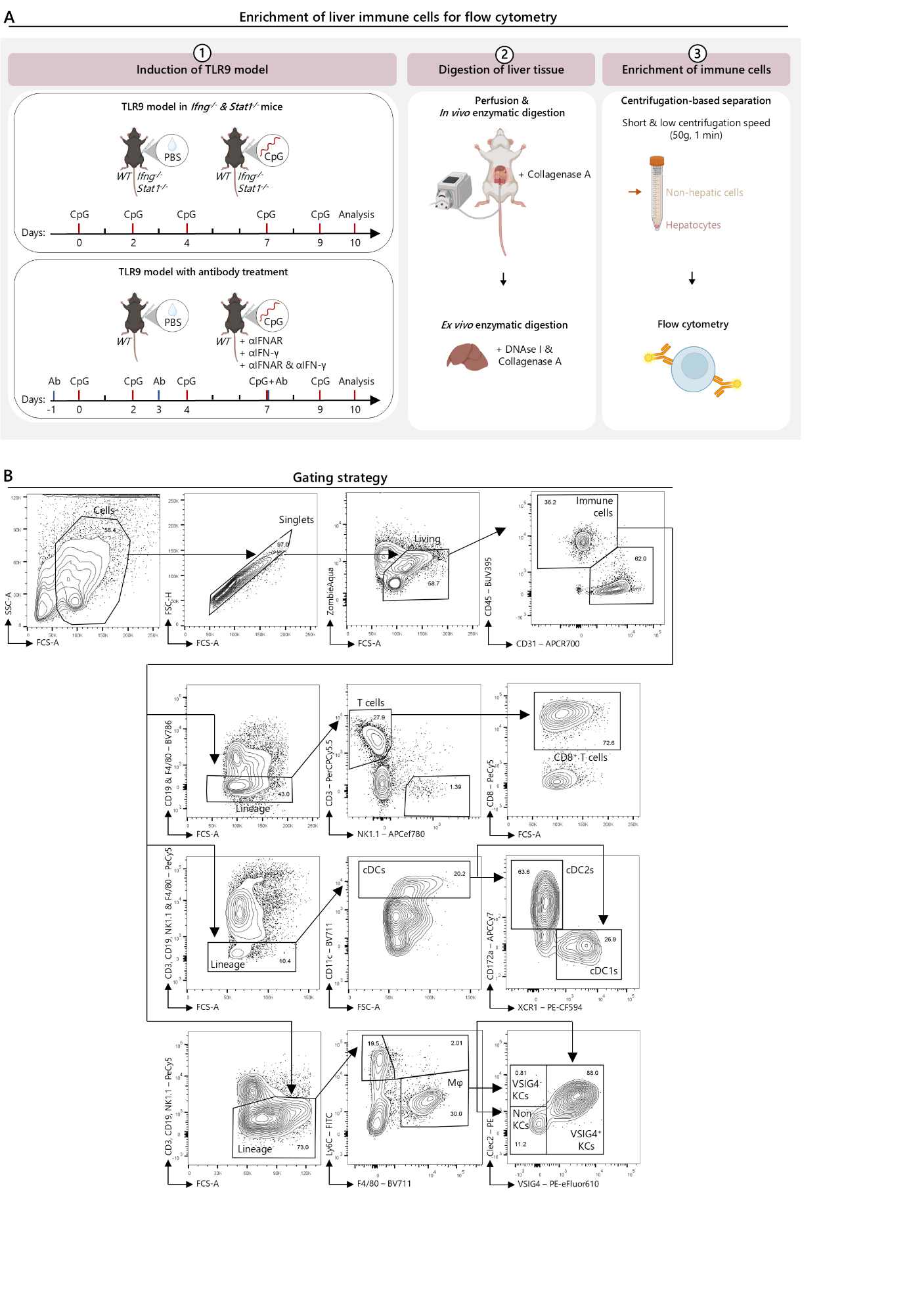
 Suppl. Fig. S6. Overview of the experimental setup for flow cytometry and gating strategies.** (A) Experimental setup. (B) Representative gating strategy of the TLR9-associated populations.

## **Suppl. Fig. S7**

**
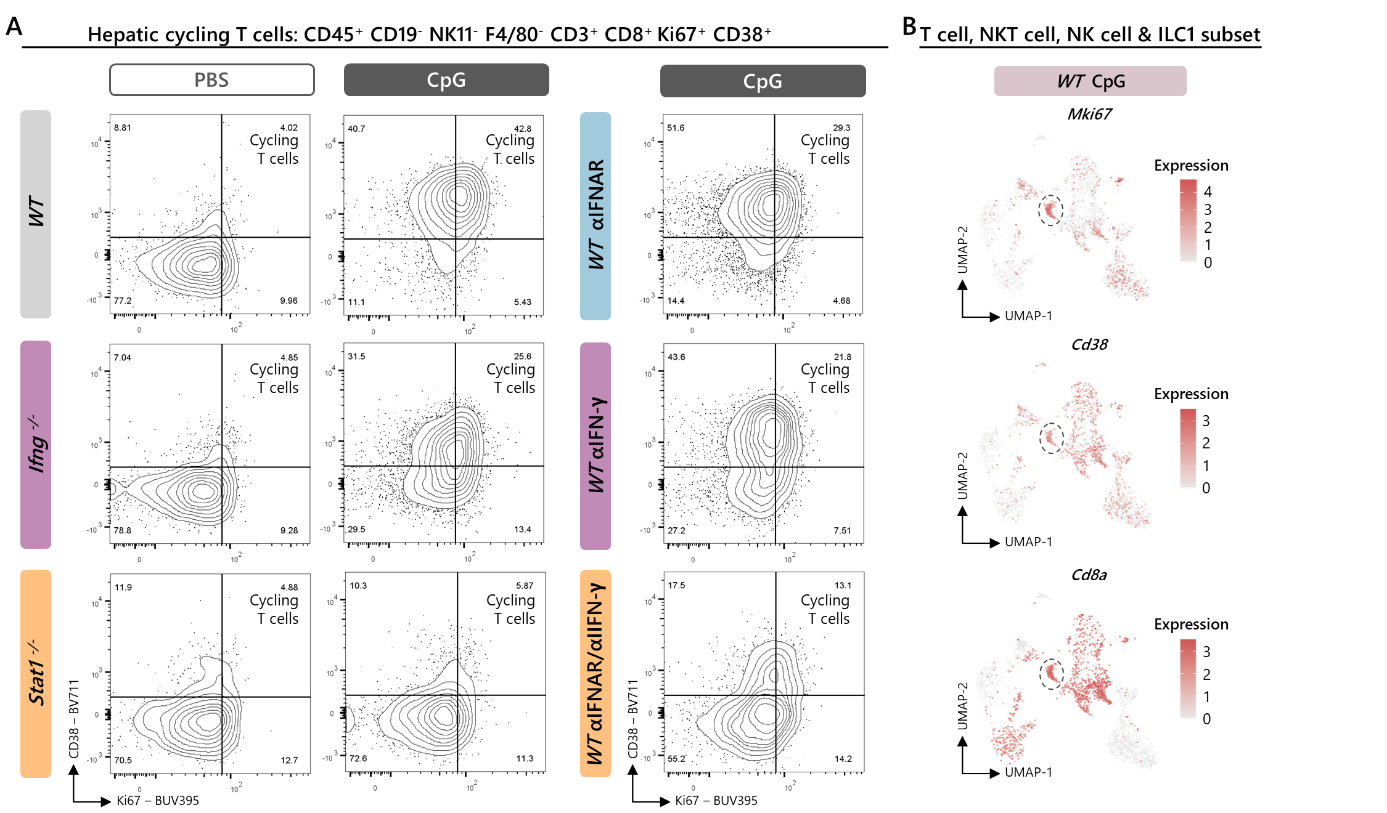
 Suppl. Figure S7. Comparison of cycling T cells identified by flow cytometry *versus* CITE-seq.** Experimental setup and gating strategy (**Suppl. Fig. S6**). (A) Representative flow cytometry plots of cycling T cells (CD45^+^CD19^-^NK1.1^-^F4/80^-^CD3^+^CD8^+^Ki67^+^CD38^+^ cells). (B) Lymphoid subclustering of *WT* CpG. Color scale (average log-normalized gene expression).

## **Suppl. Fig. S8**

**
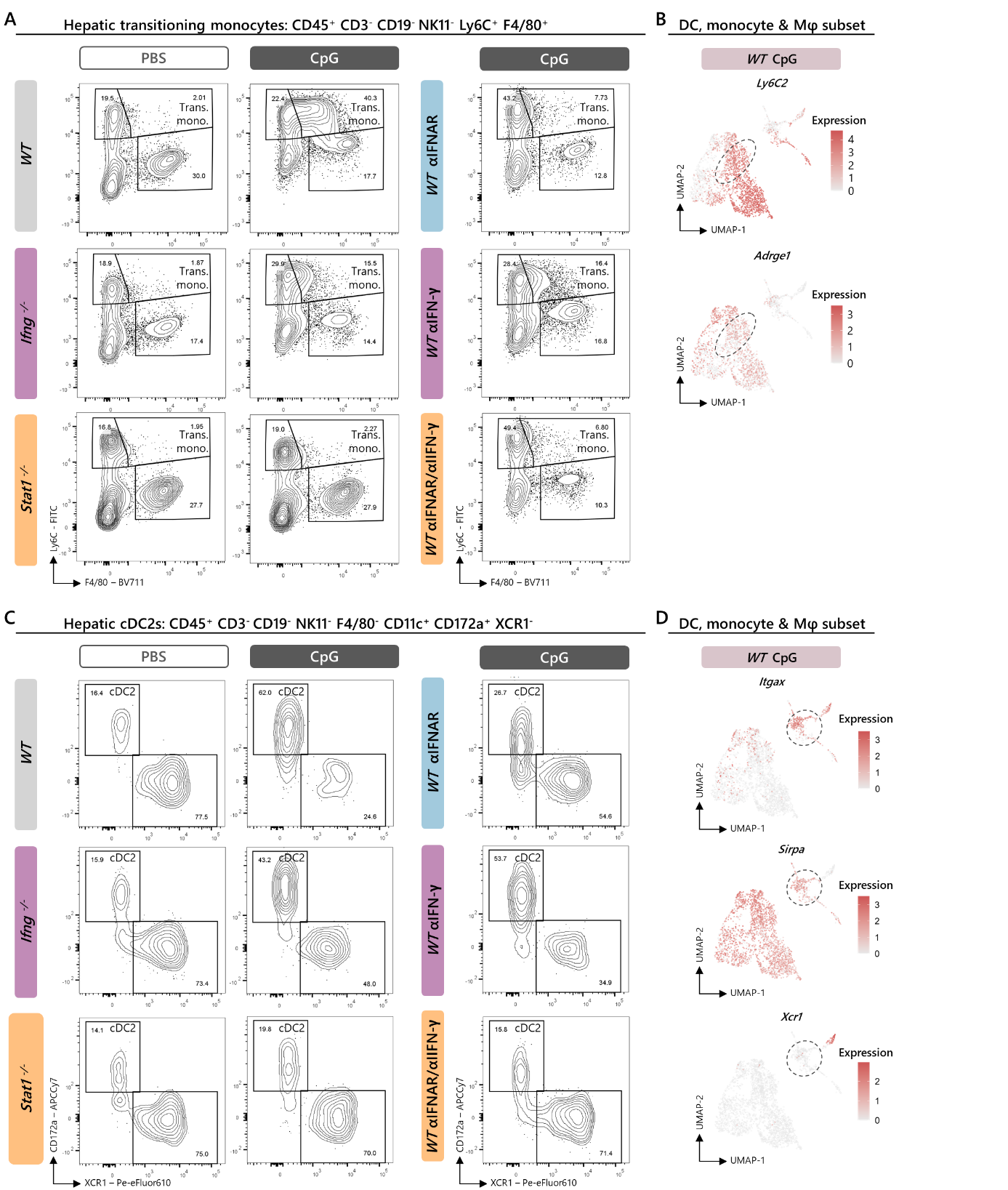
 Suppl. Fig. S8. Comparison of transitioning monocytes and type 2 conventional dendritic cells identified by flow cytometry *versus* CITE-seq.** Experimental setup and gating strategy (**Suppl. Fig. S6**). Representative flow cytometry plots of (A) transitioning monocytes (CD45^+^CD3^-^CD19^-^NK1.1^-^Ly6C^+^F4/80^+^) and, (C) cDC2 (CD45^+^CD3^-^CD19^-^NK1.1^-^Ly6C^-^F4/80^-^CD11c^+^CD172a^+^ XCR1^-^). (B) Lymphoid subclustering and (D) myeloid subclustering of *WT* CpG. Color scale (average log-normalized gene expression).

## **Suppl. Fig. S9**

**
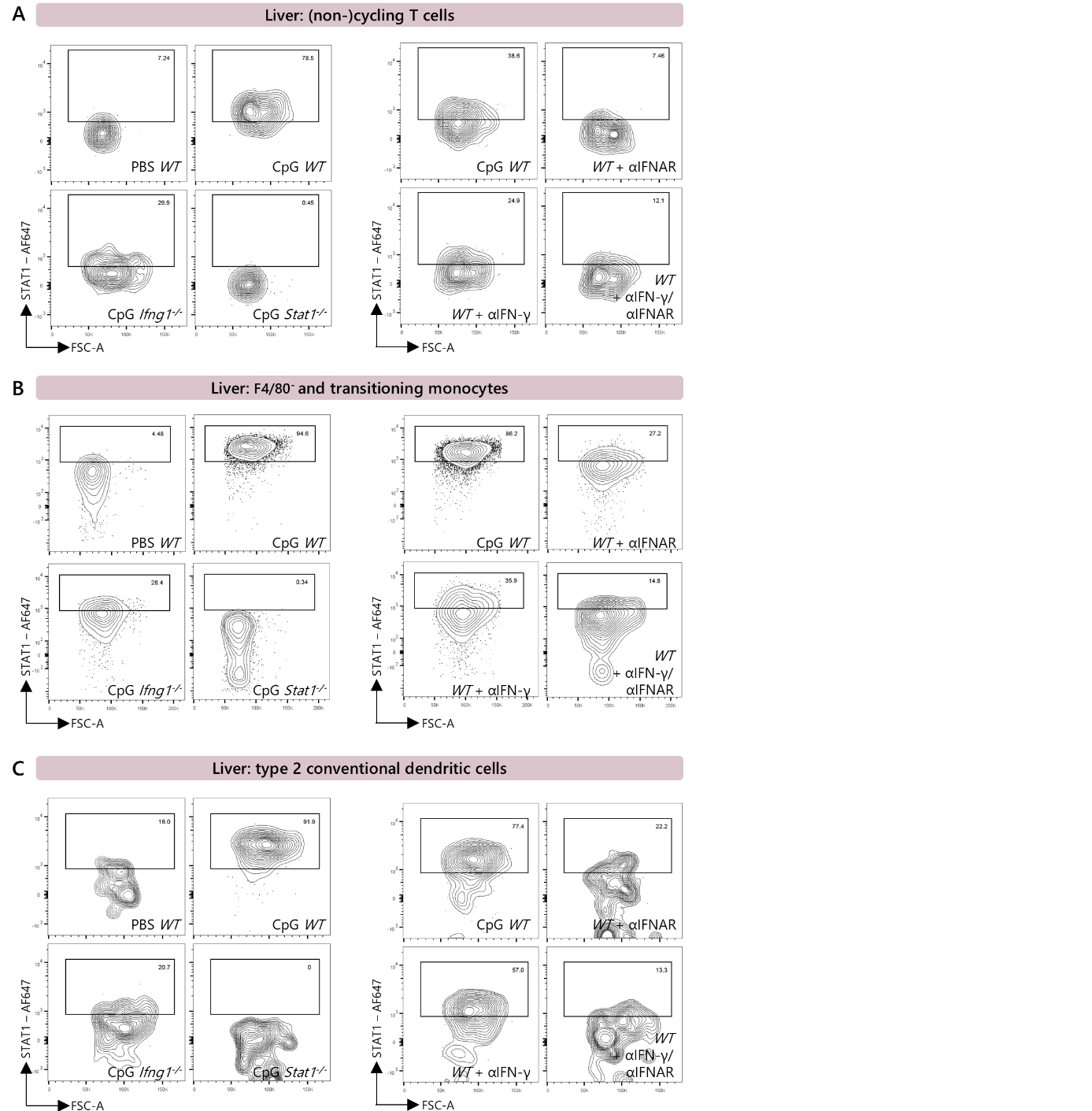
Suppl. Fig. S9. Representative plots of STAT1 expression in T cells, monocytes, and DCs.** Experimental setup and gating strategy (**Suppl. Fig. S6A**). (A) Representative flow cytometry plots of STAT1 expression in non-cycling (*WT* PBS, *Stat1^-/-^* CpG) and cycling T cells (*WT* CpG, *Ifng^-/-^* CpG, *WT* + anti-IFNAR, *WT* + anti-IFN-γ, *WT* + anti-IFNAR and + anti-IFN-γ)*.* (B) Representative flow cytometry plots of STAT1 expression in F4/80^-^ (*WT* PBS, *Stat1^-/-^* CpG, *WT*) and transitioning monocytes (*WT* CpG, *Ifng^-/-^* CpG, *WT* + anti-IFNAR, *WT* + anti-IFN-γ, *WT* + anti-IFNAR and + anti-IFN-γ). (C) Representative flow cytometry plots of STAT1 expression in cDC2.

## **Suppl. Fig. S10**

**
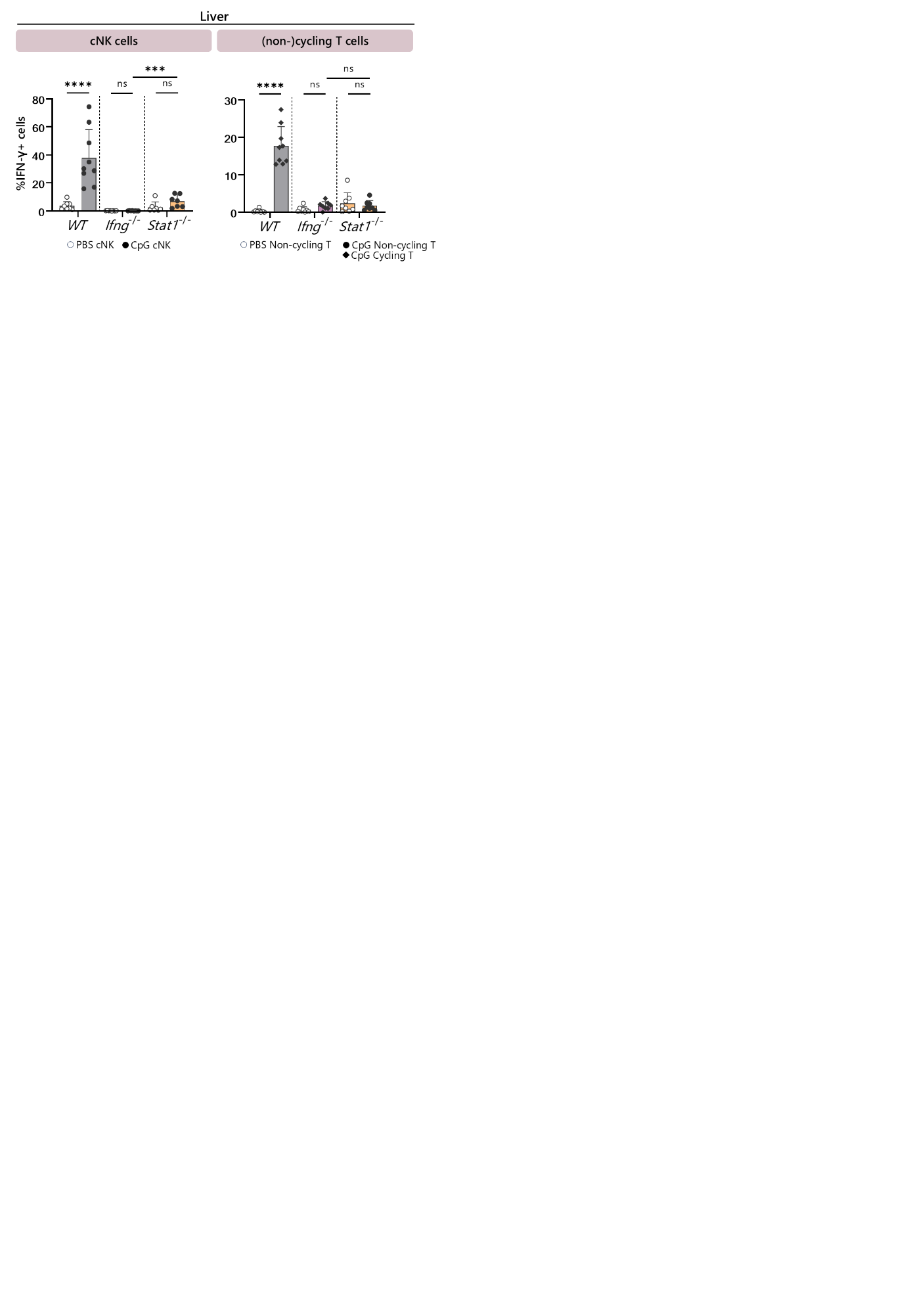
Suppl. Fig. S10. Production of type II IFN by cycling T cells and cNK cells during TLR9-mediated liver inflammation.** Experimental setup and gating strategy (**Suppl. Fig. S6A**). (A) Percentage of IFN-γ^+^ cNK cells, and (non-)cycling T cells. Bars (mean) and error bars (standard deviation). ns, *p*>0.05, ****p*<0.001 *****p*<0.0001 [Student’s t-test (*WT* in cNK cells), Mann-Whitney U-test (*WT* in T cells), Dunn’s multiple-comparisons test (*Ifng^-/-^*, *Stat1^-/-^* in cNK cells, T cells].

Suppl. Fig. S11

**
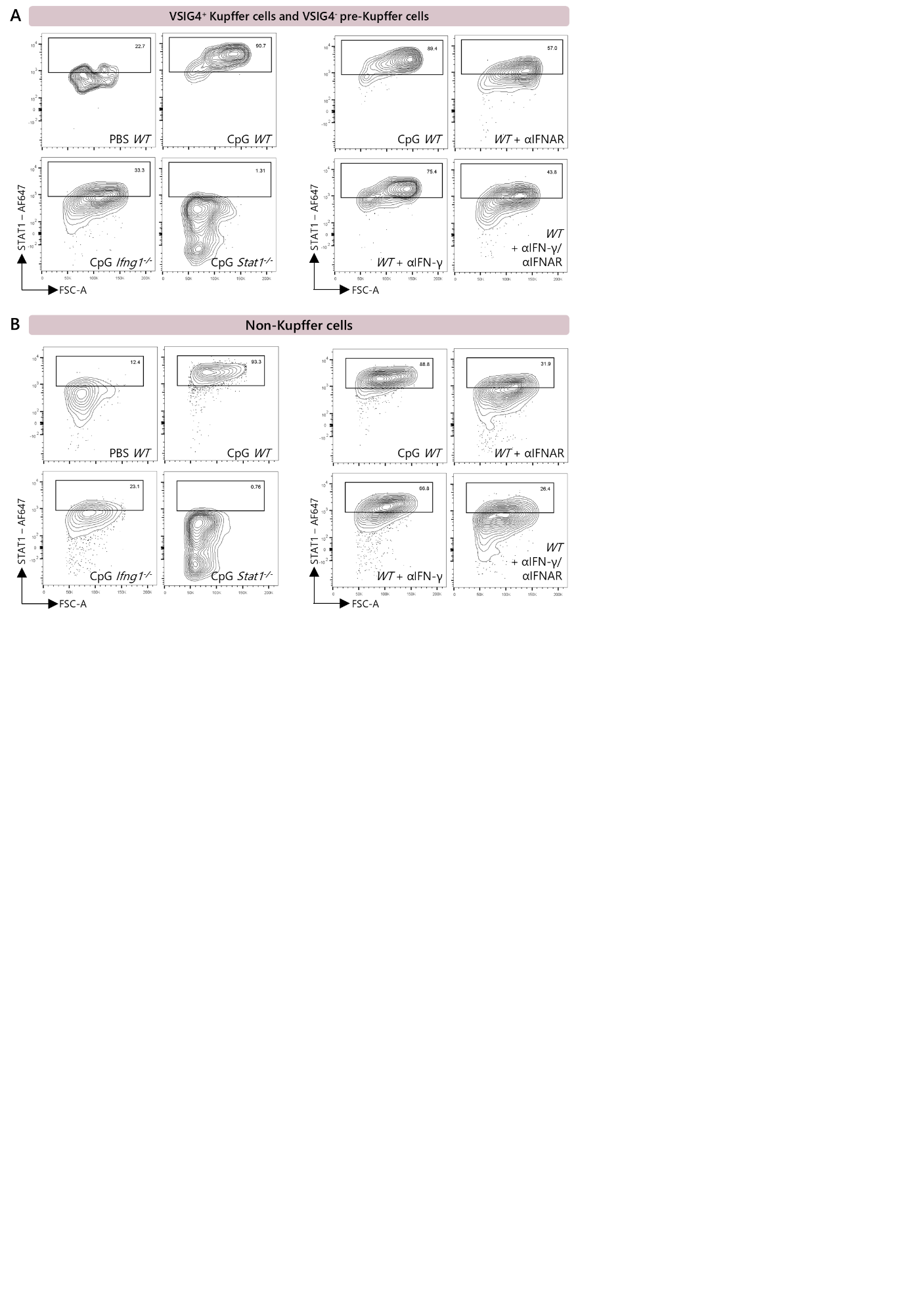
Suppl. Fig. S11. Representative plots of STAT1 expression in macrophage populations.** Experimental setup and gating strategy (**Suppl. Fig. S6A**). (A) Representative plots of STAT1 expression in VSIG4^+^ Kupffer cells (*WT* PBS, *Stat1^-/-^* CpG, *WT* + anti-IFNAR, *WT* + anti-IFN- γ, *WT* + anti-IFNAR and + anti-IFN-γ) and VSIG4^-^ pre-Kupffer cells (*WT* CpG, *Ifng^-/-^* CpG, *Stat1^-/-^* CpG, *WT* + anti-IFNAR, *WT* + anti-IFN-γ, *WT* + anti-IFNAR and + anti-IFN-γ). (B) Representative plots of STAT1 expression in non-Kupffer cells.

## **Suppl. Fig. S12**

**
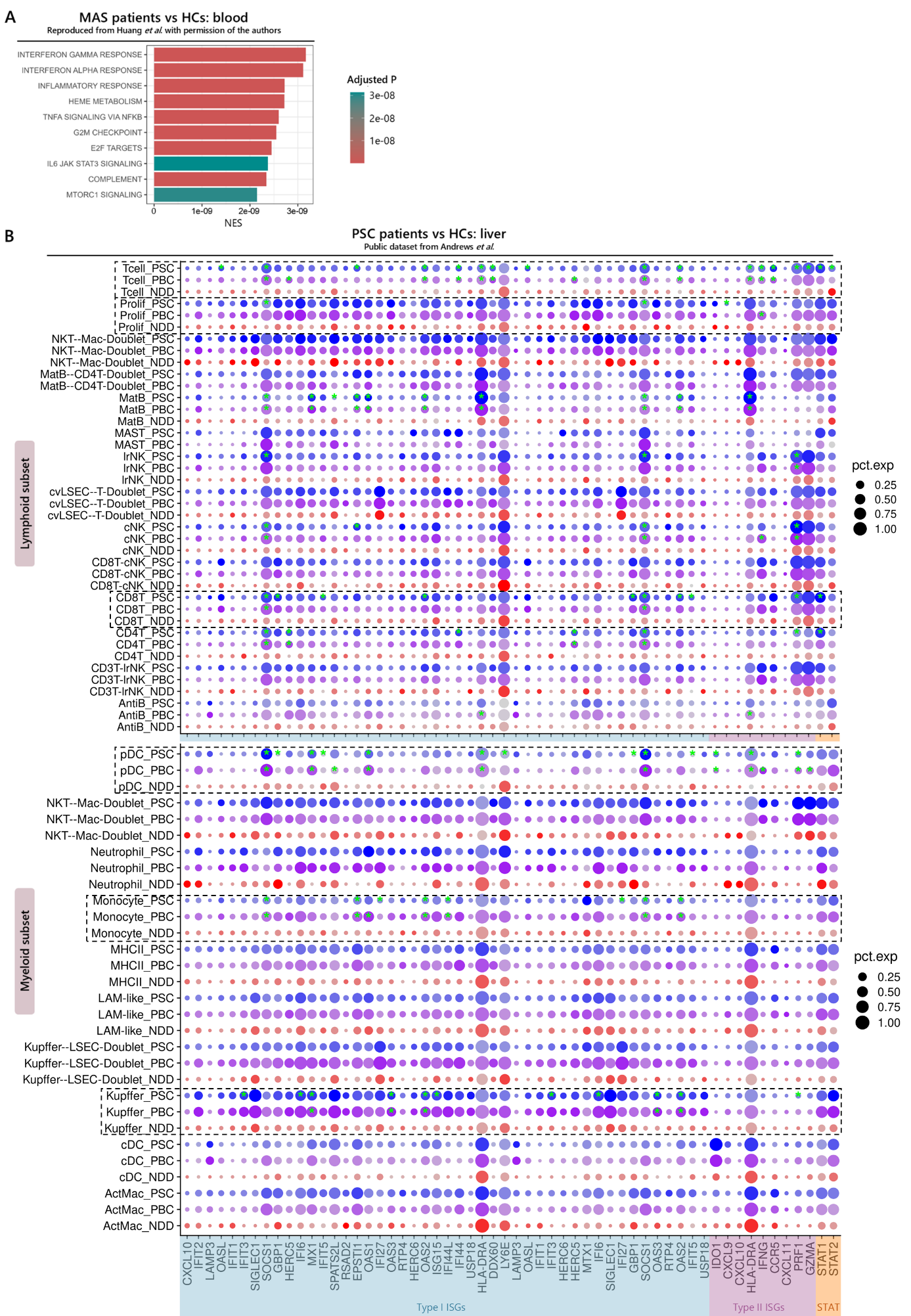
Suppl. Fig. S12. STAT1 expression and a type I and II interferon signature in blood and liver of human TLR9-implicated pathologies**. (A) GSEA of patients with macrophage activation syndrome (MAS) versus healthy controls (HCs) reproduced from the publicly available dataset from Huang *et al*.^1^ (*P_corrected_* <0.05). (B) Expression of type I and II ISGs in patients with primary sclerosing cholangitis (PSC, blue), patients with primary biliary cholangitis (PBC, purple), and HCs (NDD; neurologically deceased healthy donor, red) from the publicly available dataset from Andrews *et al.^3^* Populations discussed in the results are boxed. Stars indicate a significant difference (FDR<5%). Prolif; Proliferating cell, NKT; Natural killer T cell, Mac; Macrophage, MatB; Mature B cell, IrNK; Liver-resident NK cell, cNK; Conventional natural killer cell, antiB; Antibody-secreting B cell, pDC; Plasmacytoid dendritic cell, LAM; Lipid-associated macrophage, (cv)LSEC; (central venous) Sinusoidal endothelial cell, cDC; Conventional dendritic cell, ActMac; Activated macrophage.

##
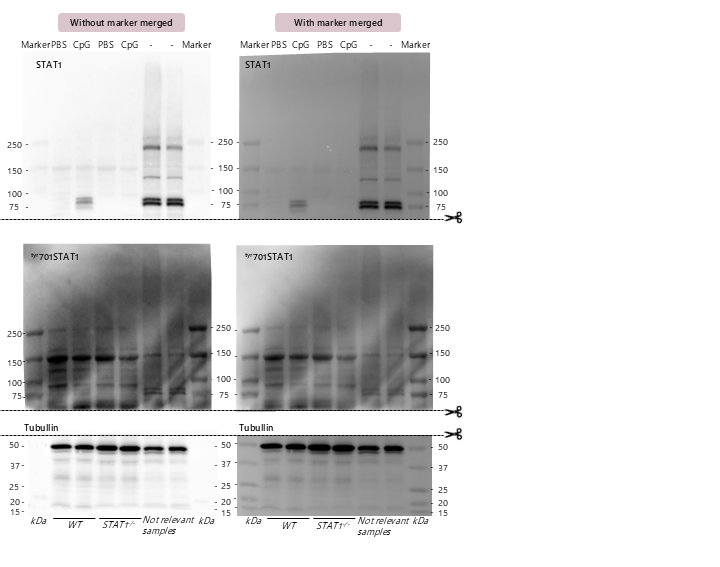
**Suppl. Fig. S13**

**
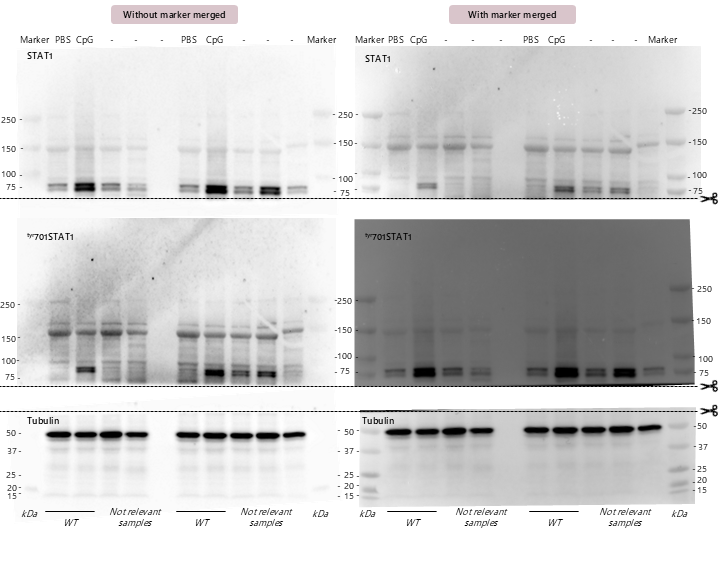

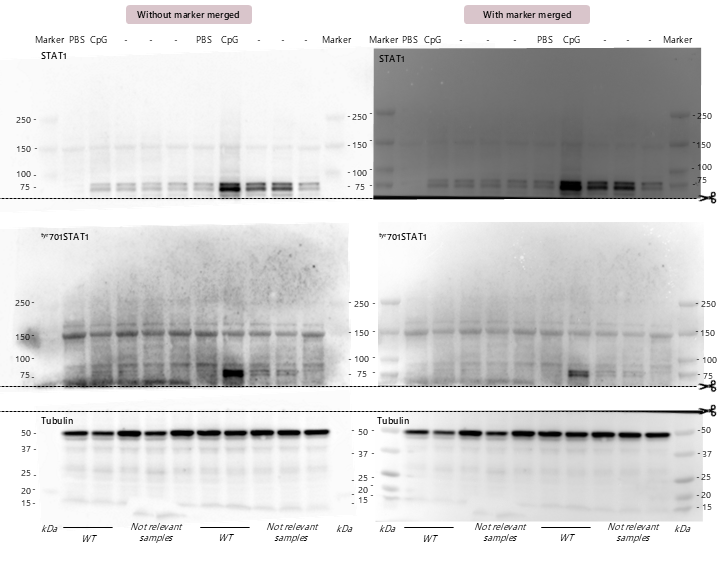
Suppl. Fig. S13 (continuation)**

**
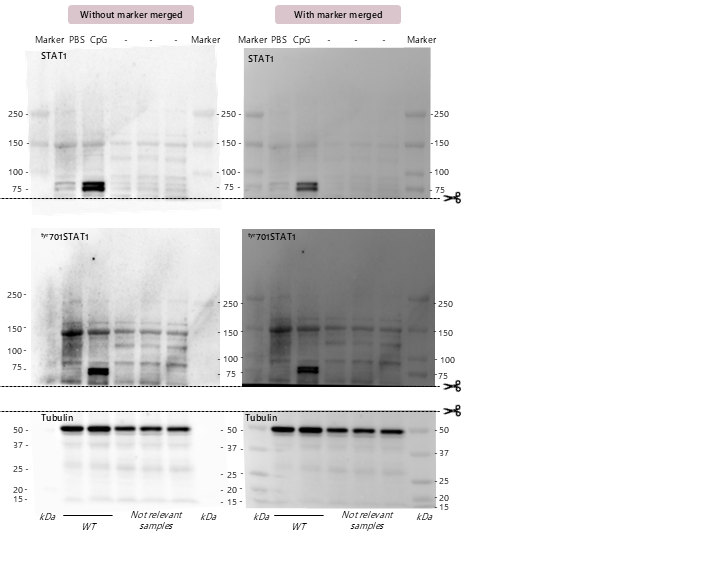
Suppl. Fig. S13 (continuation)**

**
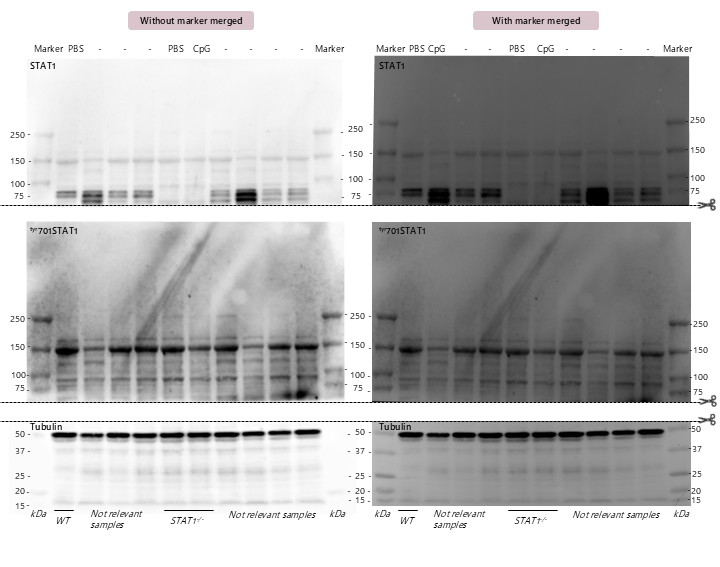

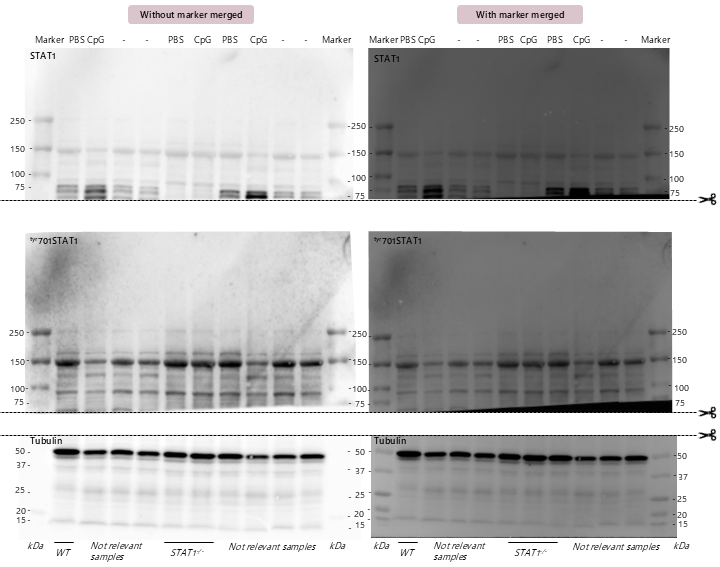
 Suppl. Fig. S13 (continuation)**

**Supplementary Fig. S13. Remaining original western blots of pSTAT1, STAT1, and Tubulin (un)merged with the marker/ladder**. The first western blot is shown in **Suppl. Fig. 2** D). Asterisks refer to lanes not relevant for the data presented in this manuscript. Blots without marker merged were used for formal analysis. Blots were analyzed with ImageJ. Values obtained for STAT and pSTAT were first normalized against Tubulin. Outliers in the pSTAT/STAT ratio were identified by the ROUT method (Q = 1%) and excluded from the analysis. The pSTAT/STAT ratio values of the 8+8 wildtype (WT) mice (PBS and CpG) and the 4+4 STAT1 knockout (STAT1^-/-^) mice (PBS and CpG) are shown in **Fig. 2G**.

# ****Supplementary Tables****

## **Supplementary Table S1. RT-qPCR primers**

| Antigen | Exon location | Assay ID | Fluorescent dye | Company |
| --- | --- | --- | --- | --- |
| *Cxcl9* | Exon 2-3 | Mm.PT.58.57267 | FAM^TM^ | IDT |
| *Cxcl10* | Exon 1-2 | Mm.PT.58.43575827 | FAM^TM^ | IDT |
| *Ifit1* | Exon 1-2 | Mm.PT.58.32674307 | FAM^TM^ | IDT |
| *Ifng* | Exon 1-2 | Mm.PT.58.41769240 | FAM^TM^ | IDT |
| *Il6* | Exon 4-5 | Mm.PT.58.10005566 | FAM^TM^ | IDT |
| *Il12b* | Exon 2-3 | Mm.PT.58.12409997 | FAM^TM^ | IDT |
| *Il18* | Exon 4-5 | Mm.PT.58.42776691 | FAM^TM^ | IDT |
| *Irf7* | Exon 2-3 | Mm.PT.58.32394021.g | FAM^TM^ | IDT |
| *Isg15* | Exon 2-2 | Mm.PT.58.41476392.g | FAM^TM^ | IDT |
| *Siglec1* | Exon 17-18 | Mm.PT.58.8446763 | FAM^TM^ | IDT |
| *Tnfa* | Exon 2-4 | Mm.PT.58.12575861 | FAM^TM^ | IDT |

## **Supplementary Table S2. Fluorochrome-labelled antibodies**

| Antigen | Clone | Fluorochrome | Company |
| --- | --- | --- | --- |
| FACS of CD45+ with enriched F4/80+ cells for CITE-seq | | | |
| CD45 | 30-F11 | PerCP-Cy5.5 | BioLegend |
| CD49a | Ha31/8 | PE | BD Biosciences |
| CD3 | 145-2C11 | BV786 | BD Biosciences |
| CD19 | 6D5 | BV785 | BioLegend |
| F4/80 | Bm8 | BV711 | BioLegend |
| Flow cytometric validation of TLR9-associated populations | | | |
| CD3 | 145-2C11 | PerCP-Cy5.5, PE-Cy5, PE-Cy7 | Invitrogen, BioLegend |
| CD8 | 53-6.7 | PE-Cy5, FITC, BV786 | Invitrogen, BioLegend |
| CD11c | N418 | BV711, PerCP-Cy5.5 | BioLegend |
| CD19 | 6D5 | PE-Cy5 | BioLegend |
| CD38 | 90/CD38 | BV711 | BD Biosciences |
| CD45 | 30-F11 | BUV395 | Invitrogen |
| CD172a | P84 | APC-Cy7 | BioLegend |
| CD317 | q27 | BV605 | BioLegend |
| CLEC2 | 17D9/CLEC-2 | PE | BioLegend |
| F4/80 | BM8 | PE, PE-Cy5 | BioLegend |
| IFN-γ | XMG1.2 | PE | BioLegend |
| Ki67 | B56 | BUV395 | BD Biosciences |
| Ly6C | HK1.4 | FITC | BioLegend |
| NK1.1 | PK136 | PE-Cy5, APC-eF780 | BioLegend, Invitrogen |
| STAT1 | 1/STAT1 | AF647 | BD Biosciences |
| VSIG4 | NLA14 | PE-Dazzle594 | Invitrogen |
| XCR1 | ZET | PE-Dazzle594 | BioLegend |

## **Supplementary Table S3. Oligo-labelled antibodies**

| Antigen | Clone | Identifier | Company |
| --- | --- | --- | --- |
| Allophycocyanin | APC003 | TotalSeq-C0987 | BioLegend |
| Biotin | 1D4-C5 | TotalSeq-C0436 | BioLegend |
| CD1d | 1B1 | TotalSeq-C0851 | BioLegend |
| CD2 | RM2-5 | TotalSeq-C0892 | BioLegend |
| CD3 | 17A2 | TotalSeq-C0182 | BioLegend |
| CD5 | 53-7.3 | TotalSeq-C0111 | BioLegend |
| CD8a | 53-6.7 | TotalSeq-C0002 | BioLegend |
| CD8b | YTS156.7.7 | TotalSeq-C0230 | BioLegend |
| CD9 | MZ3 | TotalSeq-C0813 | BioLegend |
| CD11a | M17/4 | TotalSeq-C0595 | BioLegend |
| CD11b | M1/70 | TotalSeq-C0014 | BioLegend |
| CD11c | N418 | TotalSeq-C0106 | BioLegend |
| CD14 | Sa14-2 | TotalSeq-C0424 | BioLegend |
| CD15 | MC-480 | TotalSeq-C0076 | BioLegend |
| CD16/32 | 93 | TotalSeq-C0109 | BioLegend |
| CD19 | 6D5 | TotalSeq-C0093 | BioLegend |
| CD20 | SA275A11 | TotalSeq-C0192 | BioLegend |
| CD21,CD35 | 7,00E+09 | TotalSeq-C0107 | BioLegend |
| CD22 | OX-97 | TotalSeq-C0827 | BioLegend |
| CD23 | B3B4 | TotalSeq-C0108 | BioLegend |
| CD24 | M1/69 | TotalSeq-C0212 | BioLegend |
| CD25 | PC61 | TotalSeq-C0097 | BioLegend |
| CD27 | LG.3A10 | TotalSeq-C0191 | BioLegend |
| CD28 | 37.51 | TotalSeq-C0204 | BioLegend |
| CD29 | HMβ1-1 | TotalSeq-C0570 | BioLegend |
| CD30 | mCD30.1 | TotalSeq-C1062 | BioLegend |
| CD31 | 390 | TotalSeq-C0904 | BioLegend |
| CD34 | SA376A4 | TotalSeq-C0823 | BioLegend |
| CD34 | HM34 | TotalSeq-C0857 | BioLegend |
| CD38 | 90 | TotalSeq-C0557 | BioLegend |
| CD39 | Duha59 | TotalSeq-C0834 | BioLegend |
| CD40 | mrt/23 | TotalSeq-C0903 | BioLegend |
| CD41 | MWReg30 | TotalSeq-C0443 | BioLegend |
| CD43 | S11 | TotalSeq-C0110 | BioLegend |
| CD44 | IM7 | TotalSeq-C0073 | BioLegend |
| CD45 | 30-F11 | TotalSeq-C0096 | BioLegend |
| CD45.1 | A20 | TotalSeq-C0178 | BioLegend |
| CD45.2 | 104 | TotalSeq-C0157 | BioLegend |
| CD45R/B220 | RA3-6B2 | TotalSeq-C0103 | BioLegend |
| CD45RB | C363-16A | TotalSeq-C1063 | BioLegend |
| CD48 | HM48-1 | TotalSeq-C0429 | BioLegend |
| CD49a | HMα1 | TotalSeq-C0850 | BioLegend |
| CD49b | HMα2 | TotalSeq-C0421 | BioLegend |
| CD49d | R1-2 | TotalSeq-C0078 | BioLegend |
| CD49f | GoH3 | TotalSeq-C0070 | BioLegend |
| CD54 | YN1/1.7.4 | TotalSeq-C0074 | BioLegend |
| CD55 | RIKO-3 | TotalSeq-C0558 | BioLegend |
| CD61 | 2C9.G2 (HMβ3-1) | TotalSeq-C0910 | BioLegend |
| CD62L | MEL-14 | TotalSeq-C0112 | BioLegend |
| CD62P | RMP-1 | TotalSeq-C0229 | BioLegend |
| CD63 | NVG-2 | TotalSeq-C0559 | BioLegend |
| CD64 | X54-5/7.1 | TotalSeq-C0202 | BioLegend |
| CD68 | FA-11 | TotalSeq-C0560 | BioLegend |
| CD69 | H1.2F3 | TotalSeq-C0197 | BioLegend |
| CD71 | RI7217 | TotalSeq-C0441 | BioLegend |
| CD73 | TY/11.8 | TotalSeq-C0077 | BioLegend |
| CD79b | HM79-12 | TotalSeq-C0561 | BioLegend |
| CD80 | 16-10A1 | TotalSeq-C0849 | BioLegend |
| CD81 | Eat-2 | TotalSeq-C1064 | BioLegend |
| CD83 | Michel-19 | TotalSeq-C0562 | BioLegend |
| CD85k | H1.1 | TotalSeq-C1007 | BioLegend |
| CD86 | GL-1 | TotalSeq-C0200 | BioLegend |
| CD88 | 20/70 | TotalSeq-C1042 | BioLegend |
| CD90.2 | 30-H12 | TotalSeq-C0075 | BioLegend |
| CD90/CD90.1 | OX-7 | TotalSeq-C0380 | BioLegend |
| CD93 | AA4.1 | TotalSeq-C0113 | BioLegend |
| CD94 | 18d3 | TotalSeq-C1009 | BioLegend |
| CD95 | SA367H8 | TotalSeq-C0917 | BioLegend |
| CD96 | 3.3 | TotalSeq-C0906 | BioLegend |
| CD98 | RL388 | TotalSeq-C0989 | BioLegend |
| CD103 | 2E7 | TotalSeq-C0201 | BioLegend |
| CD104 | 346-11A | TotalSeq-C1028 | BioLegend |
| CD105 | MJ7/18 | TotalSeq-C0812 | BioLegend |
| CD106 | 429 (MVCAM.A) | TotalSeq-C0226 | BioLegend |
| CD115 | AFS98 | TotalSeq-C0105 | BioLegend |
| CD117 | 2B8 | TotalSeq-C0012 | BioLegend |
| CD120b | TR75-89 | TotalSeq-C0893 | BioLegend |
| CD122 | 5H4 | TotalSeq-C0227 | BioLegend |
| CD124 | I015F8 | TotalSeq-C0916 | BioLegend |
| CD127 | A7R34 | TotalSeq-C0198 | BioLegend |
| CD134 | OX-86 | TotalSeq-C0195 | BioLegend |
| CD135 | A2F10 | TotalSeq-C0098 | BioLegend |
| CD137 | 17B5 | TotalSeq-C0194 | BioLegend |
| CD138 | 281-2 | TotalSeq-C0810 | BioLegend |
| CD140a | APA5 | TotalSeq-C0573 | BioLegend |
| CD146 | P1H12 | TotalSeq-C0134 | BioLegend |
| CD150 | TC15-12F12.2 | TotalSeq-C0203 | BioLegend |
| CD152 | UC10-4B9 | TotalSeq-C0388 | BioLegend |
| CD155 | TX56 | TotalSeq-C1011 | BioLegend |
| CD160 | 7H1 | TotalSeq-C1006 | BioLegend |
| CD163 | S15049I | TotalSeq-C0417 | BioLegend |
| CD169 | 3D6.112 | TotalSeq-C0440 | BioLegend |
| CD170 | S17007L | TotalSeq-C0431 | BioLegend |
| CD172a | P84 | TotalSeq-C0422 | BioLegend |
| CD178 | MFL3 | TotalSeq-C1012 | BioLegend |
| CD182 | SA044G4 | TotalSeq-C0909 | BioLegend |
| CD183 | CXCR3-173 | TotalSeq-C0228 | BioLegend |
| CD185 | L138D7 | TotalSeq-C0846 | BioLegend |
| CD186 | SA051D1 | TotalSeq-C0926 | BioLegend |
| CD192 | SA203G11 | TotalSeq-C0426 | BioLegend |
| CD193 | J073E5 | TotalSeq-C0808 | BioLegend |
| CD194 | 2G12 | TotalSeq-C0833 | BioLegend |
| CD196 | 29-2L17 | TotalSeq-C0225 | BioLegend |
| CD197 | 4B12 | TotalSeq-C0377 | BioLegend |
| CD198 | SA214G2 | TotalSeq-C0907 | BioLegend |
| CD200 | OX-90 | TotalSeq-C0079 | BioLegend |
| CD200R | OX-110 | TotalSeq-C0807 | BioLegend |
| CD201 | RCR-16 | TotalSeq-C0439 | BioLegend |
| CD204 | 1F8C33 | TotalSeq-C0448 | BioLegend |
| CD205 | NLDC-145 | TotalSeq-C1010 | BioLegend |
| CD207 | 4C7 | TotalSeq-C0437 | BioLegend |
| CD210 | 1B1.3a | TotalSeq-C1032 | BioLegend |
| CD223 | C9B7W | TotalSeq-C0378 | BioLegend |
| CD226 | 1,00E+06 | TotalSeq-C0852 | BioLegend |
| CD226 | TX42.1 | TotalSeq-C0949 | BioLegend |
| CD252 | RM134L | TotalSeq-C0924 | BioLegend |
| CD270 | HMHV-1B18 | TotalSeq-C0885 | BioLegend |
| CD272 | 6A6 | TotalSeq-C0881 | BioLegend |
| CD273 | TY25 | TotalSeq-C0914 | BioLegend |
| CD274 | MIH6 | TotalSeq-C0190 | BioLegend |
| CD278 | C398.4A | TotalSeq-C0171 | BioLegend |
| CD278 | 7E.17G9 | TotalSeq-C0847 | BioLegend |
| CD279 | RMP1-30 | TotalSeq-C0004 | BioLegend |
| CD300LG | ZAQ5 | TotalSeq-C0416 | BioLegend |
| CD301b | URA-1 | TotalSeq-C0566 | BioLegend |
| CD304 | 3,00E+12 | TotalSeq-C0552 | BioLegend |
| CD309 | Avas12 | TotalSeq-C0553 | BioLegend |
| CD314 | CX5 | TotalSeq-C0835 | BioLegend |
| CD317 | 927 | TotalSeq-C0811 | BioLegend |
| CD326 | G8.8 | TotalSeq-C0449 | BioLegend |
| CD335 | 29A1.4 | TotalSeq-C0184 | BioLegend |
| CD357 | DTA-1 | TotalSeq-C0193 | BioLegend |
| CD366 | RMT3-23 | TotalSeq-C0003 | BioLegend |
| CD370 | 7H11 | TotalSeq-C0556 | BioLegend |
| CD371 | 5D3/CLEC12A | TotalSeq-C0825 | BioLegend |
| CX3CR1 | SA011F11 | TotalSeq-C0563 | BioLegend |
| DLL1 | HMD1-3 | TotalSeq-C0884 | BioLegend |
| DR3 | 4C12 | TotalSeq-C0836 | BioLegend |
| ESAM | 1G8/ESAM | TotalSeq-C0596 | BioLegend |
| F4/80 | BM8 | TotalSeq-C0114 | BioLegend |
| FcεRIα | MAR-1 | TotalSeq-C0115 | BioLegend |
| FITC (FITC labeled anti-CD49a - Ha31/8) | FIT-22 | TotalSeq-C0988 | BioLegend |
| FR4 | TH6 | TotalSeq-C1058 | BioLegend |
| GPR56 | CG4 | TotalSeq-C0912 | BioLegend |
| H-2Kb | 25-D1.16 | TotalSeq-C0231 | BioLegend |
| HA.11 | 16B12 | TotalSeq-C1131 | BioLegend |
| I-A/I-E | M5/114.15.2 | TotalSeq-C0117 | BioLegend |
| IgD | 11-26c.2a | TotalSeq-C0571 | BioLegend |
| IgG Isotype Ctrl | HTK888 | TotalSeq-C0241 | BioLegend |
| IgG1 | RMG1-1 | TotalSeq-C1167 | BioLegend |
| IgG1, κ | MOPC-21 | TotalSeq-C0090 | BioLegend |
| IgG1, κ Isotype Ctrl | RTK2071 | TotalSeq-C0236 | BioLegend |
| IgG1, λ Isotype Ctrl | G0114F7 | TotalSeq-C0237 | BioLegend |
| IgG2a, κ | MOPC-173 | TotalSeq-C0091 | BioLegend |
| IgG2a, κ Isotype Ctrl | RTK2758 | TotalSeq-C0238 | BioLegend |
| IgG2b | RMG2b-1 | TotalSeq-C1168 | BioLegend |
| IgG2b κ | RTK4530 | TotalSeq-C0095 | BioLegend |
| IgG2b, κ Isotype Ctrl | MPC-11 | TotalSeq-C0092 | BioLegend |
| IgM | RMM-1 | TotalSeq-C0450 | BioLegend |
| IL-21R | 4A9 | TotalSeq-C0879 | BioLegend |
| IL-33Rα | DIH9 | TotalSeq-C0837 | BioLegend |
| Integrin β7 | FIB504 | TotalSeq-C0214 | BioLegend |
| JAML | 4,00E+10 | TotalSeq-C0877 | BioLegend |
| KLRG1 | 2F1/KLRG1 | TotalSeq-C0250 | BioLegend |
| Ly108 | 330-AJ | TotalSeq-C0930 | BioLegend |
| Ly49A | YE1/48.10.6 | TotalSeq-C0842 | BioLegend |
| Ly49D | 4,00E+05 | TotalSeq-C0841 | BioLegend |
| Ly49G | AT8 | TotalSeq-C0840 | BioLegend |
| Ly49H | 3D10 | TotalSeq-C0839 | BioLegend |
| Ly-51 | 6C3 | TotalSeq-C1034 | BioLegend |
| Ly-6A/E | D7 | TotalSeq-C0130 | BioLegend |
| Ly6C | HK1.4 | TotalSeq-C0013 | BioLegend |
| Ly6G | 1A8 | TotalSeq-C0015 | BioLegend |
| Ly-6G/Ly-6C | RB6-8C5 | TotalSeq-C0116 | BioLegend |
| MAdCAM-1 | MECA-367 | TotalSeq-C0232 | BioLegend |
| MERTK | 2B10C42 | TotalSeq-C0565 | BioLegend |
| NK-1.1 | PK136 | TotalSeq-C0118 | BioLegend |
| Notch 1 | HMN1-12 | TotalSeq-C0442 | BioLegend |
| Notch 4 | HMN4-14 | TotalSeq-C0888 | BioLegend |
| P2X7R | 1F11 | TotalSeq-C0824 | BioLegend |
| Rat IgG2c, κ | RTK4174 | TotalSeq-C0240 | BioLegend |
| Siglec H | 551 | TotalSeq-C0119 | BioLegend |
| TCR Va2 | B20.1 | TotalSeq-C0981 | BioLegend |
| TCR Vα 8.3 | B21.14 | TotalSeq-C0982 | BioLegend |
| TCR Vα 8.3 | KT50 | TotalSeq-C0983 | BioLegend |
| TCR Vα11 | RR8-1 | TotalSeq-C0984 | BioLegend |
| TCR Vβ5.1, 5.2 | MR9-4 | TotalSeq-C0354 | BioLegend |
| TCR Vβ8.1,8.2 | KJ16-133.18 | TotalSeq-C0235 | BioLegend |
| TCR Vγ1.1 | 2.11 | TotalSeq-C0209 | BioLegend |
| TCR Vγ2 | UC3-10A6 | TotalSeq-C0211 | BioLegend |
| TCR Vγ3 | 536 | TotalSeq-C0210 | BioLegend |
| TCR β chain | H57-597 | TotalSeq-C0120 | BioLegend |
| TCR γ/δ | GL3 | TotalSeq-C0121 | BioLegend |
| TCR γ/δ | UC7-13D5 | TotalSeq-C0986 | BioLegend |
| TER-119 | TER-119 | TotalSeq-C0122 | BioLegend |
| TIGIT | 1G9 | TotalSeq-C0848 | BioLegend |
| Tim-4 | RMT4-54 | TotalSeq-C0567 | BioLegend |
| VISTA | MIH63 | TotalSeq-C0915 | BioLegend |
| XCR1 | ZET | TotalSeq-C0568 | BioLegend |

# ****References****

1. **Huang Z**, **Brodeur KE**, **Chen L**, et al. Type I interferon signature and cycling lymphocytes in macrophage activation syndrome. J Clin Invest 2023;133(22):e165616.

2. **Andrews TS**, **Nakib D**, Perciani CT, et al. Single-cell, single-nucleus, and spatial transcriptomics characterization of the immunological landscape in the healthy and PSC human liver. J Hepatol 2024;80(5):730–43.

3. **Kim H**, **De Jesus AA**, **Brooks SR**, et al. Development of a Validated Interferon Score Using NanoString Technology. Journal of Interferon and Cytokine Research 2018;38(4):171–85

4. **Cui C**, **Xu C**, Yang W, et al. Ratio of the interferon-γ signature to the immunosuppression signature predicts anti-PD-1 therapy response in melanoma. NPJ Genom Med 2021;6(1).
