# Supplementary material for: STAT1 drives the immune landscape of murine Toll-like receptor 9-induced liver inflammation
Source: JHEP Rep. 2025 Nov 6;8(2):101668. doi: 10.1016/j.jhepr.2025.101668 (PMC12814853; doi:10.1016/j.jhepr.2025.101668)
Supplement: Multimedia component 2 [file mmc2.docx]

**JHEP Reports**

**CTAT methods**

Tables for a “Complete, Transparent, Accurate and Timely account” (CTAT) are now mandatory for all revised submissions. The aim is to enhance the reproducibility of methods.

- Only include the parts relevant to your study
- Refer to the CTAT in the main text as ‘Supplementary CTAT Table’
- Do not add subheadings
- Add as many rows as needed to include all information
- Only include one item per row

**If the CTAT form is not relevant to your study, please outline the reasons why:**

| / |
| --- |

- 1. **Antibodies**

| **Name** | | **Citation** | **Supplier** | **Cat no.** | **Clone no.** |
| --- | --- | --- | --- | --- | --- |
| **Antigen** | **Fluorochrome** |  |  |  |  |
| CD45 | PerCP-Cy5.5 | n/a | BioLegend | 103132 | 30-F11 |
| CD49a | PE | n/a | BD Biosciences | 562115 | Ha31/8 |
| CD3 | BV786 | n/a | BD Biosciences | 564379 | 145-2C11 |
| CD19 | BV785 | n/a | BIoLegend | 115543 | 6D5 |
| F4/80 | BV711 | n/a | BIoLegend | 123147 | Bm8 |
| CD3 | PerCP-Cy5.5,  PE-Cy5,  PE-Cy7 | n/a | Invitrogen, BioLegend | 11-0452-82,  15-0031-82,  25-0031-82 | 145-2C11 |
| CD8 | PE-Cy5, FITC, BV785 | n/a | Invitrogen, BioLegend | 100710, 100706,100750 | 53-6.7 |
| CD11c | BV711,  PerCP-Cy5.5 | n/a | BioLegend | 117349,1173 | N418 |
| CD19 | PE-Cy5 | n/a | BioLegend | 115510 | 6D5 |
| CD38 | BV711 | n/a | BD Biosciences | 740697 | 90/CD38 |
| CD45 | BUV395 | n/a | Invitrogen | 363-0451-82 | 30-F11 |
| CD172a | APC-Cy7 | n/a | BioLegend | 144018 | P84 |
| CD317 | BV605 | n/a | BioLegend | 127025 | 927 |
| CLEC2 | PE | n/a | BioLegend | 146104 | 17D9/CLEC-2 |
| F4/80 | PE, PE-Cy5 | n/a | BioLegend | 123110, 123114 | BM8 |
| IFN-γ | PE | n/a | BioLegend | 505808 | XMG1.2 |
| Ki67 | BUV395 | n/a | BD Biosciences | 564071 | B56 |
| Ly6C | FITC | n/a | BioLegend | 128006 | HK1.4 |
| NK1.1 | PE-Cy5,  APC-eF780 | n/a | BioLegend, Invitrogen | 108716,  47-5941-82 | PK136 |
| STAT1 | AF647 | n/a | BD Biosciences | 558560 | 1/STAT1 |
| VSIG4 | PE-Dazzle594 | n/a | Invitrogen | 17-5752-82 | NLA14 |
| XCR1 | PE-Dazzle594 | n/a | BioLegend | 148234 | ZET |
| Allophycocyanin |  | n/a | BioLegend | TotalSeq-C0987 | APC003 |
| Biotin |  | n/a | BioLegend | TotalSeq-C0436 | 1D4-C5 |
| CD1d |  | n/a | BioLegend | TotalSeq-C0851 | 1B1 |
| CD2 |  | n/a | BioLegend | TotalSeq-C0892 | RM2-5 |
| CD3 |  | n/a | BioLegend | TotalSeq-C0182 | 17A2 |
| CD5 |  | n/a | BioLegend | TotalSeq-C0111 | 53-7.3 |
| CD8a |  | n/a | BioLegend | TotalSeq-C0002 | 53-6.7 |
| CD8b |  | n/a | BioLegend | TotalSeq-C0230 | YTS156.7.7 |
| CD9 |  | n/a | BioLegend | TotalSeq-C0813 | MZ3 |
| CD11a |  | n/a | BioLegend | TotalSeq-C0595 | M17/4 |
| CD11b |  | n/a | BioLegend | TotalSeq-C0014 | M1/70 |
| CD11c |  | n/a | BioLegend | TotalSeq-C0106 | N418 |
| CD14 |  | n/a | BioLegend | TotalSeq-C0424 | Sa14-2 |
| CD15 |  | n/a | BioLegend | TotalSeq-C0076 | MC-480 |
| CD16/32 |  | n/a | BioLegend | TotalSeq-C0109 | 93 |
| CD19 |  | n/a | BioLegend | TotalSeq-C0093 | 6D5 |
| CD20 |  | n/a | BioLegend | TotalSeq-C0192 | SA275A11 |
| CD21,CD35 |  | n/a | BioLegend | TotalSeq-C0107 | 7,00E+09 |
| CD22 |  | n/a | BioLegend | TotalSeq-C0827 | OX-97 |
| CD23 |  | n/a | BioLegend | TotalSeq-C0108 | B3B4 |
| CD24 |  | n/a | BioLegend | TotalSeq-C0212 | M1/69 |
| CD25 |  | n/a | BioLegend | TotalSeq-C0097 | PC61 |
| CD27 |  | n/a | BioLegend | TotalSeq-C0191 | LG.3A10 |
| CD28 |  | n/a | BioLegend | TotalSeq-C0204 | 37.51 |
| CD29 |  | n/a | BioLegend | TotalSeq-C0570 | HMβ1-1 |
| CD30 |  | n/a | BioLegend | TotalSeq-C1062 | mCD30.1 |
| CD31 |  | n/a | BioLegend | TotalSeq-C0904 | 390 |
| CD34 |  | n/a | BioLegend | TotalSeq-C0823 | SA376A4 |
| CD34 |  | n/a | BioLegend | TotalSeq-C0857 | HM34 |
| CD38 |  | n/a | BioLegend | TotalSeq-C0557 | 90 |
| CD39 |  | n/a | BioLegend | TotalSeq-C0834 | Duha59 |
| CD40 |  | n/a | BioLegend | TotalSeq-C0903 | mrt/23 |
| CD41 |  | n/a | BioLegend | TotalSeq-C0443 | MWReg30 |
| CD43 |  | n/a | BioLegend | TotalSeq-C0110 | S11 |
| CD44 |  | n/a | BioLegend | TotalSeq-C0073 | IM7 |
| CD45 |  | n/a | BioLegend | TotalSeq-C0096 | 30-F11 |
| CD45.1 |  | n/a | BioLegend | TotalSeq-C0178 | A20 |
| CD45.2 |  | n/a | BioLegend | TotalSeq-C0157 | 104 |
| CD45R/B220 |  | n/a | BioLegend | TotalSeq-C0103 | RA3-6B2 |
| CD45RB |  | n/a | BioLegend | TotalSeq-C1063 | C363-16A |
| CD48 |  | n/a | BioLegend | TotalSeq-C0429 | HM48-1 |
| CD49a |  | n/a | BioLegend | TotalSeq-C0850 | HMα1 |
| CD49b |  | n/a | BioLegend | TotalSeq-C0421 | HMα2 |
| CD49d |  | n/a | BioLegend | TotalSeq-C0078 | R1-2 |
| CD49f |  | n/a | BioLegend | TotalSeq-C0070 | GoH3 |
| CD54 |  | n/a | BioLegend | TotalSeq-C0074 | YN1/1.7.4 |
| CD55 |  | n/a | BioLegend | TotalSeq-C0558 | RIKO-3 |
| CD61 |  | n/a | BioLegend | TotalSeq-C0910 | 2C9.G2 (HMβ3-1) |
| CD62L |  | n/a | BioLegend | TotalSeq-C0112 | MEL-14 |
| CD62P |  | n/a | BioLegend | TotalSeq-C0229 | RMP-1 |
| CD63 |  | n/a | BioLegend | TotalSeq-C0559 | NVG-2 |
| CD64 |  | n/a | BioLegend | TotalSeq-C0202 | X54-5/7.1 |
| CD68 |  | n/a | BioLegend | TotalSeq-C0560 | FA-11 |
| CD69 |  | n/a | BioLegend | TotalSeq-C0197 | H1.2F3 |
| CD71 |  | n/a | BioLegend | TotalSeq-C0441 | RI7217 |
| CD73 |  | n/a | BioLegend | TotalSeq-C0077 | TY/11.8 |
| CD79b |  | n/a | BioLegend | TotalSeq-C0561 | HM79-12 |
| CD80 |  | n/a | BioLegend | TotalSeq-C0849 | 16-10A1 |
| CD81 |  | n/a | BioLegend | TotalSeq-C1064 | Eat-2 |
| CD83 |  | n/a | BioLegend | TotalSeq-C0562 | Michel-19 |
| CD85k |  | n/a | BioLegend | TotalSeq-C1007 | H1.1 |
| CD86 |  | n/a | BioLegend | TotalSeq-C0200 | GL-1 |
| CD88 |  | n/a | BioLegend | TotalSeq-C1042 | 20/70 |
| CD90.2 |  | n/a | BioLegend | TotalSeq-C0075 | 30-H12 |
| CD90/CD90.1 |  | n/a | BioLegend | TotalSeq-C0380 | OX-7 |
| CD93 |  | n/a | BioLegend | TotalSeq-C0113 | AA4.1 |
| CD94 |  | n/a | BioLegend | TotalSeq-C1009 | 18d3 |
| CD95 |  | n/a | BioLegend | TotalSeq-C0917 | SA367H8 |
| CD96 |  | n/a | BioLegend | TotalSeq-C0906 | 3.3 |
| CD98 |  | n/a | BioLegend | TotalSeq-C0989 | RL388 |
| CD103 |  | n/a | BioLegend | TotalSeq-C0201 | 2~~,00E+0~~E7 |
| CD104 |  | n/a | BioLegend | TotalSeq-C1028 | 346-11A |
| CD105 |  | n/a | BioLegend | TotalSeq-C0812 | MJ7/18 |
| CD106 |  | n/a | BioLegend | TotalSeq-C0226 | 429 (MVCAM.A) |
| CD115 |  | n/a | BioLegend | TotalSeq-C0105 | AFS98 |
| CD117 |  | n/a | BioLegend | TotalSeq-C0012 | 2B8 |
| CD120b |  | n/a | BioLegend | TotalSeq-C0893 | TR75-89 |
| CD122 |  | n/a | BioLegend | TotalSeq-C0227 | 5H4 |
| CD124 |  | n/a | BioLegend | TotalSeq-C0916 | I015F8 |
| CD127 |  | n/a | BioLegend | TotalSeq-C0198 | A7R34 |
| CD134 |  | n/a | BioLegend | TotalSeq-C0195 | OX-86 |
| CD135 |  | n/a | BioLegend | TotalSeq-C0098 | A2F10 |
| CD137 |  | n/a | BioLegend | TotalSeq-C0194 | 17B5 |
| CD138 |  | n/a | BioLegend | TotalSeq-C0810 | 281-2 |
| CD140a |  | n/a | BioLegend | TotalSeq-C0573 | APA5 |
| CD146 |  | n/a | BioLegend | TotalSeq-C0134 | P1H12 |
| CD150 |  | n/a | BioLegend | TotalSeq-C0203 | TC15-12F12.2 |
| CD152 |  | n/a | BioLegend | TotalSeq-C0388 | UC10-4B9 |
| CD155 |  | n/a | BioLegend | TotalSeq-C1011 | TX56 |
| CD160 |  | n/a | BioLegend | TotalSeq-C1006 | 7H1 |
| CD163 |  | n/a | BioLegend | TotalSeq-C0417 | S15049I |
| CD169 |  | n/a | BioLegend | TotalSeq-C0440 | 3D6.112 |
| CD170 |  | n/a | BioLegend | TotalSeq-C0431 | S17007L |
| CD172a |  | n/a | BioLegend | TotalSeq-C0422 | P84 |
| CD178 |  | n/a | BioLegend | TotalSeq-C1012 | MFL3 |
| CD182 |  | n/a | BioLegend | TotalSeq-C0909 | SA044G4 |
| CD183 |  | n/a | BioLegend | TotalSeq-C0228 | CXCR3-173 |
| CD185 |  | n/a | BioLegend | TotalSeq-C0846 | L138D7 |
| CD186 |  | n/a | BioLegend | TotalSeq-C0926 | SA051D1 |
| CD192 |  | n/a | BioLegend | TotalSeq-C0426 | SA203G11 |
| CD193 |  | n/a | BioLegend | TotalSeq-C0808 | J073E5 |
| CD194 |  | n/a | BioLegend | TotalSeq-C0833 | 2G12 |
| CD196 |  | n/a | BioLegend | TotalSeq-C0225 | 29-2L17 |
| CD197 |  | n/a | BioLegend | TotalSeq-C0377 | 4B12 |
| CD198 |  | n/a | BioLegend | TotalSeq-C0907 | SA214G2 |
| CD200 |  | n/a | BioLegend | TotalSeq-C0079 | OX-90 |
| CD200R |  | n/a | BioLegend | TotalSeq-C0807 | OX-110 |
| CD201 |  | n/a | BioLegend | TotalSeq-C0439 | RCR-16 |
| CD204 |  | n/a | BioLegend | TotalSeq-C0448 | 1F8C33 |
| CD205 |  | n/a | BioLegend | TotalSeq-C1010 | NLDC-145 |
| CD207 |  | n/a | BioLegend | TotalSeq-C0437 | 4C7 |
| CD210 |  | n/a | BioLegend | TotalSeq-C1032 | 1B1.3a |
| CD223 |  | n/a | BioLegend | TotalSeq-C0378 | C9B7W |
| CD226 |  | n/a | BioLegend | TotalSeq-C0852 | 1,00E+06 |
| CD226 |  | n/a | BioLegend | TotalSeq-C0949 | TX42.1 |
| CD252 |  | n/a | BioLegend | TotalSeq-C0924 | RM134L |
| CD270 |  | n/a | BioLegend | TotalSeq-C0885 | HMHV-1B18 |
| CD272 |  | n/a | BioLegend | TotalSeq-C0881 | 6A6 |
| CD273 |  | n/a | BioLegend | TotalSeq-C0914 | TY25 |
| CD274 |  | n/a | BioLegend | TotalSeq-C0190 | MIH6 |
| CD278 |  | n/a | BioLegend | TotalSeq-C0171 | C398.4A |
| CD278 |  | n/a | BioLegend | TotalSeq-C0847 | 7E.17G9 |
| CD279 |  | n/a | BioLegend | TotalSeq-C0004 | RMP1-30 |
| CD300LG |  | n/a | BioLegend | TotalSeq-C0416 | ZAQ5 |
| CD301b |  | n/a | BioLegend | TotalSeq-C0566 | URA-1 |
| CD304 |  | n/a | BioLegend | TotalSeq-C0552 | 3,00E+12 |
| CD309 |  | n/a | BioLegend | TotalSeq-C0553 | Avas12 |
| CD314 |  | n/a | BioLegend | TotalSeq-C0835 | CX5 |
| CD317 |  | n/a | BioLegend | TotalSeq-C0811 | 927 |
| CD326 |  | n/a | BioLegend | TotalSeq-C0449 | G8.8 |
| CD335 |  | n/a | BioLegend | TotalSeq-C0184 | 29A1.4 |
| CD357 |  | n/a | BioLegend | TotalSeq-C0193 | DTA-1 |
| CD366 |  | n/a | BioLegend | TotalSeq-C0003 | RMT3-23 |
| CD370 |  | n/a | BioLegend | TotalSeq-C0556 | 7H11 |
| CD371 |  | n/a | BioLegend | TotalSeq-C0825 | 5D3/CLEC12A |
| CX3CR1 |  | n/a | BioLegend | TotalSeq-C0563 | SA011F11 |
| DLL1 |  | n/a | BioLegend | TotalSeq-C0884 | HMD1-3 |
| DR3 |  | n/a | BioLegend | TotalSeq-C0836 | 4C12 |
| ESAM |  | n/a | BioLegend | TotalSeq-C0596 | 1G8/ESAM |
| F4/80 |  | n/a | BioLegend | TotalSeq-C0114 | BM8 |
| FcεRIα |  | n/a | BioLegend | TotalSeq-C0115 | MAR-1 |
| FITC |  | n/a | BioLegend | TotalSeq-C0988 | FIT-22 |
| FR4 |  | n/a | BioLegend | TotalSeq-C1058 | TH6 |
| GPR56 |  | n/a | BioLegend | TotalSeq-C0912 | CG4 |
| H-2Kb |  | n/a | BioLegend | TotalSeq-C0231 | 25-D1.16 |
| HA.11 |  | n/a | BioLegend | TotalSeq-C1131 | 16B12 |
| I-A/I-E |  | n/a | BioLegend | TotalSeq-C0117 | M5/114.15.2 |
| IgD |  | n/a | BioLegend | TotalSeq-C0571 | 11-26c.2a |
| IgG Isotype Ctrl |  | n/a | BioLegend | TotalSeq-C0241 | HTK888 |
| IgG1 |  | n/a | BioLegend | TotalSeq-C1167 | RMG1-1 |
| IgG1, κ |  | n/a | BioLegend | TotalSeq-C0090 | MOPC-21 |
| IgG1, κ Isotype Ctrl |  | n/a | BioLegend | TotalSeq-C0236 | RTK2071 |
| IgG1, λ Isotype Ctrl |  | n/a | BioLegend | TotalSeq-C0237 | G0114F7 |
| IgG2a, κ |  | n/a | BioLegend | TotalSeq-C0091 | MOPC-173 |
| IgG2a, κ Isotype Ctrl |  | n/a | BioLegend | TotalSeq-C0238 | RTK2758 |
| IgG2b |  | n/a | BioLegend | TotalSeq-C1168 | RMG2b-1 |
| IgG2b κ |  | n/a | BioLegend | TotalSeq-C0095 | RTK4530 |
| IgG2b, κ Isotype Ctrl |  | n/a | BioLegend | TotalSeq-C0092 | MPC-11 |
| IgM |  | n/a | BioLegend | TotalSeq-C0450 | RMM-1 |
| IL-21R |  | n/a | BioLegend | TotalSeq-C0879 | 4A9 |
| IL-33Rα |  | n/a | BioLegend | TotalSeq-C0837 | DIH9 |
| Integrin β7 |  | n/a | BioLegend | TotalSeq-C0214 | FIB504 |
| JAML |  | n/a | BioLegend | TotalSeq-C0877 | 4,00E+10 |
| KLRG1 |  | n/a | BioLegend | TotalSeq-C0250 | 2F1/KLRG1 |
| Ly108 |  | n/a | BioLegend | TotalSeq-C0930 | 330-AJ |
| Ly49A |  | n/a | BioLegend | TotalSeq-C0842 | YE1/48.10.6 |
| Ly49D |  | n/a | BioLegend | TotalSeq-C0841 | 4,00E+05 |
| Ly49G |  | n/a | BioLegend | TotalSeq-C0840 | AT8 |
| Ly49H |  | n/a | BioLegend | TotalSeq-C0839 | 3D10 |
| Ly-51 |  | n/a | BioLegend | TotalSeq-C1034 | 6C3 |
| Ly-6A/E |  | n/a | BioLegend | TotalSeq-C0130 | D7 |
| Ly6C |  | n/a | BioLegend | TotalSeq-C0013 | HK1.4 |
| Ly6G |  | n/a | BioLegend | TotalSeq-C0015 | 1A8 |
| Ly-6G/Ly-6C |  | n/a | BioLegend | TotalSeq-C0116 | RB6-8C5 |
| MAdCAM-1 |  | n/a | BioLegend | TotalSeq-C0232 | MECA-367 |
| MERTK |  | n/a | BioLegend | TotalSeq-C0565 | 2B10C42 |
| NK-1.1 |  | n/a | BioLegend | TotalSeq-C0118 | PK136 |
| Notch 1 |  | n/a | BioLegend | TotalSeq-C0442 | HMN1-12 |
| Notch 4 |  | n/a | BioLegend | TotalSeq-C0888 | HMN4-14 |
| P2X7R |  | n/a | BioLegend | TotalSeq-C0824 | 1F11 |
| Rat IgG2c, κ |  | n/a | BioLegend | TotalSeq-C0240 | RTK4174 |
| Siglec H |  | n/a | BioLegend | TotalSeq-C0119 | 551 |
| TCR Va2 |  | n/a | BioLegend | TotalSeq-C0981 | B20.1 |
| TCR Vα 8.3 |  | n/a | BioLegend | TotalSeq-C0982 | B21.14 |
| TCR Vα 8.3 |  | n/a | BioLegend | TotalSeq-C0983 | KT50 |
| TCR Vα11 |  | n/a | BioLegend | TotalSeq-C0984 | RR8-1 |
| TCR Vβ5.1, 5.2 |  | n/a | BioLegend | TotalSeq-C0354 | MR9-4 |
| TCR Vβ8.1,8.2 |  | n/a | BioLegend | TotalSeq-C0235 | KJ16-133.18 |
| TCR Vγ1.1 |  | n/a | BioLegend | TotalSeq-C0209 | 2.11 |
| TCR Vγ2 |  | n/a | BioLegend | TotalSeq-C0211 | UC3-10A6 |
| TCR Vγ3 |  | n/a | BioLegend | TotalSeq-C0210 | 536 |
| TCR β chain |  | n/a | BioLegend | TotalSeq-C0120 | H57-597 |
| TCR γ/δ |  | n/a | BioLegend | TotalSeq-C0121 | GL3 |
| TCR γ/δ |  | n/a | BioLegend | TotalSeq-C0986 | UC7-13D5 |
| TER-119 |  | n/a | BioLegend | TotalSeq-C0122 | TER-119 |
| TIGIT |  | n/a | BioLegend | TotalSeq-C0848 | 1G9 |
| Tim-4 |  | n/a | BioLegend | TotalSeq-C0567 | RMT4-54 |
| VISTA |  | n/a | BioLegend | TotalSeq-C0915 | MIH63 |
| XCR1 |  | n/a | BioLegend | TotalSeq-C0568 | ZET |
| Rabbit anti-mouse STAT1 |  | n/a | Cell Signaling Technologies | 9172 | Polyclonal (9172T) |
| Rabbit anti-mouse pSTAT1 |  | n/a | Cell Signaling Technologies | 9167 | 58D6 |
| Mouse anti-mouse horseradish peroxidase (HRP)-tubulin |  | n/a | Thermo Fisher Scientific | BT7R | Polyclonal |
| Donkey anti-rabbit HRP secondary antibody |  | n/a | Jackson immunoResearch | AB10015282 | Polyclonal |

- 1. **Cell lines**

| **Name** | **Citation** | **Supplier** | **Cat no.** | **Passage no.** | **Authentication test method** |
| --- | --- | --- | --- | --- | --- |
| **/** | **/** | **/** | **/** | **/** | **/** |

- 1. **Organisms**

| **Name** | **Citation** | **Supplier** | **Strain** | **Sex** | **Age** | **Overall n number** |
| --- | --- | --- | --- | --- | --- | --- |
| WT mice |  | Janvier Laboratories | [C57BL/6J](https://janvier-labs.com/en/fiche_produit/2_c57bl-6j_mouse/) | Male/female | 8-10 weeks | 3-31 per condition (depending on the measured parameter) |
| *Ifng^-/-^* mice |  | The Jackson Laboratory | C.129S7(B6)-*Ifng^tm1Ts^*/J | Male/female | 8-10 weeks | 3-31 per condition (depending on the measured parameter) |
| *Stat1^-/-^* |  | The Jackson Laboratory (kindly provided by Dr. Andrew Brown and Dr. Andy Wullaert VIB-University of Ghent) | B6.129S(Cg)-*Stat1^tm1Dlv^*/J | Male/female | 8-10 weeks | 3-31 per condition (depending on the measured parameter) |

- 1. **Sequence based reagents**

| **Name** | **Sequence** | **Supplier** |
| --- | --- | --- |
| *Cxcl9* | Mm.PT.58.57267 | Integrated DNA Technologies |
| *Cxcl10* | Mm.PT.58.43575827 | Integrated DNA Technologies |
| *Ifit1* | Mm.PT.58.32674307 | Integrated DNA Technologies |
| *Ifng* | Mm.PT.58.41769240 | Integrated DNA Technologies |
| *Il6* | Mm.PT.58.10005566 | Integrated DNA Technologies |
| *Il12b* | Mm.PT.58.12409997 | Integrated DNA Technologies |
| *Il18* | Mm.PT.58.42776691 | Integrated DNA Technologies |
| *Irf7* | Mm.PT.58.32394021.g | Integrated DNA Technologies |
| *Isg15* | Mm.PT.58.41476392.g | Integrated DNA Technologies |
| *Siglec1* | Mm.PT.58.8446763 | Integrated DNA Technologies |
| *Tnfa* | Mm.PT.58.12575861 | Integrated DNA Technologies |

- 1. **Biological samples**

| **Description** | **Source** | **Identifier** |
| --- | --- | --- |
| **/** | **/** | **/** |

- 1. **Deposited data**

| **Name of repository** | **Identifier** | **Link** |
| --- | --- | --- |
| CBI’s Gene Expression Omnibus (GEO) repository | GSE287998 | https://www.ncbi.nlm.nih.gov/geo/query/acc.cgi?acc=GSE287998 |

- 1. **Software**

| **Software name** | **Manufacturer** | **Version** |
| --- | --- | --- |
| FlowJo | LLC | V10.9 |
| Rstudio | Open-source (Posit, PBC) | **R-4.5.1** |
| GraphPad | Prism | V10.1.0 |
| ImageJ | Open-source (National Institutes of Health) | V8.0_172 |

- 1. **Other (*e.g*. drugs, proteins, vectors etc.)**

| Anti-IFNAR antibody | MAR1-5A3 | Leinco Technologies |
| --- | --- | --- |
| Anti-IFN-γ antibody | F3 | In-house made (REGA Institute, KU Leuven) |
| Dolethal |  | Vétoquinol |
| Collagenase A |  | Roche |
| Collagenase D |  | Sigma |
| DNAse I |  | Sigma |
| Heparin |  | LEO pharma |
| Recombinant murine IFN-alpha |  | PBL |
| Recombinant murine IFN-beta |  | R&D |
| Recombinant murine IFN-gamma |  | PeproTech |
| Baricitinib-phosphate |  | MedChemExpress |
| GolgiStop (Monensin) |  | BD Biosciences |
| GolgiPlug (Brefeldin A) |  | BD Bioscienes |
| Rat serum |  | ThermoFisher scientific |
| Phosphatase inhibitor cocktail 2 |  | Sigma-Aldrich |
| Phosphatase inhibitor cocktail 3 |  | Sigma-Aldrich |
| Protease inhibitor |  | Roche |

- 1. **Please provide the details of the corresponding methods author for the manuscript:**

| Patrick Matthys: patrick.matthys@kuleuven.be |
| --- |

**2.0 Please confirm for randomised controlled trials all versions of the clinical protocol are included in the submission. These will be published online as supplementary information.**

| **/** |
| --- |
