# Supplementary material for: STAT1 drives the immune landscape of murine Toll-like receptor 9-induced liver inflammation
Source: JHEP Rep. 2025 Nov 6;8(2):101668. doi: 10.1016/j.jhepr.2025.101668 (PMC12814853; doi:10.1016/j.jhepr.2025.101668)
Supplement: Multimedia component 4 [file mmc4.pdf]

Research article

JHEP Reports

STAT1 drives the immune landscape of murine Toll-like receptor 9-induced liver inflammation

Authors

Amber De Visscher, Jarne Beliën, Eline Bernaerts, …, Charlotte L. Scott, Carine H. Wouters, Patrick Matthys

Correspondence

patrick.matthys@kuleuven.be (P. Matthys), carine.wouters@kuleuven.be (C.H. Wouters).

Graphical abstract

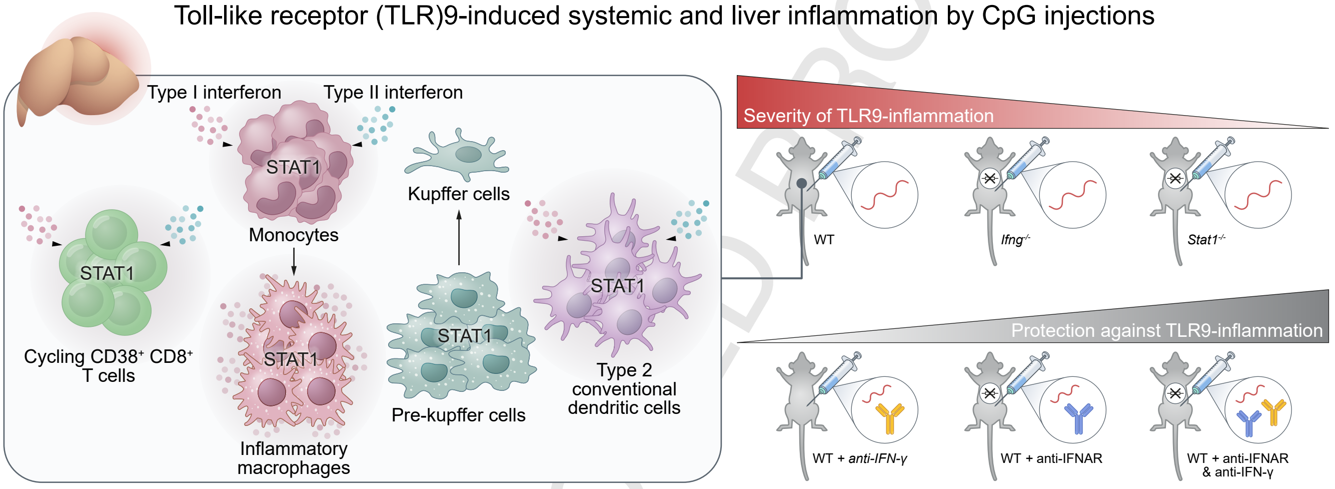

Highlights:

- Type I and II IFN-STAT1 signaling drives TLR9-induced systemic/liver inflammation.
- Liver inflammation consists of cycling T cells, transitioning monocytes, and cDC2s.
- IFNs promote the phenotype of cycling T cells and transitioning monocytes.
- Upregulated IFN-STAT1 signaling in patients with systemic liver pathology.

Impact and implications:

Our study reveals that interferon (IFN)-induced STAT1 signaling is a central mediator of both systemic and hepatic inflammation during a TLR9-induced cytokine storm. Based on these findings, we support the therapeutic use of JAK1/2 inhibitors, such as ruxolitinib and baricitinib, which indirectly suppress STAT1 activity, in patients with cytokine storm syndromes and inflammatory liver disorders to alleviate both systemic and hepatic symptoms. Notably, our data also highlight the promise of direct STAT1 inhibition as a more and potentially refined approach for intervention.

https://doi.org/10.1016/j.jhepr.2025.101668

© 2025 The Authors. Published by Elsevier B.V. on behalf of European Association for the Study of the Liver (EASL). This is an open access article under the CC BY license (http://creativecommons.org/licenses/by/4.0/).

UNCORRECTED PROOF

ARTICLE IN PRESS

Research article
Experimental and Translational Hepatology

JHEP|Reports

STAT1 drives the immune landscape of murine Toll-like receptor 9-induced liver inflammation

Amber De Visscher[1], Jarne Beliën[2], Eline Bernaerts[1], Marte Vandeput[1], Bert Malengier-Devlies[1,3], Fran Prenen[4], Hanne Meers[1], Liliana Sokol[1], Tania Mitera[1], Nele Berghmans[5], Seray Anak[6], Olivier Govaere[6], Philippe Van den Steen[4], Jochen Lamote[7,8], Niels Vandamme[9], Anna Bujko[10,11], Charlotte L. Scott[10,11], Carine H. Wouters[1,*,†], Patrick Matthys[1,*,†]

JHEP Reports **2025**. vol. ■ | 1–16

Background & Aims: Persistent activation of Toll-like receptor 9 (TLR9) has been implicated in eliciting a cytokine storm syndrome, leading to systemic and hepatic inflammation in mice and humans. This study investigates the unexplored role of STAT1, a transcription factor in pathogen-driven immune response, in mediating TLR9-induced liver inflammation.

Methods: We compared clinical, histological, and laboratory characteristics (in total nine parameters) of TLR9-induced liver inflammation between wild-type (WT) mice and STAT1-deficient (*Stat1*$^{-/-}$) mice (n = 3–31 mice/condition depending on the parameter measured) and explored their hepatic immune landscape using single-cell CITE-sequencing (total of 36,585 CD45^{+} liver cells from four to eight mice/condition). Findings were validated by flow cytometry, treatment with biologicals, *ex vivo* cell culture, and exploration of publicly available patient datasets.

Results: *Stat1*$^{-/-}$ mice are protected against TLR9-induced inflammation as they do not develop the typical features seen in WT counterparts (*p* <0.05–0.0001, depending on the parameter). This protection is associated with the absence of hepatic cycling CD38^{+}CD8^{+} T cells, type 2 conventional dendritic cells, and monocytes transitioning into inflammatory macrophages. These cell populations exhibit elevated STAT1 expression and type I and II interferon (IFN) signatures, resembling immune profiles of patients with cytokine storm syndromes and liver inflammation. *Ex vivo*, type I and II IFNs induce the phenotype of cycling T cells and transitioning monocytes through STAT1 signaling. *In vivo*, simultaneous treatment with anti-type I and II IFN antibodies in CpG-injected WT mice provided protection against systemic and liver inflammation (*p* <0.05–0.001 for five mice/condition).

Conclusions: Type I and II IFN-induced STAT1 activation drives TLR9-induced liver inflammation, and support further exploration of JAK1/2 inhibitors, which indirectly inhibit STAT1 activity, in patients with cytokine storm syndrome and other inflammatory liver disorders.

© 2025 The Authors. Published by Elsevier B.V. on behalf of European Association for the Study of the Liver (EASL). This is an open access article under the CC BY license (http://creativecommons.org/licenses/by/4.0/).

UNCORRECTED PROOF

Introduction

Over the past decade, interest in cytokine storm syndromes has intensified, particularly because of the emergence of a cytokine storm in a subset of patients with COVID-19.[1] These syndromes arise from the failure to control and terminate an immune response, leading to T cell and macrophage (Mφs) hyperactivation, which fuels a cytokine storm.[2] Patients present with systemic inflammation, characterized by fever, pancytopenia, hepatosplenomegaly, coagulopathy, and liver dysfunction. Without adequate treatment, this progresses into multi-organ failure and death.[2] Persistent activation of Toll-like receptors (TLRs), especially TLR9, has been implicated in initiating and perpetuating a cytokine storm.[3,4] Correspondingly, a sterile, well-characterized murine model based on repetitive TLR9 triggering by cytosine-phosphate-guanine (CpG) injections (developed by Behrens *et al.*[5]) phenocopies this inflammation, including liver inflammation, without the interference of infectious agents. However, sterile TLR9-induced liver inflammation remains poorly characterized.

Within the TLR9-induced cytokine storm, type II interferon (IFN-γ) has been established as a key cytokine.[5,6] Binding to its receptor activates Janus kinase (JAK)1 and JAK2, which subsequently activate Signal transducer and activator of transcription (STAT)1.[7] Notably, levels of total and phosphorylated STAT1 (pSTAT1) are elevated in livers of patients with cytokine storm syndrome,[8] implicating the JAK1/2–STAT1 axis in the disease pathogenesis. Clinical studies also demonstrated the efficacy of JAK1/2 inhibitors (*e.g.* ruxolitinib and baricitinib, which indirectly inhibit STAT1 activation) in patients with cytokine storm syndrome.[9–11] However, the precise mechanism driving remission remains incompletely understood.

In this context, Albeituni *et al.*[12] showed that ruxolitinib confers superior protection compared to IFN-γ neutralization alone in a TLR9-induced murine model, suggesting the

* Corresponding authors. Address: Laboratory of Immunobiology, Department of Microbiology, Immunology, and Transplantation, Rega Institute for Medical Research, University of Leuven, Herestraat 49, 3000 Leuven, Belgium.
E-mail addresses: patrick.matthys@kuleuven.be (P. Matthys), carine.wouters@kuleuven.be (C.H. Wouters).
† PM and CW are co-senior authors and contributed equally to this work.
https://doi.org/10.1016/j.jhepr.2025.101668

involvement of other mechanisms, for example IFN-γ-independent STAT1 signaling. Despite these insights,[9–11] the specific contribution of STAT1 and the cytokines driving its activation in cytokine storm syndromes remains unclear.

Here, we demonstrate that both type I and II IFN-induced STAT1 activation drives murine TLR9-induced systemic, and in particular liver, inflammation. In-depth transcriptomic profiling identified a distinct inflammatory landscape composed of IFN-γ-producing cycling $CD8^+$ effector T cells, type 2 conventional dendritic cells (cDC2s), and inflammatory monocytes differentiating into Mφs.

Materials and methods

Mice and *in vivo* injections

Origin and housing of mice is detailed in the Supplementary Materials and methods. The model was induced in 8–10-week-old mice by intraperitoneal (i.p.) injections of CpG (50 μg, ODN1826, IDT) every 2 days as described.[5] PBS-injected age/sex-matched mice served as controls. Our study examined male and female animals (similar findings are reported for both). The number of animals used depends on the measured parameter (n = 3–31 mice/condition). For blocking type I IFNs, mice were i.p.-injected with anti-interferon-α/β receptor (IFNAR) antibody (MAR1-5A3, Leinco Technologies, 1,000 μg/ml) kindly provided by Dr. Kai Dallmeier and Lara Kelchtermans (Laboratory of Virology & Antiviral Research, REGA institute, KU Leuven, Belgium) and for blocking type II IFNs anti-IFN-γ antibody (F3, in-house made, 1,000 μg/ml) starting 1 day before CpG injection and repeated at days 3 and 7. Experiments were approved by the Ethics Committee of KU Leuven (P223/2017 and P104/2021).

Isolation of liver non-parenchymal cells

Mice were euthanized by an i.p. injection of dolethal (Vétoquinol, 0.2 mg) and organs were dissected. Livers were fragmented in RPMI 1640 (Gibco) with Collagenase D (2 mg/ml, Sigma), DNase I (0.2 mg/ml, Sigma), and 2% heat-inactivated FBS using the GentleMACS dissociator (Miltenyi Biotec). Fragmented tissue was incubated for 30 min at 37 °C and filtered through a 70 μm cell strainer. Leukocytes were isolated via density centrifugation with 37.5% Percoll (GE Healthcare), as described.[13] For *in vivo* isolation, mice were sedated/euthanized by an i.p. injection of dolethal (0.50 mg), and cells were isolated by *ex vivo* liver perfusion as described.[14]

Blood analysis and ALT measurement

Blood samples were obtained by submandibular bleeding with heparin (500 units/ml, LEO pharma) for complete blood cell analysis (Advia 2120i hematology system, Siemens) or without heparin for alanine aminotransferase (ALT) measurement (ALT/GPT activity kit, ThermoFisher Scientific).

Histology of liver sections

Liver tissue was fixed in 10% formalin (VWR), gradually dehydrated, and embedded in paraffin. Sections (5 μm) were stained with H&E Staining Kit (4501565630, Abcam). Liver sections were blindly scored (explained in the Supplementary Materials and methods).

RNA extraction and RT-qPCR

Liver tissue was mechanically homogenized in RLT buffer from the RNeasy Mini Kit (Qiagen) and RNA was extracted according to the manufacturer's guidelines. For each sample, cDNA was synthesized from 1,000 ng RNA using the High-capacity cDNA reverse transcription Kit (Applied Biosystems). Quantitative reverse transcription-polymerase chain reaction (qRT-PCR) was performed in duplicate with primer and probe sets from Integrated DNA Technologies using an ABI Prism 7500 Sequence Detection System (Applied Biosystems). Data were normalized to the 18S ribosomal RNA levels. The fold change was compared to the average of PBS-injected wild-type (WT) mice and calculated using the $2^{-\Delta\Delta CT}$ method. Primers are listed in Table S1.

Protein extraction and Western blot

Protein extraction, SDS-PAGE, and protein transfer are described in the Supplementary Materials and methods. Membranes were blocked with 5% non-fat dry milk (BioRad) diluted in tris-buffered saline Tween (TBST) buffer for 1 h at room temperature, and incubated with rabbit anti-mouse STAT1 or pSTAT1 (Cell Signaling Technologies) or mouse anti-mouse horseradish peroxidase (HRP)-tubulin (Thermo Fisher Scientific)] overnight at 4 °C. After washing, membranes were incubated with donkey anti-rabbit HRP secondary antibody (Jackson ImmunoResearch). Membranes were exposed to a chemiluminescent substrate (SuperSignal west pico PLUS chemiluminescent substrate or SuperSignal west femto maximum sensitivity substrate, Thermo Fisher Scientific). Protein bands were captured by the Fusion Solo S (Vilber Lourmat). Between detection of pSTAT1 and STAT1, membranes were stripped with stripping buffer (0.1 M glycine, pH 2.8). Protein band intensities were measured with ImageJ. Values obtained for STAT and pSTAT were first normalized against the housekeeping protein tubulin. Outliers in the pSTAT/STAT ratio were identified by the ROUT method (Q = 1%) and excluded from the analysis.

Ex vivo cell culture

Liver cells isolated from CpG-injected WT mice through *ex vivo* digestion were cultured (10^6 cells/ml) for 24 h with IFN-α (1,000 U/ml, Pbl), IFN-β (100 ng/ml, R&D), IFN-γ (150 ng/ml, PeproTech) and/or baricitinib-phosphate (20 μM, MedChemExpress) in RPMI 1640 medium (Gibco) containing 10% FBS.

Flow cytometry

Cells were incubated with FcR blocking reagent (1:100, Miltenyi Biotec), anti-FcγRIV antibody[15] (1:100, 9E9, BioLegend) and ZombieAqua516 (1:1,000, BioLegend). Cells were stained at 4 °C during 25 min in FACS buffer (PBS with 2% FBS and 2 mM EDTA) with 25% brilliant stain buffer (BD Biosciences) and 10% rat serum (ThermoFisher Scientific). For cytokine detection, samples were incubated with GolgiStop^{TM} and GolgiPlug^{TM} protein transport inhibitors (1:1,000, BD Biosciences) for 1 h at 37 °C, and staining was performed with the Cytofix/Cytoperm^{TM} Fixation/Permeabilisation kit (BD Biosciences). For staining of STAT1, cells were fixed with 1X BD Phosflow^{TM} Lyse/Fix solution (BD Biosciences) for 10 min at 37 °C and permeabilized with BD Phosflow^{TM} Perm III. The

UNCORRECTED PROOF

antibodies are listed in Table S2. Analysis was performed on a BD LSR Fortessa X20 with DIVA software. Data were analyzed using FlowJo (version 10.9). The flowAI plugin was used to perform an automatic quality control on the FACS data which detects low-quality events by evaluating the flow rate, signal acquisition, and dynamic range, and these were removed. A representative gating strategy is depicted in Figs S6B, S7A, and S8A and C.

Single-cell transcriptomics

For FACS and CITE-seq antibody labelling, 1–2 × 10^6 liver cells of a pool from four to eight mice (1:1 male:female) were stained with fluorochrome-labelled antibodies (Table S2), Tru-Stain FcX Block (BioLegend), and oligo-conjugated antibodies (195 TotalSeq-C antibodies and 4 TotalSeq-C isotype controls, Table S3). Cell sorting was performed with BD FACS Aria Fusion. Sorted populations (69,000 CD45$^+$ living cells enriched with 1,000 CD3$^-$CD19$^-$CD45$^+$F4/80$^+$ living cells) were loaded onto a Chromium GemCode Single Cell Instrument for the formation of single-cell suspensions, followed by preparation of scRNA-seq libraries according to manufacturer's guidelines (Chromium Next GEM Single Cell 5′ Kit version 2, 1000244, and library construction kit, 1000190, 10 × Genomics). Sequencing was performed with Illumina NovaSeq flow cell at the VIB Nucleomics core. Libraries were pooled at an 85:15 ratio for 5′ gene expression and cell surface protein sequencing. The R pipeline used for subsequent analysis of

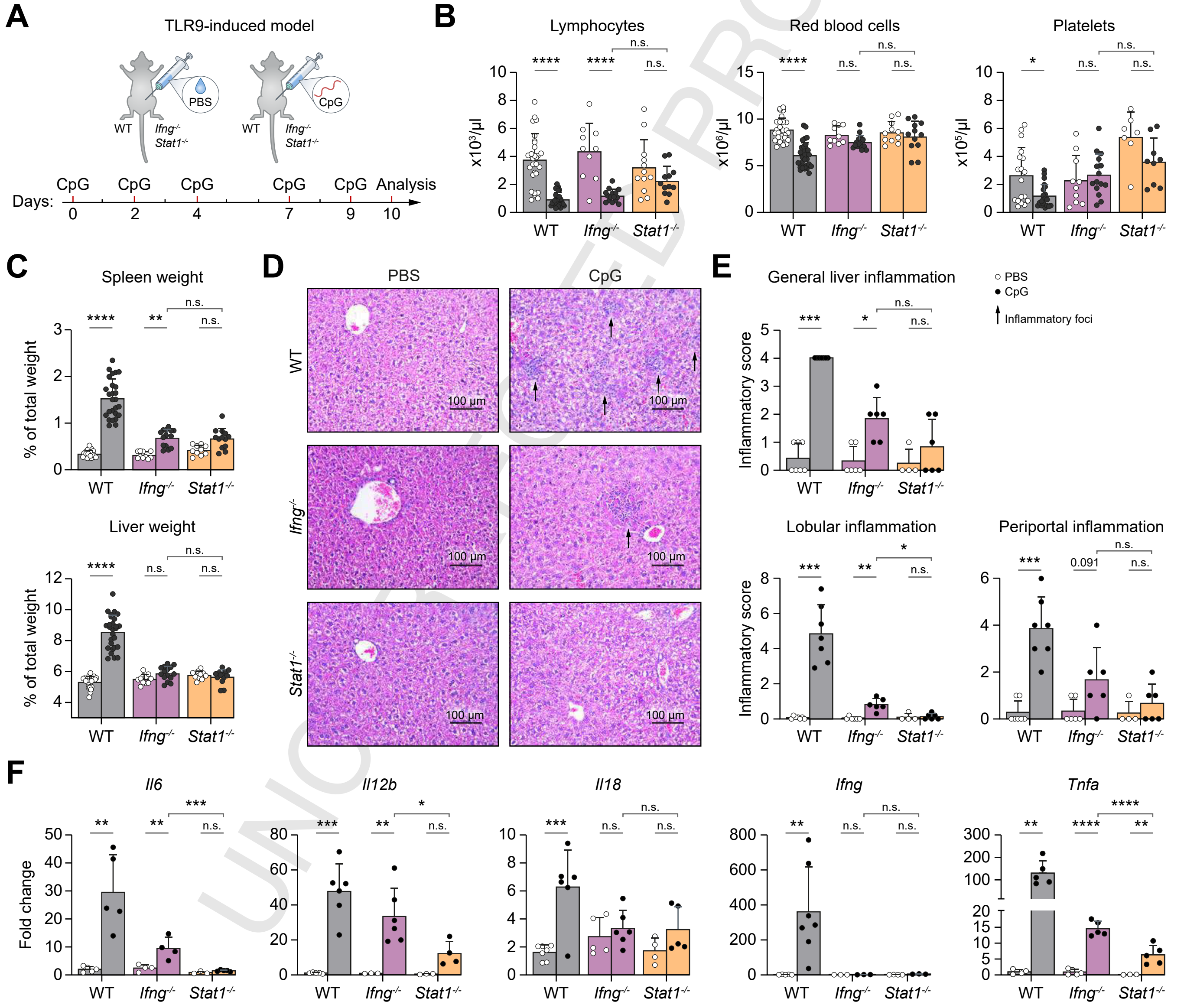

Fig. 1. STAT1 deficiency provides enhanced protection against TLR9-induced clinical features compared with IFN-γ deficiency. (A) Experimental setup. (B) Blood counts. (C) Organ/body weight. (D) Representative liver sections with H&E staining and (E) Quantification of liver inflammation. (F) Cytokine expression by qRT-PCR in livers. Bars (mean) and error bars (standard deviation). ns p>0.05, *p <0.05, **p <0.01, ***p <0.001, ****p <0.0001 (Student's t test [WT in C, F – *Il6/Il12b/Tnfa/Il18*], Mann–Whitney U test [WT in B, E, F – *Ifng*], Šídák's multiple-comparisons test [*Ifng*$^{-/-}$, *Stat1*$^{-/-}$ in B, C – liver, F – *Il6/Il12/Tnfa*], Dunn's multiple-comparisons test [*Ifng*$^{-/-}$, *Stat1*$^{-/-}$ in C – spleen, E, F – *Il18*, *Ifng*]). IFN, interferon; qRT-PCR, quantitative reverse transcription-polymerase chain reaction; STAT1, signal transducer and activator of transcription 1; TLR, Toll-like receptor; WT, wild type.

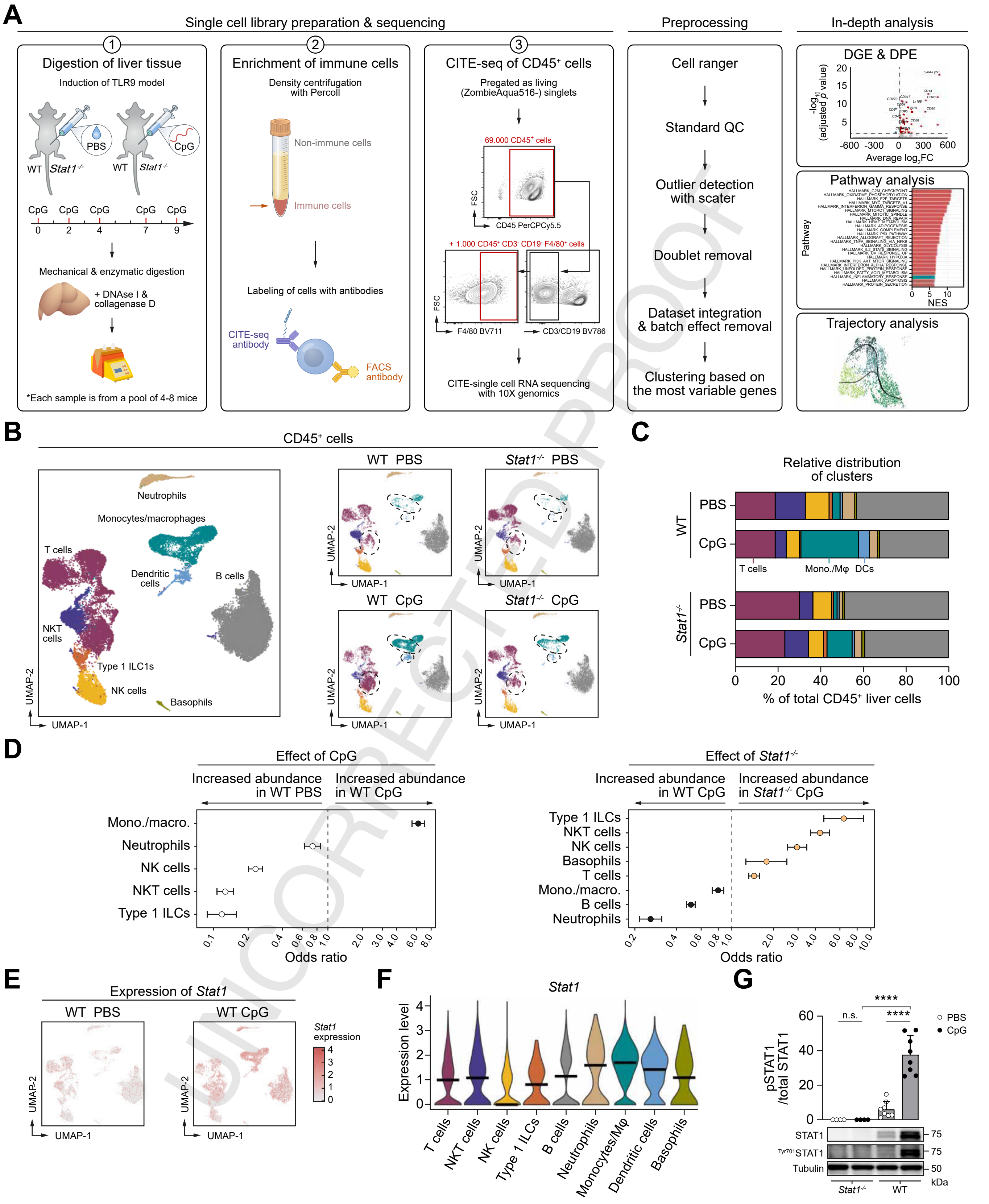

Fig. 2. Upregulation of STAT1 and changes in T cells, DCs, and monocytes/macrophages in TLR9-mediated liver inflammation. (A) Experimental setup. (B) Integrated/separate datasets from scRNA-seq. Clusters discussed in the results are circled. (C) Cluster/total CD45+ liver cells. Colors correspond with clusters in panel B. (D) Odds ratio calculated by MASC with condition (WT PBS *vs.* WT CpG) or genotype (WT CpG *vs.* *Stat1*$^{-/-}$ CpG) as contrast of interest (p <0.05). (E) *Stat1* expression. (F) *Stat1* expression in clusters of WT CpG. Colors correspond with clusters in panel B. (B) Horizontal lines (median). (G) Ratio phosphorylated-STAT1

UNCORRECTED PROOF

the CITE-seq data and exploration of publicly available human datasets is detailed in the Supplementary Materials and methods.

Statistical analysis

GraphPad Prism (version 10.1.0, GraphPad Software, San Diego, CA, USA) was used for data analysis and graphing. Outliers were identified by the ROUT method (Q = 1%) and excluded from analysis. Before statistical testing, normality was checked by the Shapiro–Wilk test. To compare two unpaired groups, the parametric Student's *t* test or non-parametric Mann–Whitney *U* test was used in case the data were normally or not normally distributed, respectively. Comparison of three or more unpaired groups was performed using one-way ANOVA followed by the Šídák's multiple-comparisons test or the non-parametric Kruskal–Wallis test followed by the Dunn's multiple-comparisons test if the data were normally or not normally distributed, respectively. For the comparison of three or more paired groups, one-way ANOVA with Geisser–Greenhouse correction followed by a Šídák's multiple-comparisons test or a Friedman test followed by Dunn's multiple-comparison test was performed if the data were normally or not normally distributed, respectively. Regarding transcriptomics, statistical tests were performed in R (version 4.2.2, R Foundation for Statistical Computing, Vienna, Austria) and is detailed in the Supplementary Materials and methods.

Results

STAT1 deficiency provides enhanced protection against clinical features of TLR9-induced liver inflammation compared to IFN-γ deficiency

Given the established role of abnormal TLR signaling in cytokine storm syndromes,[3,4] we utilized a well-characterized TLR9 model in which mice receive five i.p. injections with CpG oligonucleotides over 10 days. This induces systemic inflammation, manifesting as pancytopenia, hepatosplenomegaly, and liver inflammation, without overt liver injury[5] (normal ALT levels, Fig. S1).

To determine the contribution of STAT1 signaling induced by cytokines other than IFN-γ, we compared TLR9-induced clinical features in *Ifng*$^{-/-}$ mice with *Stat1*$^{-/-}$ mice (Fig. 1A). While *Ifng*$^{-/-}$ mice show protection against the development of anemia, thrombocytopenia, and hepatomegaly, they still present with lymphopenia and splenomegaly. In contrast, *Stat1*$^{-/-}$ mice do not develop any of these pathological features (Fig. 1B and C).

STAT1 deficiency provided enhanced protection against liver pathology compared with IFN-γ deficiency, specifically regarding the lobular inflammation (Fig, 1D and E). Correspondingly, the inflammatory cytokines *Il6*, *Il12b*, and *Tnfa*, but not *Il18*, are significantly lower in livers of *Stat1*$^{-/-}$ mice compared with *Ifng*$^{-/-}$ mice. Notably, *Stat1*$^{-/-}$ mice show no expression of *Ifng* upon TLR9 triggering (Fig. 1F).

In conclusion, although IFN-γ deficiency provides partial protection against TLR9-induced inflammation, STAT1 deficiency yields a more complete protection, particularly against liver inflammation.

Upregulation of STAT1 and changes in hepatic T cell, dendritic cell, and monocyte/macrophage populations during TLR9-induced liver inflammation

To explore the immune landscape of TLR9-induced liver inflammation and the contribution of STAT1 signaling, we performed CITE-seq on the liver from PBS-injected WT (WT PBS) and *Stat1*$^{-/-}$ mice (*Stat1*$^{-/-}$ PBS) and CpG-injected WT (WT CpG) and *Stat1*$^{-/-}$ mice (*Stat1*$^{-/-}$ CpG). Of each condition, a pool of cells (four to eight mice) was stained with FACS and CITE-seq antibodies. Sorted CD45^{+} liver cells were slightly enriched with F4/80^{+} cells (as described in the Materials and methods) to ensure a good resolution of the monocyte/Mφ compartment (Fig. 2A). A total of 9,904, 8,708, 8,069, and 9,904 cells of WT PBS, WT CpG, *Stat1*$^{-/-}$ PBS, and *Stat1*$^{-/-}$ CpG, respectively, passed quality control and were included in the analysis (Fig. 2B). Validation of the annotation of the subclusters are described in Fig. S2A and B.

CpG triggering in WT mice results in a dramatic increase in the monocyte/Mφ cluster, the dendritic cell (DC) cluster, and a subset of cells within the T cell cluster. In contrast, *Stat1*$^{-/-}$ mice are protected to some extent from those changes, maintaining an immune landscape that closely resembles that of naive mice (WT and *Stat1*$^{-/-}$ PBS) (Fig. 2B and C). Differential composition analysis demonstrated that the increased abundance of the monocyte/Mφ cluster in WT CpG is significant compared to WT PBS, as well as the decreased abundance of this cluster in *Stat1*$^{-/-}$ CpG compared with WT CpG (Fig. 2D).

Furthermore, differential gene expression (DGE) analysis showed that *Stat1* is significantly upregulated in all immune populations of WT CpG compared with WT *PBS* (Fig. 2E and Fig. S2C), with the highest expression in CpG-induced myeloid cells compared with other CpG-induced populations (Fig. 2E and F). Using Western blot on snap-frozen liver tissue, we confirmed the phosphorylation of STAT1 (pSTAT1) during TLR9 triggering in WT mice (Fig. 2G, and Figs S2D and S13). On average a 6.3-fold increase in pSTAT1/STAT1 ratio was found in CpG WT compared to PBS WT mice (average ratios for eight mice/group ± SE were 37.7 ± 11.1 and 6.0 ± 4.7, respectively).

Taken together, this identifies STAT1 as a potential driver of the expansion of leukocyte populations during CpG-induced inflammation.

STAT1 is associated with expansion of hepatic cycling T cells in TLR9-induced liver inflammation

Subclustering of the lymphocytes revealed that the CpG-induced T cell compartment consists mainly of a cycling CD8^{+} effector T (T_{eff}) cell population and two non-cycling CD8^{+} T_{eff} cell populations (Fig. 3A and B). Differential composition

(^{Tyr701}STAT1) to total STAT1 in liver lysate (calculations as described in methods) with a representative Western blot image. All uncropped Western blots are shown in Fig. S2D and S13. ns, p >0.05, ****p <0.0001 (Šídák's multiple-comparisons test [in G]). STAT1, signal transducer and activator of transcription 1; TLR, Toll-like receptor; WT, wild type.

UNCORRECTED PROOF

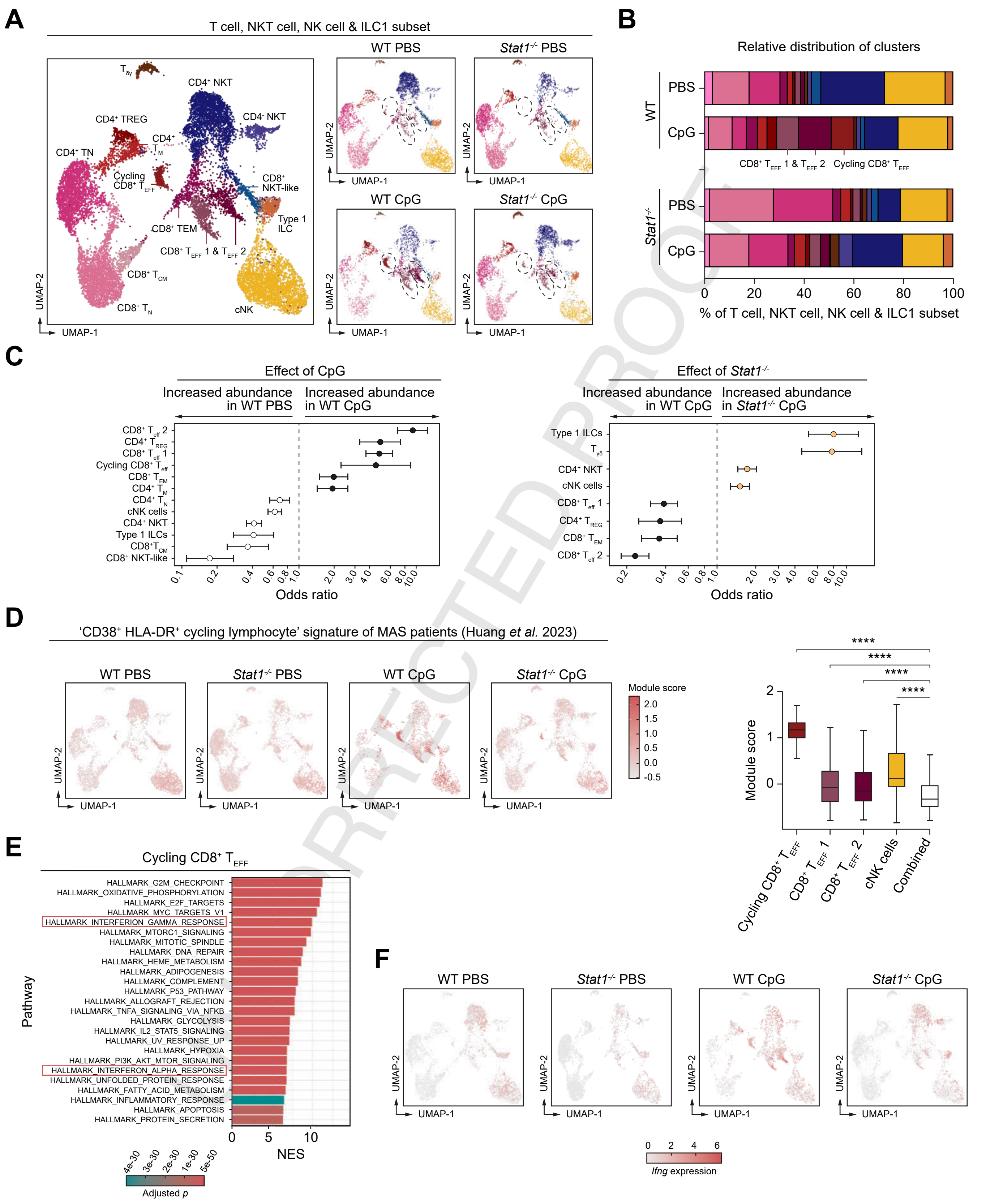

Fig. 3. STAT1 is associated with the expansion of cycling T cells in TLR9-mediated liver inflammation. Experimental setup is shown in Fig. 2A. (A) Lymphoid subclustering of integrated/separate datasets. Clusters discussed in the results are circled. (B) Cluster/total lymphocytes. Colors of clusters shown in panel A. (C) Odds ratios calculated by MASC with treatment (WT PBS *vs.* WT CpG) or genotype (WT CpG *vs.* *Stat1*$^{-/-}$ CpG) as contrast of interest (p <0.05). (D) Module score 'CD38^{+}HLA-DR^{+} cycling lymphocyte-signature' patients with MAS (Huang *et al.*[16]). Whiskers (1.5× interquartile range). Combined (remaining clusters not shown

UNCORRECTED PROOF

analysis confirmed the increased presence of all three CD8^{+} T$_{eff}$ cell populations in WT CpG compared to WT PBS. Validation of the annotation of the subclusters is shown in Fig. S3A and B.

A recent single-cell transcriptomics study from Huang *et al.*[16] identified the presence of peripheral CD38^{+} HLA-DR^{+} cycling lymphocytes, comprised of CD4^{+} T cells, CD8^{+} T cells, and cNK cells, in patients with macrophage activation syndrome (MAS), a cytokine storm syndrome which develops in the context of rheumatic disorders. Evaluation of this 'cycling lymphocyte' signature in our murine model demonstrated a clear enrichment in CpG-induced cycling CD8^{+} T$_{eff}$ cells and, to some extent, in two non-cycling CD8^{+} T$_{eff}$ cell clusters and cNK cell clusters (Fig. 3D). DGE analysis showed that the CD8^{+} T$_{eff}$ cell population 1 displays a quiescent cell state (*Klf3*), while the CD8^{+} T$_{eff}$ cell population 2 displays an inflammatory (*Ccl3* and *Ccl4*) and exhausted phenotype (*Rgs1*, *Rgs16*, *Nr4a1*, and *Nr4a2*) (Fig. S3C). To explore the signaling pathways driving the development of cycling CD8^{+} T$_{eff}$ cells, gene set enrichment analysis (GSEA) was performed. In addition to proliferation pathways, we found a type I IFN (IFN-α) and type II IFN (IFN-γ) signature (Fig. 3E), two cytokines that activate STAT1.[17] Expression of type I IFNs was not detectable in the CITE-seq data, however, type II IFNs (*Ifng*) are clearly produced by multiple lymphocyte subsets in WT CpG, particularly by CD8^{+} T$_{eff}$ cells (Fig. 3F). In both cycling and non-cycling CD8^{+} T cells, we found enriched expression of *Il10*, a cytokine that plays a protective role in TLR9-induced inflammation[5] (Fig. S3D).

In conclusion, TLR9-triggering results in the expansion of type I and II IFN-induced cycling CD8^{+} T$_{eff}$ cells, reminiscent of cycling CD38^{+}HLA-DR^{+} CD8^{+} T$_{eff}$ cells found in blood of patients with cytokine storm syndrome (MAS).

STAT1 is associated with the expansion of hepatic transitioning monocytes and type 2 cDCs in TLR9-induced liver inflammation

Subclustering of the myeloid compartment revealed the presence of Mφs, conventional type 2 DCs (cDC2), and transitioning monocytes upon TLR9 triggering (Fig. 4A and B). Differential composition analysis confirmed the enrichment of the monocyte and Mφ populations in WT CpG compared to WT PBS, but could not confirm this for the cDC2s. Similarly, the monocyte and Mφ populations are significantly reduced in *Stat1*$^{-/-}$ CpG compared with WT CpG, but this could not be supported for cDC2s (Fig. 4C). Validation of the annotation of the subclusters is depicted in Fig. S4A and B. Of note, we could not identify a Kupffer cell (KC) population. The latter can be attributed to the use of collagenase D, a rather mild collagenase, in our *ex vivo* cell isolation protocol, whereas isolation of KCs requires the harsher collagenase A.[18]

As the transitioning monocyte cluster is suggestive of an intermediate cell state between monocytes and Mφs, we performed a pseudotime trajectory analysis. Two distinct lineages are identified with the classical monocyte cluster set as starting point. Lineage 2 shows the differentiation of classical monocytes into Mφs in WT CpG, while this differentiation is blocked in *Stat1*$^{-/-}$ CpG (Fig. 4D). Expression of *Stat1* is also significantly associated with lineage 2 (Wald test = 51.02, p = 8.20 $\times$ 10^{-05}) (Fig. 4E), suggesting that STAT1 drives the monocyte-to-Mφ differentiation.

As TLR9 triggering is associated with a cytokine storm, we evaluated the expression of inflammatory cytokines and chemokines. Transitioning monocytes and Mφs from WT CpG display increased expression of several inflammatory cytokines (*Il1b*, *Il18*, and *Tnf*) and chemokines (*Ccl2*, *Ccl3*, and *Ccl4*) (Fig. 4F). Differential protein expression (DPE) analysis of this cluster between WT CpG and WT PBS further revealed the upregulation of CD317, indicative of a type I or II IFN response,[19] and Ly6A-Ly6E (SCA-1), a well-established marker of murine hematopoietic stem cells,[20] suggestive of extramedullary monocytopoiesis. Additionally, proteins indicative of an activated phenotype (CD49a, CD54, and CD80) are upregulated (Fig. 4G). Moreover, GSEA of the three TLR9-associated myeloid populations demonstrated a similar type I and II IFN signature as observed in the CD8^{+} T$_{eff}$ cell populations (Fig. 4H), suggesting that both cytokines drive STAT1 activation during monocyte-to-Mφ differentiation.

Overall, our analysis suggests that type I and II IFN-induced activation of STAT1 is driving the expansion of inflammatory transitioning monocytes during TLR9-induced liver inflammation.

STAT1 deficiency provides enhanced protection against the expansion of hepatic immune cell populations during TLR9-mediated liver inflammation in comparison to IFN-γ deficiency

The CITE-seq data predicted a type I and II IFN signature in multiple hepatic leukocyte populations associated with TLR9-induced liver inflammation. At first, we confirmed that both type I (*Irf7*, *Siglec1, and Mx1*) and II (*Cxcl9*, *Cxcl10*, and *Irf1*) IFN-stimulated genes (ISGs) are expressed during TLR9 triggering in total liver lysate using reverse transcription PCR (Fig. S5). Moreover, type I ISGs are still induced in CpG-injected *Ifng*$^{-/-}$ mice, along with a mild but significant induction of *Cxcl9*. The latter is often considered a classical type II ISG, however, it has been demonstrated that type I IFNs can induce *Cxcl9* expression in the absence of *Ifng*.[21] Expression of both type I and II ISGs is completely absent in CpG-injected *Stat1*$^{-/-}$ mice.

We also validated the CITE-seq-identified hepatic populations associated with TLR9 triggering using flow cytometry in WT, *Ifng*$^{-/-}$, and *Stat1*$^{-/-}$ mice. Because the *ex vivo* isolation protocol fails to isolate KCs, we used an alternative isolation protocol involving *ex vivo* liver perfusion with collagenase A[18] for the validation by flow cytometry (Fig. S6A). Based on the results of the CITE-seq data and literature,[18] cycling CD8^{+} T cells were identified as CD45^{+}CD19^{-}NK1.1^{-}F4/80^{-}CD3^{+}CD8^{+}Ki67^{+}CD38^{+} cells (Figs S6B and S7A and B), transitioning monocytes as CD45^{+}CD3^{-}CD19^{-}NK1.1^{-} Ly6C^{+}F4/80^{+} (Figs S6B and S8A,B), and cDC2s as CD45^{+}CD3^{-}CD19^{-}NK1.1^{-}Ly6C^{-}F4/80^{-}CD11c^{+}CD172a^{+}XCR1^{-} (Figs S6B and S8C and D).

individually). (E) GSEA from WT CpG ($p_{corrected}$ <0.05). (F) *Ifng* expression. CpG, cytosine-phosphate-guanine; GSEA, gene set enrichment analysis; MAS, macrophage activation syndrome; STAT1, signal transducer and activator of transcription 1; TLR, Toll-like receptor; WT, wild type.

UNCORRECTED PROOF

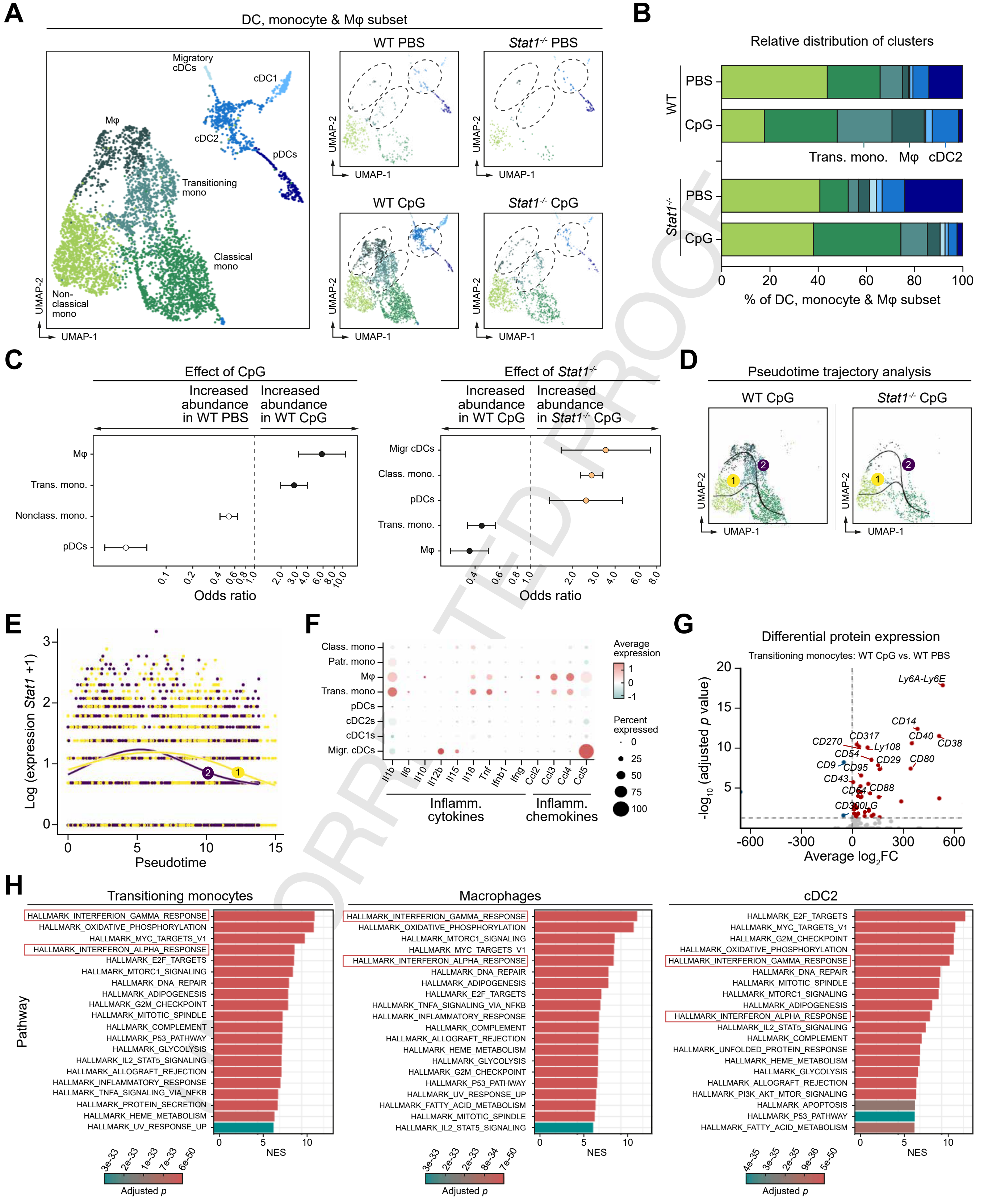

Fig. 4. STAT1 is associated with expansion of transitioning monocytes and cDC2s in TLR9-challenged livers. Experimental setup is shown in Fig. 2A. (A) Myeloid subclustering of integrated/separate datasets. Clusters discussed in the results are circled. (B) Cluster/total myeloid cells. Colors of clusters shown in panel A. (C) Odds ratios calculated by MASC with condition (WT PBS *vs.* WT CpG) or genotype (WT CpG *vs.* $Stat1^{-/-}$ CpG) as contrast of interest (p <0.05). (D) Pseudotime trajectory analysis with (E) *Stat1* expression. (F) Cytokine/chemokine expression in WT CpG. (G) Differential protein expression from transitioning monocytes (WT PBS *vs.* WT CpG). (H) GSEA from WT CpG ($p_{corrected}$ <0.05). CpG, cytosine-phosphate-guanine; DC, dendritic cell; GSEA, gene set enrichment analysis; Mφ, macrophage; STAT1, signal transducer and activator of transcription 1; TLR, Toll-like receptor; WT, wild type.

We confirmed the expansion of all three CpG-induced populations in WT mice and their absence in *Stat1*$^{-/-}$ mice, while there is a limited but still significant increase in *Ifng*$^{-/-}$ mice. Likewise, expression of STAT1 is highly induced in these cells from both WT and *Ifng*$^{-/-}$ mice, but less potently in *Ifng*$^{-/-}$ mice compared with WT mice. This is associated with an increased expression of CD317,[19,22] confirming that CpG-induced cells underwent IFN stimulation in both WT and *Ifng*$^{-/-}$ mice, while all IFN signaling is blocked in *Stat1*$^{-/-}$ mice. Cycling T cells and transitioning monocytes are not found in PBS-injected mice or CpG-injected *Stat1*$^{-/-}$ mice. Therefore, comparisons were made with the F4/80$^-$ monocytes and the non-cycling T cells for transitioning monocytes and cycling T cells, respectively. Also, for cDC2s, CD317 expression could not validate the effect of IFN as it is expressed on DCs during homeostasis[19,22] (Fig. 5A–C and Figs S7A, S8A and C, and S9A–C). Moreover, hepatic cycling T cells and cNK cells are sources of type II IFNs (IFN-γ) (Fig. S9), as observed in the transcriptome data (Fig. 3F).

Because KCs could not be studied in the CITE-seq data, we investigated KCs (CD45$^+$CD3$^-$CD19$^-$NK1.1$^-$Ly6C$^-$F4/80$^+$VSIG4$^+$CLEC2$^+$) in this flow cytometry dataset (Fig. S6A and B). Surprisingly, VSIG4$^+$ KCs are severely depleted upon CpG triggering (Fig. 6A), while a population reminiscent of pre-KCs (CD45$^+$CD3$^-$CD19$^-$NK1.1$^-$Ly6C$^-$F4/80$^+$VSIG4$^-$CLEC2$^+$) is induced (Fig. 6B). Pre-KCs exhibit elevated expression of STAT1 and CD317 in both WT and *Ifng*$^{-/-}$ mice, while *Stat1*$^{-/-}$ mice are protected from these changes (Fig. 6D and Fig. S11A). Non-KC Mφs (CD45$^+$CD3$^-$CD19$^-$NK1.1$^-$Ly6C$^-$F4/80$^+$VSIG4$^-$CLEC2$^-$) expressing STAT1 and CD317, corresponding to the identified Mφs in the CITE-seq data, are still present in *Ifng*$^{-/-}$ mice, but completely absent in *Stat1*$^{-/-}$ mice (Fig. 6C and E and Fig. S11B).

To investigate whether type I and II IFNs stimulate the phenotype of the TLR9-associated hepatic leukocytes, cells from CpG-injected WT mice were cultured and stimulated with IFNs for 24 h (Fig. 7A). Using baricitinib, we also evaluated their dependence on STAT1 signaling. Both type I or II IFNs are able to promote the ‘cycling’ phenotype of CD8$^+$ T cells and the ‘transitioning’ phenotype of monocytes *ex vivo* (Fig. 7B and C). However, in case of the cDC2s, IFNs do not promote their phenotype, suggestive of their indirect effect on cDC2s *in vivo* (Fig. 7D).

Taken together, using flow cytometry, we validated the expansion of cycling T cells and transitioning monocytes, which are both stimulated by type I and II IFNs and STAT1 signaling, as well as the expansion of cDC2s during TLR9 triggering in WT mice.

Pharmacological targeting of both type I and II interferons prevents clinical features of TLR9-induced inflammation and the expansion of hepatic leukocyte populations

To confirm that both type I and II IFNs drive TLR9-associated features, we treated mice with blocking anti-IFNAR antibodies and a neutralizing anti-IFN-γ antibody (Fig. S6A).

CpG-injected WT mice receiving double treatment, show improvement in pancytopenia (Fig. 8A), hepatosplenomegaly (Fig. 8B), and liver inflammation (Fig. 8C and D), with reduced expression of inflammatory cytokines (*Il6*, *Il12b*, and *Tnfa*) (Fig. 8E) compared with untreated mice. In contrast, anti-IFN-γ treatment alone significantly improves hepatosplenomegaly (Fig. 8B–D), while single treatment with anti-IFNAR only improves thrombocytopenia and minimizes the expression of inflammatory cytokines (*Il6*, *Il12b*, and *Tnfa*).

Similarly, expansion of hepatic leukocyte populations associated with TLR9 triggering is more inhibited in CpG-injected WT mice with the combined treatment, compared with anti-IFN-γ antibody, hence mimicking the *Stat1*$^{-/-}$ mice (Figs 5A–C, 6A–E and Figs S7A, S8A and C, S9A–C, and S11A–C).

In conclusion, simultaneous pharmacological inhibition of type I and II IFNs in CpG-injected WT mice phenocopies the protective phenotype of *Stat1*$^{-/-}$ mice against TLR9-associated clinical features and hepatic immune populations.

Type I and II IFN signature in blood and liver from patients with a TLR9-implicated pathology

To confirm the clinical relevance of our findings, we explored publicly available datasets of patients with a cytokine storm syndrome (MAS)[16] and liver inflammation (primary sclerosing cholangitis [PSC], which is characterized by chronic inflammation and irreparable damage to the bile ducts).[23] Importantly, both disorders are characterized by a TLR9-implicated pathology.[2,5,24,25] In peripheral blood mononuclear cells (PBMCs) of patients with MAS, RNA-seq showed significantly enriched type I and II IFN signatures (Fig. S12A). Additionally, in liver non-parenchymal cells from patients with PSC, expression of type I and II ISGs, and STAT1 are significantly enriched in several leukocyte populations, including the cycling and CD8$^+$ T cells, plasmacytoid dendritic cell (pDCs), monocytes, and KCs (Fig. S12B).

In conclusion, type I and II IFN signatures in blood and liver from patients with a TLR9-implicated pathology validate the clinical relevance of our findings with the murine TLR9 model.

Discussion

Persistent TLR triggering initiates and perturbates a cytokine storm syndrome, a severe complication associated with infections, malignancies, and autoinflammatory disorders. Using a well-characterized model of TLR9-induced inflammation, our analysis demonstrated that type I and II IFN-induced STAT1 signaling drives the TLR9-associated liver inflammatory landscape, characterized by the presence of cycling CD8$^+$ T cells, transitioning monocytes, and cDC2s.

The role of type I IFNs in TLR9-induced liver inflammation has been very scarcely investigated. Canna *et al.*[26] demonstrated that *Ifnar*$^{-/-}$ mice are partially protected from TLR9-associated clinical features, including mildly but significantly reduced liver inflammation compared with WT mice. As CpG-injected *Ifnar*$^{-/-}$ mice still exhibited increased levels of type II IFNs,[26] this may drive the remaining liver inflammation, which is in line with our findings using the anti-IFNAR antibody treatment. Correspondingly, Huang *et al.*[16] recently identified both type I and II IFN signatures in PBMCs of patients with MAS. Additionally, in a publicly available dataset of hepatic leukocyte populations of patients with liver inflammation, we identified both type I and II IFN signatures, which further underscores the clinical relevance of our findings. It has been shown that type I IFNs prime the type II IFN-induced signaling pathway by upregulating the expression of STAT1.[27] In agreement with this, we found increased STAT1 expression in

UNCORRECTED PROOF

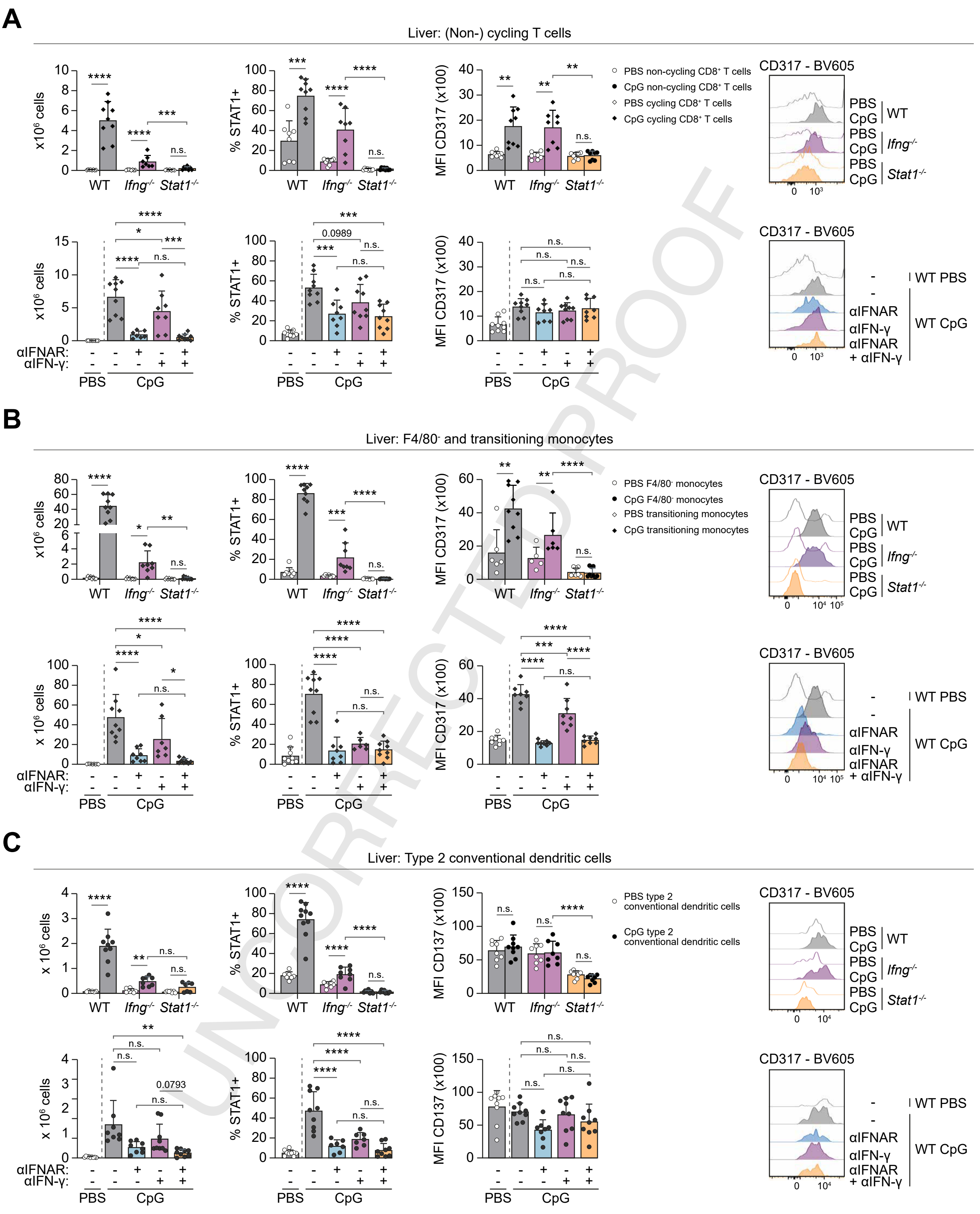

Fig. 5. Type I and II interferons drive development of hepatic TLR9-associated leukocyte populations. Experimental setup and gating strategy are shown in Fig. S6. Absolute number of cells, %STAT1$^+$ and MFI CD317 expression with representative histograms of (A) (non-)cycling T cells, (B) F4/80$^-$/transitioning monocytes, and (C) cDC2. Bars (mean) and error bars (standard deviation). ns *p* >0.05, **p* <0.05, ***p* <0.01, ****p* <0.001, *****p* <0.0001 (Student's *t* test [WT in A, B – counts, C], Mann–Whitney *U* test (WT in B – STAT1/CD317), Šidák's multiple-comparisons test [*Ifng*$^{-/-}$, *Stat1*$^{-/-}$ in A – counts/STAT1, B – STAT1, C – STAT1/CD317; treatment

UNCORRECTED PROOF

all three TLR9-associated hepatic leukocyte populations of CpG-injected *Ifng*$^{-/-}$ mice, in which type I IFN signaling is intact. Furthermore, Weaver *et al.*[28] have shown that injecting healthy WT mice with solely type II IFNs could not recapitulate the clinical features of TLR9-induced inflammation. Instead, the interplay between type II IFNs and TLR9 signaling was required to induce systemic and hepatic inflammation.[28] Based on our observations, we assume that type I IFNs induced by TLR9 triggering is particularly required. Interestingly, IFNAR and IFN-γ deficiency have been shown to protect against hepatic inflammation in other disease contexts, such as experimental severe malaria, and PBMCs of patients with malaria also exhibit a high type I IFN signature.[29]

We identified that TLR9-induced liver inflammation is dominated by a monocyte population differentiating into Mφs, which we termed transitioning monocytes. STAT1 is significantly associated with their differentiation, which is in accordance with the established role of STAT1 in Mφ differentiation and function.[30–32] Interestingly, blood monocytes of patients with cytokine storm syndrome (MAS) display a similar increase in basal levels of pSTAT1, and their monocytes are hyperresponsive to type II IFN stimulation, resulting in even higher pSTAT1 levels.[33] Whether this type II IFN hyperresponsiveness is mediated by type I IFN priming was not explored by the authors. Verweyen *et al.*[34] showed that TLR-induced IL-18 production from monocytes is strongly enhanced when primed with type I IFNs. In line with this, we found that anti-IFNAR, but not anti-IFN-γ treatment, inhibits IL-18 production in the liver upon TLR9 triggering. Furthermore, our transcriptome data showed that the CpG-induced transitioning monocytes and Mφs both display high expression of *Il18*. In addition, we found increased expression of *Tnf* and, to a lesser extent, of *Il6*, which is reminiscent of the interleukin (IL)-6^{+} and TNF-α^{+} Mφs found in liver biopsies of patients with cytokine storm syndrome (MAS).[35]

Regarding the origin of monocyte expansion, Weaver *et al.* showed that a CCR2-independent mechanism of extramedullary monocytopoiesis drives their expansion during TLR9 triggering.[36] With both our cell isolation protocols, we show monocyte expansion, indicating that they are not merely enriched in the blood but also expand within the liver tissue itself. Additionally, we found the increased expression of Sca-1, a marker of murine hematopoietic stem cells,[20] on the transitioning monocytes, further supporting the hypothesis of TLR9-induced extramedullary monocytopoiesis. In the same study, it was shown that inflammatory monocyte expansion is not altered in *Ifng*$^{-/-}$ and *Ifnar*$^{-/-}$ mice during TLR9 triggering,[36] while we found their significant reduction in *Ifng*$^{-/-}$ and *Stat1*$^{-/-}$ mice or with anti-IFN-γ and anti-IFNAR treatment in WT mice.[36] Nevertheless, inflammatory monocytes were defined as CCR2^{+}Ly6C^{hi} cells, only representing a subset of monocytes, whereas we defined our transitioning monocytes as Ly6C^{+}F4/80^{+} based on our CITE-seq results.

With our *ex vivo* digestion protocol, we found that liver-resident KCs are depleted during TLR9-induced liver inflammation. Instead, another KC population is induced, expressing CLEC2 but not VSIG4, and therefore reminiscent of pre-moKCs, which are recruited to replenish the KC pool.[37] This so-called 'Mφ disappearance reaction' has been described in a variety of inflammatory liver disorders.[38–42] Interestingly, type I IFNs have been implicated in delaying KC replenishment during viral hepatitis.[38]

Recent patient studies described a peripheral CD38^{+}HLA-DR^{+}CD8^{+} T cell population,[43,44] with increased expression of genes implicated in cell proliferation in patients with cytokine storm syndrome (MAS).[16] Likewise, we found that TLR9-associated hepatic CD8^{+} T cells express CD38 and display an overall gene signature that strongly resembles those of patient cells. With our CITE-seq data, we found that cycling and non-cycling T cells, as well as natural killer (NK) cells, are the main IFN-γ-producing cells. Additionally, Rood *et al.*[45] identified a unique dual IL-10- and IFN-γ-producing CD8^{+} T_{eff} cell population with high turnover in livers of CpG-injected WT mice. Similarly, we found *Il10* expression in the TLR9-associated (non-)cycling CD8^{+} T_{eff} cell populations, suggesting that they correspond to this population. Interestingly, induction of IL-10 was shown to be antigen-independent but partially type II IFN-dependent.[45] Whether type I IFNs are the remaining factor mediating their expansion was not evaluated by the authors. However, Huang *et al.*[16] demonstrated the importance of type I IFNs, as *in vitro* stimulation of healthy donor CD8^{+} T cells with IFN-α2 resulted in the generation of CD38^{+}HLA-DR^{+}CD8^{+} T cells.

Data on cDCs in TLR9-induced liver inflammation are scarce, and their role in patients with cytokine storm syndrome remains unclear. Behrens *et al.*[5] suggested a role for cDCs in the early production of type II IFNs, with late-phase production taken over by NK and T cells, as we confirm here. Within the DC population, we identified that cDC2s are particularly expanding during TLR9 triggering. Interestingly, cDC2s have been described to provoke a proinflammatory environment and aid in the recruitment of Mφs into the liver.[46] Additionally, STAT1 gain-of-function has been shown to skew DCs to a proinflammatory phenotype and causes them to lose their tolerogenic properties.[47] Therefore, the excessive STAT1 activation during TLR9 triggering may induce such an inflammatory phenotype.

In summary, we show that type I and II IFN-induced STAT1 activation drives TLR9-associated features, particularly liver inflammation, which is characterized by IFN-γ-producing cycling T cells, cDC2s, and monocytes transitioning into inflammatory Mφs. Our findings further support the therapeutic use of JAK1/2 inhibition in patients with refractory cytokine storm syndromes. Moreover, we highlight the potential of novel therapeutic strategies beyond type II IFNs neutralization such as targeting type I IFNs or safely inhibiting STAT1. Given that STAT1 acts downstream from JAKs, its inhibition may offer a more refined approach with potentially fewer off-target effects than broad JAK blockade. Nonetheless, further preclinical research on these therapies is warranted to ensure their safe application in the clinic.

UNCORRECTED PROOF

in A, B, C – counts/STAT1], Dunn's multiple-comparisons test [*Ifng*$^{-/-}$, *Stat1*$^{-/-}$ in A – CD317, B – counts/CD317, C – counts; treatment in C – counts]). DC, dendritic cell; STAT1, signal transducer and activator of transcription 1; TLR, Toll-like receptor; WT, wild type.

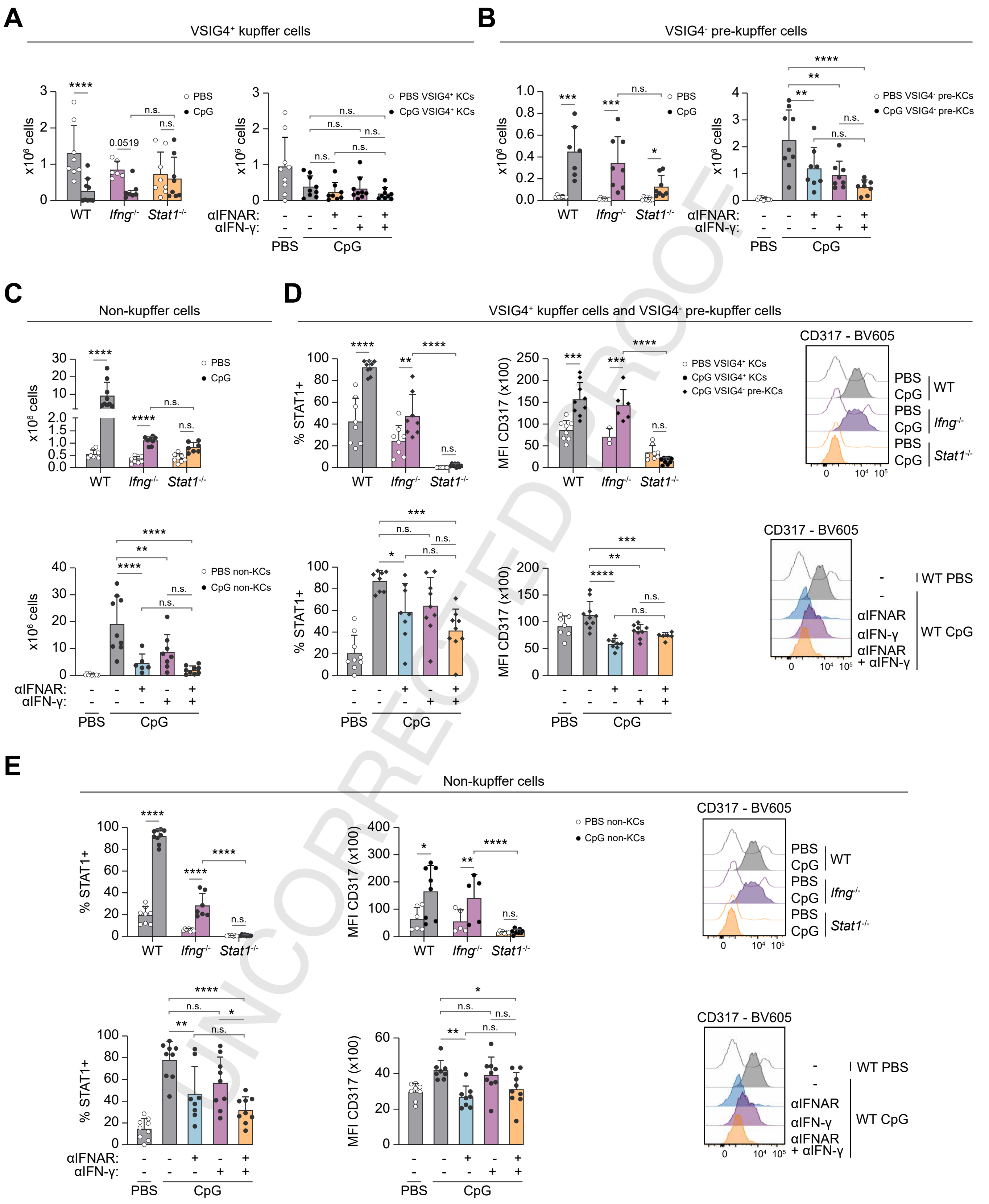

Fig. 6. Loss of Kupffer cells and expansion of non-Kupffer cells in TLR9-induced liver inflammation. Experimental setup and gating strategy are shown in Fig. S6A and B. Absolute numbers of (A) VSIG4+ KCs, (B) VSIG4- pre-KCs, and (C) non-KCs. (D&E) %STAT1+ cells and MFI CD317 expression with representative histograms of (D) VSIG4+ KCs and VSIG4- pre-KCs and (E) non-KCs. Bars (mean) and error bars (standard deviation). ns p >0.05, *p <0.05, **p <0.01, ***p <0.001, ****p <0.0001 (Student's t test [WT in B, D, E], Mann–Whitney U test [WT in A, C], Šídák's multiple-comparisons test [*Ifng*$^{-/-}$, *Stat1*$^{-/-}$ in A, C–E; treatment in B–E],

UNCORRECTED PROOF

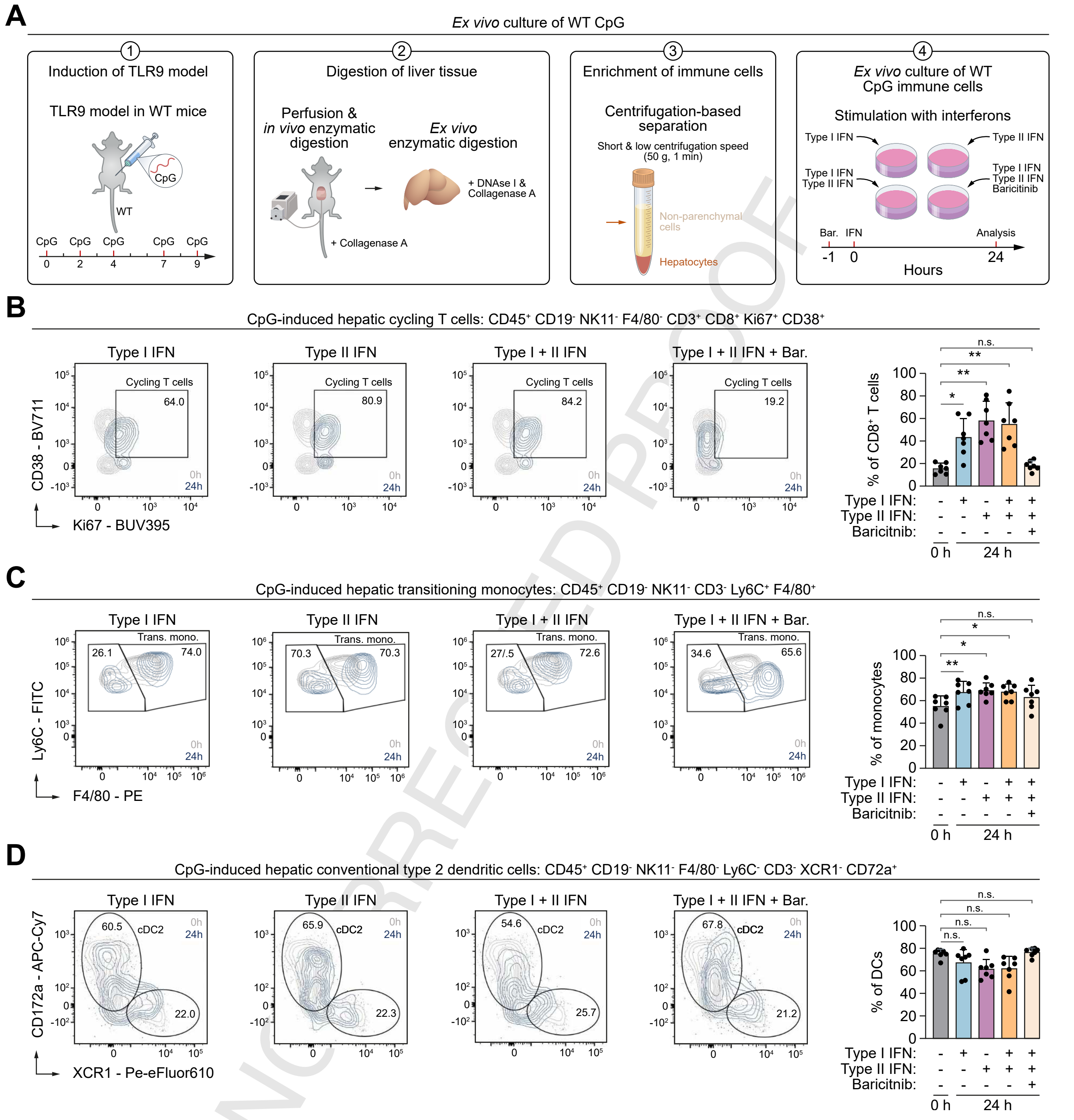

Fig. 7. IFN-induced STAT1 signaling promotes the phenotype of cycling T cells and transitioning monocytes *ex vivo*. (A) Experimental setup: isolation of liver cells from CpG-injected WT mice followed by *ex vivo* stimulation with IFNs and/or baricitinib. (B–D) Representative flow plots and percentages of indicated populations. Grey plots (CpG-induced cells, no *ex vivo* stimulation) and blue plots (CpG-induced cells, 24 h *ex vivo* stimulation with IFNs and/or baricitinib). Bar graphs on the right: bullets represent each a single culture, and bars represent mean with standard deviation. ns p >0.05, *p <0.05, **p <0.01, ***p <0.001, (Šídák's multiple-comparisons test [cycling T cells in B, transitioning monocytes in C], Dunn's multiple-comparisons test [cDCs in D]). cDC, conventional dendritic cell; CpG, cytosine-phosphate-guanine; IFN, interferon; STAT1, signal transducer and activator of transcription 1; WT, wild type.

Dunn's multiple-comparisons test [*Ifng*$^{-/-}$, *Stat1*$^{-/-}$ in A-B; treatment in A, C]). CpG, cytosine-phosphate-guanine; KC, Kupffer cell; STAT1, signal transducer and activator of transcription 1; TLR, Toll-like receptor; WT, wild type.

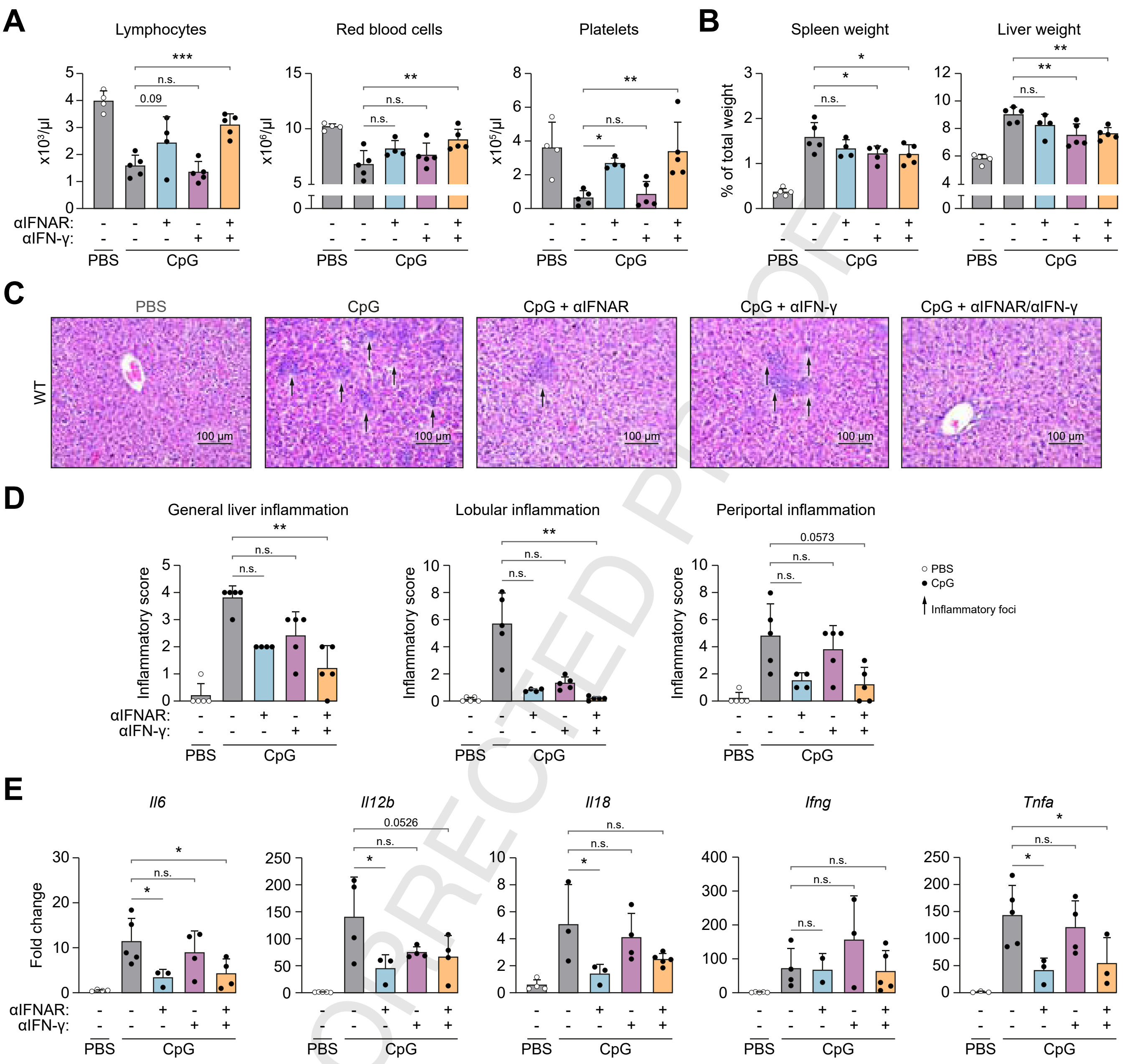

Fig. 8. Pharmacological targeting of type I and II interferons prohibits TLR9-induced clinical features. Mice were treated with αIFNAR, αIFN-γ or both, as described in the Materials and methods and shown in experimental setup in Fig. S6A. (A) Blood counts. (B) Percentages of organ *vs.* body weight. (C) Representative liver sections with H&E staining and (C) quantification of liver inflammation. (E) Fold change expression total liver lysate. Bars (mean) and error bars (standard deviation). ns p >0.05, *p <0.05, **p <0.01, ***p <0.001 [Šídák's multiple-comparisons test (A, B, E), Dunn's multiple-comparisons test (D, E – Ifng)]. CpG, cytosine-phosphate-guanine; IFN, interferon; IFNAR, interferon-α/β receptor; TLR, Toll-like receptor.

Affiliations

[1]*Laboratory of Immunobiology, Department of Microbiology, Immunology, and Transplantation, Rega Institute for Medical Research, University of Leuven, Leuven, Belgium;* [2]*Laboratory for Neuroimmunology, Department of Neurosciences, Leuven Brain Institute, KU Leuven, Leuven, Belgium;* [3]*Centre for Reproductive Health, Institute for Regeneration and Repair, University of Edinburgh, Edinburgh, UK;* [4]*Laboratory of Immunoparasitology, Department of Microbiology, Immunology, and Transplantation, Rega Institute for Medical Research, KU Leuven, Leuven, Belgium;* [5]*Laboratory of Molecular Immunology, Department of Microbiology, Immunology, and Transplantation, Rega Institute for Medical Research, KU Leuven, Leuven, Belgium;* [6]*Translational Cell and Tissue Research unit, Department of Imaging & Pathology, KU Leuven, Leuven, Belgium;* [7]*VIB Flow Core Leuven, VIB Technologies, Leuven, Belgium;* [8]*VIB-KU Leuven Center for Cancer Biology, Department of Oncology, Biomedical Science Group, KU Leuven, Leuven, Belgium;* [9]*VIB Single Cell Core, VIB, Leuven-Ghent, Belgium;* [10]*Laboratory of Myeloid Cell Biology in Tissue Damage and Inflammation, VIB-UGent Centre for Inflammation Research, Technologiepark-Zwijnaarde, Ghent, Belgium;* [11]*Department of Biomedical Molecular Biology, Faculty of Sciences, Ghent University, Ghent, Belgium*

UNCORRECTED PROOF

Abbreviations

ALT, alanine aminotransferase; cDC, conventional dendritic cell; CpG, cytosine-phosphate-guanine; DC, dendritic cell; DGE, differential gene expression; DPE, differential protein expression; GSEA, gene set enrichment analysis; HRP, horseradish peroxidase; i.p., intraperitoneal; IFN, interferon; IFNAR, interferon-α/β receptor; ISGs, IFN-stimulated genes; JAK, Janus kinase; KC, Kupffer cell; MAS, macrophage activation syndrome; Mφ, macrophage; NK, natural killer; PBMCs, peripheral blood mononuclear cells; pDC, plasmacytoid dendritic cell; PSC, primary sclerosing cholangitis; pSTAT1, phosphorylated signal transducer and activator of transcription 1; qRT-PCR, quantitative reverse transcription-polymerase chain reaction; STAT1, signal transducer and activator of transcription 1; TBST, tris-buffered saline Tween; T_{eff}, effector T; TLR, Toll-like receptor; WT, wild-type.

Financial support

This work was supported by grants from the Research Foundation Flanders (FWO) (G0A3218N) and a C1 grant (C16/17/010 and C14/23/143) of KU Leuven. ADV, JB, and EB received an FWO fellowship for fundamental research (11K0722N and 11K0724N [to ADV], 11A0523N and 11A0525N [to JB], 11H9123N and 11H9125N [to EB]).

Conflicts of interest

CW has received consultancy fees (Novartis, Sobi, and UCB) and unrestricted grants from Novartis, Roche, GlaxoSmithKline, and Pfizer paid to her institution. The authors have no additional financial interests.

Please refer to the accompanying ICMJE disclosure forms for further details.

Authors' contributions

Conceptualization: ADV, JB, BM-D, FP, CHW, PM. Resources: PM. Methodology: ADV, JB, EB, MV, SA, OG, JL, NV, AB, CLS. Investigation: ADV, JB, EB, MV, FP, HM, LS, TM, NB, SA, OG, JL, NV. Formal analysis: ADV. Supervision: CHW, PM. Project administration: PM. Writing – original draft: ADV. Writing – review and editing: JB, EB, MV, BM-D, FP, PVS, AB, CLS, CHW, PM. Funding acquisition: ADV, JB, EB, CHW, PM.

Data availability

All data that support the main findings in this study are available in the manuscript or the supplementary materials. Raw data supporting the conclusions in this manuscript will be made available by the authors, without undue reservation, to any qualified researcher. The corresponding author (PM) can be contacted via e-mail (patrick.matthys@kuleuven.be) and will provide files by written request. The raw transcriptomics data has been uploaded to CBI's Gene Expression Omnibus (GEO) repository under the accession number GSE287998.

Acknowledgements

The authors thank Prof. Dr. Roskams for advising on the histological analysis. We thank Erik Martens, Anke Vandekeere, and Max Basstanie for their assistance with animal studies and sample preparations. We express our gratitude to Kourosh Ahmadzadeh for the helpful discussions and Dr. Kai Dallmeier for providing the anti-IFNAR antibody. We are also thankful to the VIB Single Cell Core, VIB Flow Core Ghent, and VIB Nucleomics for support and access to the instrument park (vib.be/core-facilities), and particularly Ria Roelandt, Jana Roels, and Janick Mathys. Figs 1A, 2A, 7A, and Fig. S6A were partially created with BioRender.

Supplementary data

Supplementary data to this article can be found online at https://doi.org/10.1016/j.jhepr.2025.101668.

References

Author names in bold designate shared co-first authorship

[1] **Vanderbeke L**, **Van Mol P**, **Van Herck Y**, et al. Monocyte-driven atypical cytokine storm and aberrant neutrophil activation as key mediators of COVID-19 disease severity. Nat Commun 2021;12:4117.

[2] Brisse E, Wouters CH, Matthys P. Advances in the pathogenesis of primary and secondary haemophagocytic lymphohistiocytosis: differences and similarities. Br J Haematol 2016;174:203–217.

[3] Yanagimachi M, Naruto T, Miyamae T, et al. Association of IRF5 polymorphisms with susceptibility to macrophage activation syndrome in patients with juvenile idiopathic arthritis. J Rheumatol 2011;38:769–774.

[4] Fall N, Barnes M, Thornton S, et al. Gene expression profiling of peripheral blood from patients with untreated new-onset systemic juvenile idiopathic arthritis reveals molecular heterogeneity that may predict macrophage activation syndrome. Arthritis Rheumatol 2007;56:3793–3804.

[5] Behrens EM, Canna SW, Slade K, et al. Repeated TLR9 stimulation results in macrophage activation syndrome-like disease in mice. J Clin Invest 2011;121:2264–2277.

[6] De Benedetti F, Grom AA, Brogan PA, et al. Efficacy and safety of emapalumab in macrophage activation syndrome. Ann Rheum Dis 2023;82:857–865.

[7] **Hu X**, **li J**, **Fu M**, et al. The JAK/STAT signaling pathway: from bench to clinic. Signal Transduct Target Ther 2021;6:402.

[8] **Prencipe G**, **Bracaglia C**, Caiello I, et al. The interferon-gamma pathway is selectively up-regulated in the liver of patients with secondary hemophagocytic lymphohistiocytosis. PLoS One 2019;14:e0226043.

[9] Song Z, Yao H, Jin Y, et al. Ruxolitinib as a salvage therapy in adult-onset macrophage activation syndrome: insights from eight cases. Ann Rheum Dis 2024;83:1798–1799.

[10] Liao J, Tang Q, Xie X, et al. The efficacy and safety of JAK inhibitors in patients with adult-onset Still's disease: a meta-analysis and systematic review. Int Immunopharmacol 2024;137:112451.

[11] Levy O, Apel A, Alhdor H, et al. Ruxolitinib for refractory macrophage activation syndrome complicating adult-onset Still's disease. Eur J Rheumatol 2023;9:217–220.

[12] Albeituni S, Verbist KC, Tedrick PE, et al. Mechanisms of action of ruxolitinib in murine models of hemophagocytic lymphohistiocytosis. Blood 2019;134:147–159.

[13] De Visscher A, Vandeput M, Vandenhaute J, et al. Liver type 1 innate lymphoid cells undergo apoptosis in murine models of macrophage activation syndrome and are dispensable for disease. Eur J Immunol 2024;54:e2451043.

[14] Scott CL, Zheng F, De Baetselier P, et al. Bone marrow-derived monocytes give rise to self-renewing and fully differentiated Kupffer cells. Nat Commun 2016;7:10321.

[15] De Visscher A, Vandeput M, Malengier-Devlies B, et al. Upregulation of Fcγ receptor IV on activated monocytes and macrophages causes nonspecific binding of the PK136 Anti-NK1.1 antibody in murine models of Toll-like receptor-induced inflammation. Scand J Immunol 2025;101:e70027.

[16] **Huang Z**, **Brodeur KE**, **Chen L**, et al. Type I interferon signature and cycling lymphocytes in macrophage activation syndrome. J Clin Invest 2023;133:e165616.

[17] Villarino AV, Kanno Y, O'Shea JJ. Mechanisms and consequences of Jak-STAT signaling in the immune system. Nat Immunol 2017;18:374–384.

[18] **Guilliams M**, **Bonnardel J**, **Haest B**, et al. Spatial proteogenomics reveals distinct and evolutionarily conserved hepatic macrophage niches. Cell 2022;185:379–396.

[19] Blasius AL, Giurisato E, Cella M, et al. Bone marrow stromal cell antigen 2 is a specific marker of type I IFN-producing cells in the naive mouse, but a promiscuous cell surface antigen following IFN stimulation. J Immunol 2006;177:3260–3265.

[20] Holmes C, Stanford WL. Concise review: stem cell antigen-1: expression, function, and enigma. Stem Cells 2007;25:1339–1347.

[21] Mahalingam S, Chaudhri G, Tan CL, et al. Transcription of the interferon γ (IFN-γ)-inducible chemokine Mig in IFN-γ-deficient mice. J Biol Chem 2001;276:7568–7574.

[22] Swiecki M, Colonna M. Unraveling the functions of plasmacytoid dendritic cells during viral infections, autoimmunity, and tolerance. Immunol Rev 2010;234:142–162.

[23] **Andrews TS**, **Nakib D**, Perciani CT, et al. Single-cell, single-nucleus, and spatial transcriptomics characterization of the immunological landscape in the healthy and PSC human liver. J Hepatol 2024;80:730–743.

[24] Matsushita H, Miyake Y, Takaki A, et al. TLR4, TLR9, and NLRP3 in biliary epithelial cells of primary sclerosing cholangitis: relationship with clinical characteristics. J Gastroenterol Hepatol 2015;30:600–608.

[25] Karrar A, Broomé U, Södergren T, et al. Biliary epithelial cell antibodies link adaptive and innate immune responses in primary sclerosing cholangitis. Gastroenterology 2007;132:1504–1514.

UNCORRECTED PROOF

[26] Canna SW, Wrobel J, Chu N, et al. Interferon-γ mediates anemia but is dispensable for fulminant Toll-like receptor 9-induced macrophage activation syndrome and hemophagocytosis in mice. Arthritis Rheum 2013;65:1764–1775.

[27] Gough DJ, Messina NL, Hii L, et al. Functional crosstalk between type I and II interferon through the regulated expression of STAT1. PLoS Biol 2010;8:e1000361.

[28] Weaver LK, Chu N, Behrens EM. Brief Report: interferon-γ–mediated immunopathology potentiated by Toll-like receptor 9 activation in a murine model of macrophage activation syndrome. Arthritis Rheumatol 2019;71:161–168.

[29] **Dooley NL**, **Chabikwa TG**, Pava Z, et al. Single cell transcriptomics shows that malaria promotes unique regulatory responses across multiple immune cell subsets. Nat Commun 2023;14:7387.

[30] Lawrence T, Natoli G. Transcriptional regulation of macrophage polarization: enabling diversity with identity. Nat Rev Immunol 2011;11:750–761.

[31] Wang N, Liang H, Zen K. Molecular mechanisms that influence the macrophage M1-M2 polarization balance. Front Immunol 2014;5:614.

[32] Coccia EM, Russo N Del, Stellacci E, et al. STAT1 activation during monocyte to macrophage maturation: role of adhesion molecules. Int Immunol 1999;11:1075–1083.

[33] Pascarella A, Bracaglia C, Caiello I, et al. Monocytes from patients with macrophage activation syndrome and secondary hemophagocytic lymphohistiocytosis are hyperresponsive to interferon gamma. Front Immunol 2021;12:663329.

[34] Verweyen E, Holzinger D, Weinhage T, et al. Synergistic signaling of TLR and IFNα/β facilitates escape of IL-18 expression from endotoxin tolerance. Am J Respir Crit Care Med 2020;201:526–539.

[35] Billiau AD, Roskams T, Van Damme-Lombaerts R, et al. Macrophage activation syndrome: characteristic findings on liver biopsy illustrating the key role of activated, IFN-γ-producing lymphocytes and IL-6- and TNF-α-producing macrophages. Blood 2005;105:1648–1651.

[36] Weaver LK, Chu N, Behrens EM. TLR9-mediated inflammation drives a Ccr2-independent peripheral monocytosis through enhanced extramedullary monocytopoiesis. Proc Natl Acad Sci USA 2016;113:10944–10949.

[37] **Tran S**, **Baba I**, Poupel L, et al. Impaired Kupffer cell self-renewal alters the liver response to lipid overload during non-alcoholic steatohepatitis. Immunity 2020;53:627–640.

[38] **Borst K**, **Frenz T**, Spanier J, et al. Type I interferon receptor signaling delays Kupffer cell replenishment during acute fulminant viral hepatitis. J Hepatol 2018;68:682–690.

[39] Blériot C, Dupuis T, Jouvion G, et al. Liver-resident macrophage necroptosis orchestrates type 1 microbicidal inflammation and type-2-mediated tissue repair during bacterial infection. Immunity 2015;42:145–158.

[40] Louwe PA, Badiola Gomez L, Webster H, et al. Recruited macrophages that colonize the post-inflammatory peritoneal niche convert into functionally divergent resident cells. Nat Commun 2021;12:1770.

[41] **Devisscher L**, **Scott CL**, Lefere S, et al. Non-alcoholic steatohepatitis induces transient changes within the liver macrophage pool. Cell Immunol 2017;322:74–83.

[42] Zigmond E, Samia-Grinberg S, Pasmanik-Chor M, et al. Infiltrating monocyte-derived macrophages and resident Kupffer cells display different ontogeny and functions in acute liver injury. J Immunol 2014;193:344–353.

[43] De Matteis A, Colucci M, Rossi M, et al. Expansion of CD4dimCD8+ T cells characterizes macrophage activation syndrome and other secondary HLH. Blood 2020;140:262–273.

[44] Chaturvedi V, Marsh RA, Zoref-Lorenz A, et al. T-cell activation profiles distinguish hemophagocytic lymphohistiocytosis and early sepsis. Blood 2021;137:2337–2346.

[45] Rood JE, Canna SW, Weaver LK, *et al.* IL-10 distinguishes a unique population of activated, effector-like CD8+ T cells in murine acute liver inflammation. J Leukoc Biol;101:1037–1044.

[46] Méndez-Sánchez N, Córdova-Gallardo J, Barranco-Fragoso B, et al. Hepatic dendritic cells in the development and progression of metabolic steatohepatitis. Front Immunol 2021;12:641240.

[47] Paracková Z, Zentsova I, Vrabcova P, et al. Aberrant tolerogenic functions and proinflammatory skew of dendritic cells in STAT1 gain-of-function patients may contribute to autoimmunity and fungal susceptibility. Clin Immunol 2023;246:109174. Q16

Keywords: CpG-induced liver inflammation; IFN-α/β; IFN-γ; scRNA-seq; MAS; PSC.

Received 13 April 2025; received in revised form 24 October 2025; accepted 29 October 2025; Available online xxx

UNCORRECTED PROOF

Journal of Hepatology, Volume ■

Supplemental information

STAT1 drives the immune landscape of murine Toll-like receptor 9-induced liver inflammation

Amber De Visscher, Jarne Beliën, Eline Bernaerts, Marte Vandeput, Bert Malengier-Devlies, Fran Prenen, Hanne Meers, Liliana Sokol, Tania Mitera, Nele Berghmans, Seray Anak, Olivier Govaere, Philippe Van den Steen, Jochen Lamote, Niels Vandamme, Anna Bujko, Charlotte L. Scott, Carine H. Wouters, and Patrick Matthys

STAT1 drives the immune landscape of murine Toll-like receptor 9-induced liver inflammation

Amber De Visscher, Jarne Beliën, Eline Bernaerts, Marte Vandeput, Bert Malengier-Devlies, Fran Prenen, Hanne Meers, Liliana Sokol, Tania Mitera, Nele Berghmans, Seray Anak, Olivier Govaere, Philippe Van den Steen, Jochen Lamote, Niels Vandamme, Anna Bujko, Charlotte L. Scott, Carine H. Wouters, Patrick Matthys

Table of contents

Methods…………………………………………………………………………………2

Supplementary figures………………………………………………………………...4

Supplementary tables………………………………………………………………..18

Supplementary references…………………………………………………………..24

Methods

Mice

WT C57BL/6 mice were purchased from Janvier Laboratories or bred at the Animal Centre of the Rega Institute, KU Leuven. *Ifng*$^{-/-}$ C57BL/6 mice were purchased from The Jackson Laboratory, and *Stat1*$^{-/-}$ C57BL/6 mice were kindly provided by Dr. Andrew Brown and Dr. Andy Wullaert (VIB-University of Ghent), and further bred at the Animal Centre of the Rega Institute, KU Leuven.

Scoring of liver inflammation

Liver sections were blindly score and the following hepatic inflammatory scores were defined: general liver inflammation (0; no inflammation, 1; sporadic inflammation, 2; mild inflammation, 3; moderate inflammation and 4; severe inflammation), lobular inflammation (average number of inflammatory foci/mm^2 counted in 10x 1mm^2 squares and periportal inflammation (number of inflamed hepatic portal veins out of 10).

Protein extraction and western blot

For protein extraction, tissue or cells were lysed with RIPA buffer (TCI Chemicals) containing protease (Roche) and phosphatase inhibitor cocktails (Sigma-Aldrich). Of each sample, 25 µg of protein was resuspended in PierceŁ reducing loading buffer (Thermo Fisher Scientific) and incubated for 10 min at 80°C. Samples were separated in 4-12% Tris-Glycine gels (precast Novex Tris-Glycine gels, ThermoFisher Scientific). Proteins in gels were transferred onto PVDF membranes using the Trans-Blot Turbo Transfer system with associated materials and protocols.

Single-cell transcriptomics

Preprocessing of sequencing data and quality control

The Cell Ranger toolkit (v6.0.0) was used for demultiplexing, alignment to the GRCm38.99 mouse reference genome, and decoding of the CITE-seq barcodes. Before integration, the four samples went separately through the QC pipeline in R (v4.2.2). Genes present in fewer than five cells were removed, and only high-quality cells, defined as cells expressing at least 200 genes, were retained. Additionally, thresholds for the minimum and maximum unique molecular identifier (UMI) content per cell and the maximum mitochondrial RNA content were determined automatically using the Scater package with default parameters (v1.26.1). Cells that did not meet these criteria were filtered out. The corrected gene count matrices, antibody-derived tags (ADT) count matrices, and relevant metadata were used to create a single cell experiment (sce) object with the SingleCellExperiment package (v1.20.1). Doublets were identified via scDblFinder (v1.12.0) with default parameters and removed from the sce objects, which were then converted into Seurat objects with the Seurat package (v4.4.0) for further analysis. The count matrices of each Seurat object were normalized and scaled using SCTransform (v0.4.1) with default parameters, and mitochondrial content was regressed out.

Data integration, batch effect removal, and cluster annotation

To avoid sample-specific clusters, we integrated the gene count matrices of the individual samples using Seurat integration (v4.4.0) with default parameters, treating each sample as a batch. This process was repeated for the ADT count matrix. For downstream subclustering, the desired cell cluster(s) were selected and put into a new Seurat object, and the above-mentioned procedure was repeated. To cluster the cells, we ran RunPCA(), FindNeighbours(), and FindClusters(). The clusters were visualized on a Uniform Manifold Approximation and Projection (UMAP) plot. Optimal cluster resolution was determined with guidance from the clustree (v0.5.1) package. Differential gene

expression (DGE) analysis and differential protein expression (DPE) were performed using FindAllmarkers() with default parameters to identify the most differentially expressed genes and proteins in each cluster. To annotate the clusters, we checked the expression of canonical marker genes and proteins (**Supplementary Figure S1A-B**). Furthermore, we used SingleR (v2.0.0) for automated annotation based on ImmGen reference, which consists of microarray profiles of pure mouse immune cells from the Celldex package (v1.8.0), to sanity check our manual annotations.

Correction for the enrichment of F4/80+ cells

To show the correct percentages without enrichment in **Figure 2C** and **Figure 4B**, we diminished the total counts of the F4/80^{+} cells ('Transitioning monocytes' and 'Macrophages') with 1,4% (1/70). Based on these corrected counts, the corresponding percentages were calculated.

Downstream analyses

Differential composition analysis was performed using the mixed-effects association testing for single cells (MASC) package (0.1.0-alpha). The Odds ratio (OR) was calculated using a general linearized mixed-effects model with condition as the contrast of interest, cell cycle phase as a random-effect covariate, and the number of genes detected as a fixed-effect covariate. To perform pseudotime trajectory analysis on the myeloid compartment, we used Slingshot (v2.6.0). Since non-monocyte/Mφ clusters ("Migratory dendritic cells", "Type 1 conventional dendritic cells", "Type 2 conventional dendritic cells", and "Plasmacytoid dendritic cells") might interfere with the trajectories, we subsetted our data to only include monocyte/Mφ clusters for the trajectory analysis. Additionally, we carried out DGE analysis along the inferred trajectories with fitGAM(), assoRes(), and startVsEndTest() from the TradeSeq package (v1.12.0). The module score for the 'CD38^{+}HLA-DR^{+} cycling lymphocyte' signature of patients with macrophage activation syndrome (MAS), a cytokine storm syndrome developing in the context of rheumatic disorders, from Huang *et al.*[16] was calculated with AddModuleScore() using the top 10 enriched genes: *Cenpf*, *Gapdh*, *Gzma*, *Hmgb2*, *Hmgn2*, *Mki67*, *Pclaf*, *Stmn1*, *Tuba1b,* and *Tyms*.

Statistics

Differential gene/protein expression data were tested for statistical significance using a Wilcoxon rank sum test with Bonferroni correction. Proteins were highlighted if $P<0.05$ with a minimal increase (red) or decrease (blue) of log2(FC)=0.322 (**Fig. 4G**). Differential expression of module scores across clusters was demonstrated with a Kruskal-Wallis test followed by a Dunn's test for multiple comparisons with Benjamini-Hochberg correction. For GSEA, the wilcoxauc function (Presto) was used to calculate the Area Under the (receiver operator) Curve (AUC) values as an alternative to adjusted p-values. For the DGE analyses across trajectories, statistical significance was evaluated using a Wald test with Benjamini-Hochberg correction for multiple testing. To determine significant changes in the composition of the ORs as calculated by MASC, a general linearized mixed-effects model with Bonferroni correction was used.

Publicly available human datasets

The GSEA of the RNA-seq data set of PBMCs from MAS and HC patients was republished with permission from the authors (Fig. 1C of Huang *et al.*[1]). To evaluate the expression of type I and II IFN signatures in PSC patients from the dataset of Andrews *et al.*[2], we plotted the expression of established type I[3] and II[4] IFN gene sets using their online visualization tool (https://macparlandlab.shinyapps.io/shiny_sc/).

Supplementary figures

Fig. S1

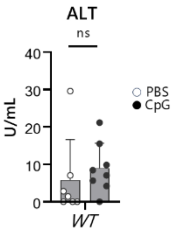

Fig. S1. TLR9 triggering represents a model for liver inflammation without hepatotoxicity. ALT levels in serum. Bars (mean) and error bars (standard deviation). ns P>0.05 [Mann-Whitney U-test].

Fig. S2

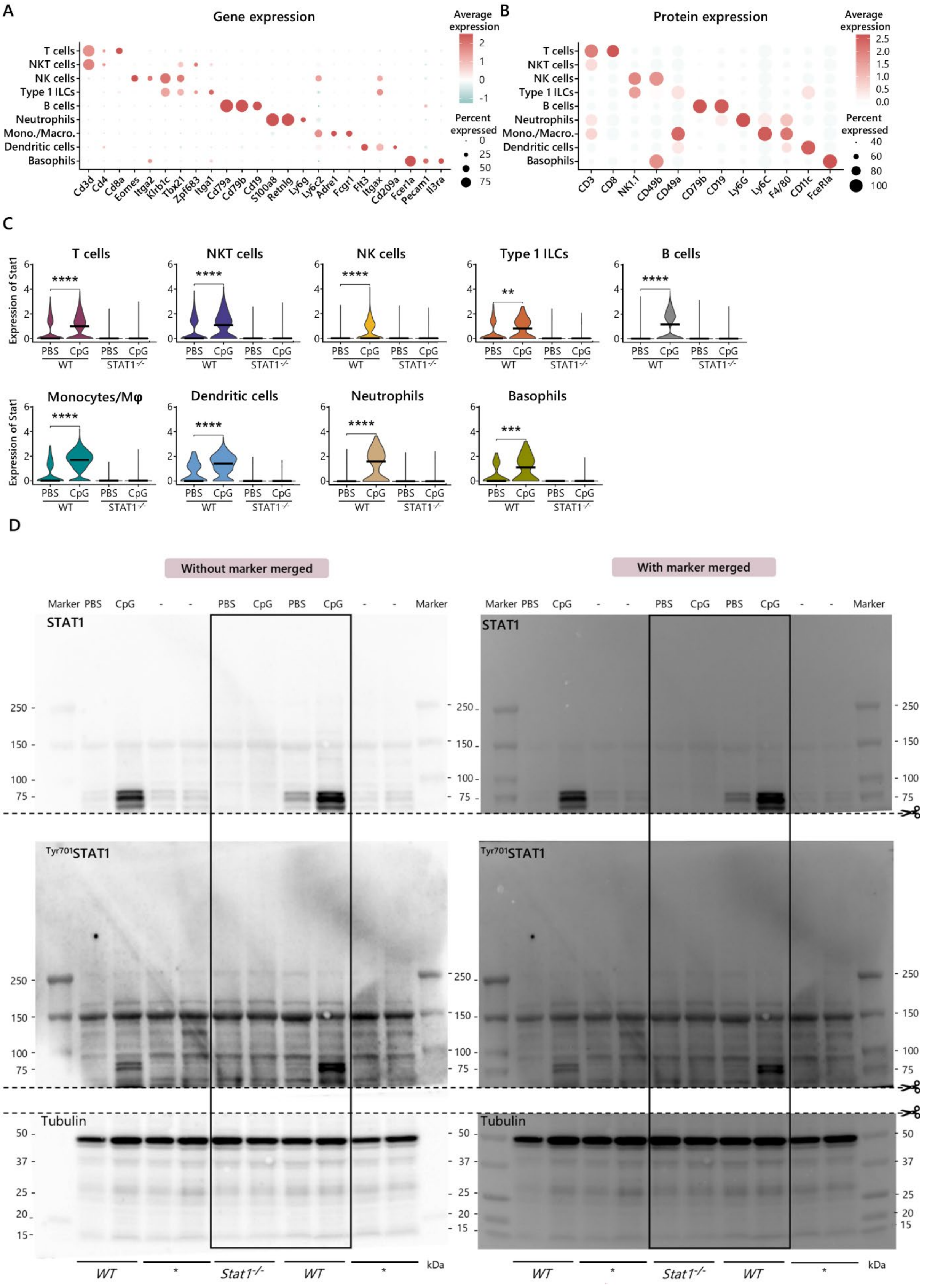

Fig. S2. Validation of the general cluster annotation and visualization of *Stat1* expression in the different clusters. Experimental setup (**Fig. 2A**). Expression of canonical (A) genes and (B)

protein markers of all clusters of the integrated dataset. (C) *Stat1* expression. Colors (clusters **Fig. 2B**). $^{**}P<0.01$, $^{***}P<0.001$ and $^{****}P<0.0001$ [Kruskal-Wallis test followed by a Dunn's test for multiple comparisons with Benjamini-Hochberg correction (C)]. (D) Representative uncropped western blots of pSTAT1, STAT1, and Tubulin (un)merged with the marker (ladder). The results shown in **Fig. 2G** are boxed. *Asterisks refer to lanes not relevant for the data presented in this manuscript. Blots without marker merged were used for formal analysis with ImageJ. Values obtained for STAT and pSTAT were first normalized against Tubulin. Outliers in pSTAT/STAT ratio were identified by the ROUT method (Q = 1%) and excluded from the analysis. Remaining original blots of the other mice (in total 8 WT and 4 STAT1$^{-/-}$) are shown in **Fig. S13**.

Fig. S3

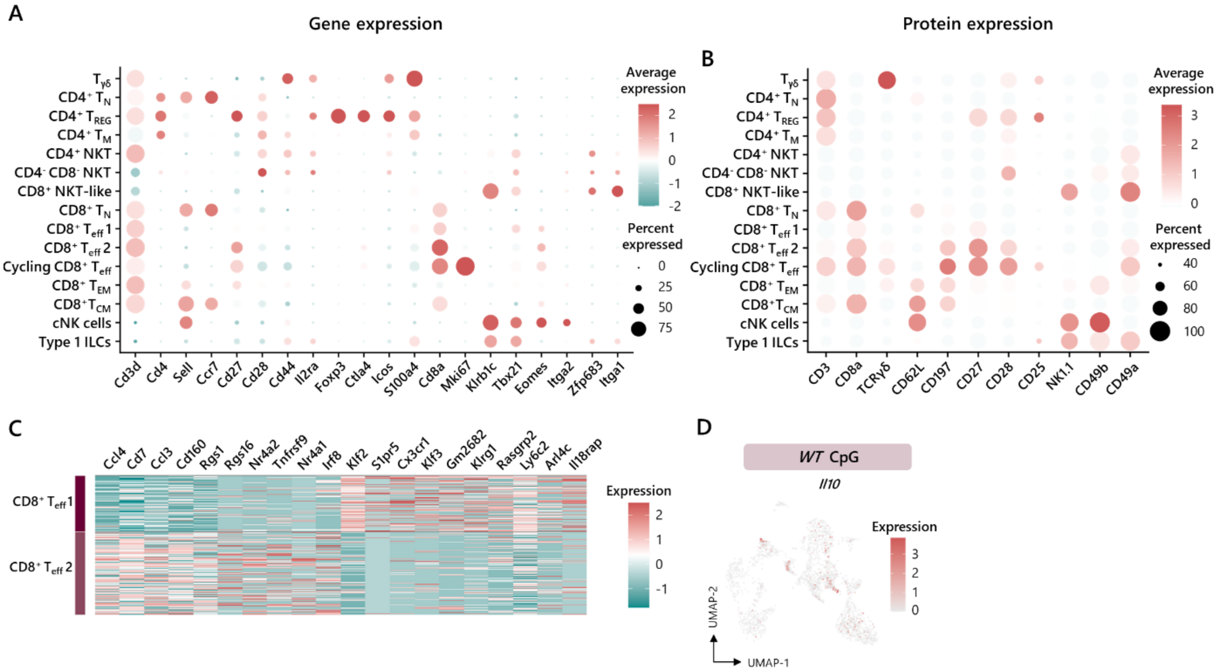

Fig. S3. Validation of the lymphoid cluster annotation and DGE of the two different TLR9-associated CD8$^+$ effector T cells. Experimental setup (**Fig. 2A**). Expression of canonical (A) genes and (B) protein markers of all clusters of the lymphoid subclustering of the integrated dataset. (C) Top ten differentially expressed genes of CD8$^+$ $T_{eff}1$ versus CD8$^+$ $T_{eff}2$ of the lymphoid subclustering in *WT* CpG. Colors (clusters Fig. 2B) and color scale (average log-normalized expression). (D) *Il10* expression.

Fig. S4

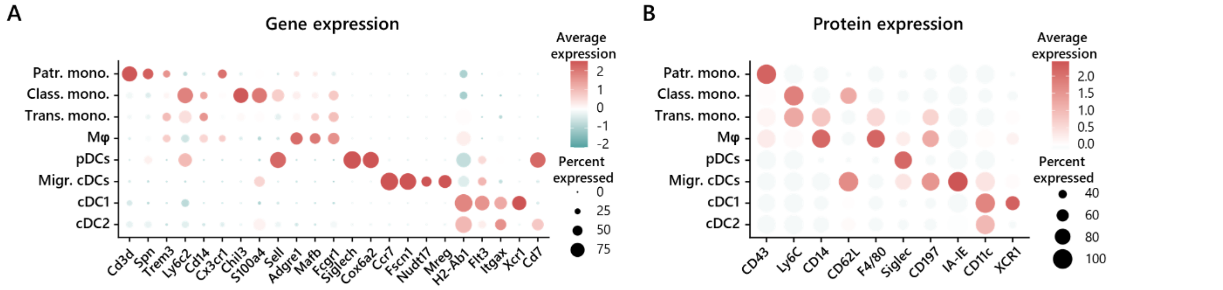

Fig. S4. Validation of the myeloid cluster annotation. Experimental setup (**Fig. 2A**). Expression of canonical (A) genes and (B) protein markers of all clusters of the myeloid subclustering of the

integrated dataset. Dot size (percentage of expressing cells) and color scale (average log-normalized expression).

Fig. S5

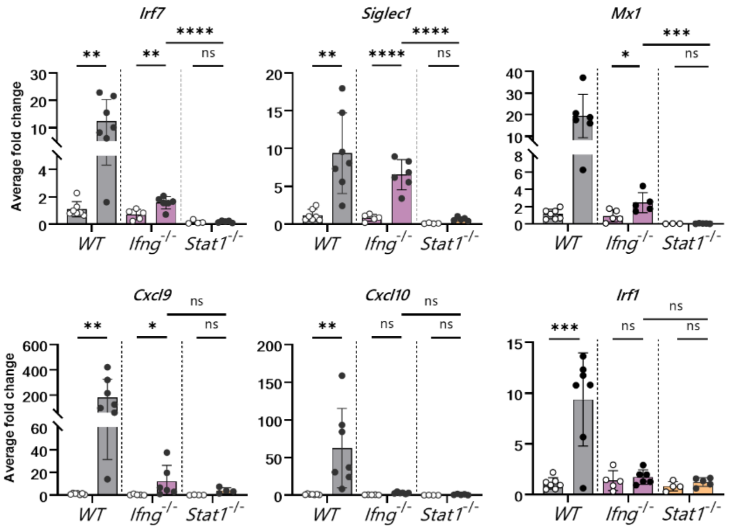

Fig. S5. Induction of type I and type II IFN-stimulated genes during TLR9-mediated liver inflammation. Experimental setup (**Fig. 1A**). Fold change in expression of interferon-stimulated genes in total liver lysate as measured by RT-QPCR. Bars (mean) and error bars (standard deviation). ns *P*>0.05, **P*<0.05, ***P*<0.01, *****P*<0.0001 [Student's t-test (*WT* in *Siglec1*, *Mx1*, Cxcl9, iRf1), Mann-Whitney U-test (*WT* in *Irf7*, *Cxcl10*), Šídák's multiple-comparisons test (*Ifng*$^{-/-}$, *Stat1*$^{-/-}$ in *Irf7*, *Siglec1*, *Mx1*, *Cxcl10*, *Irf1*), Dunn's multiple-comparisons test (*Ifng*$^{-/-}$, *Stat1*$^{-/-}$ in *Cxcl9*)].

Fig. S6

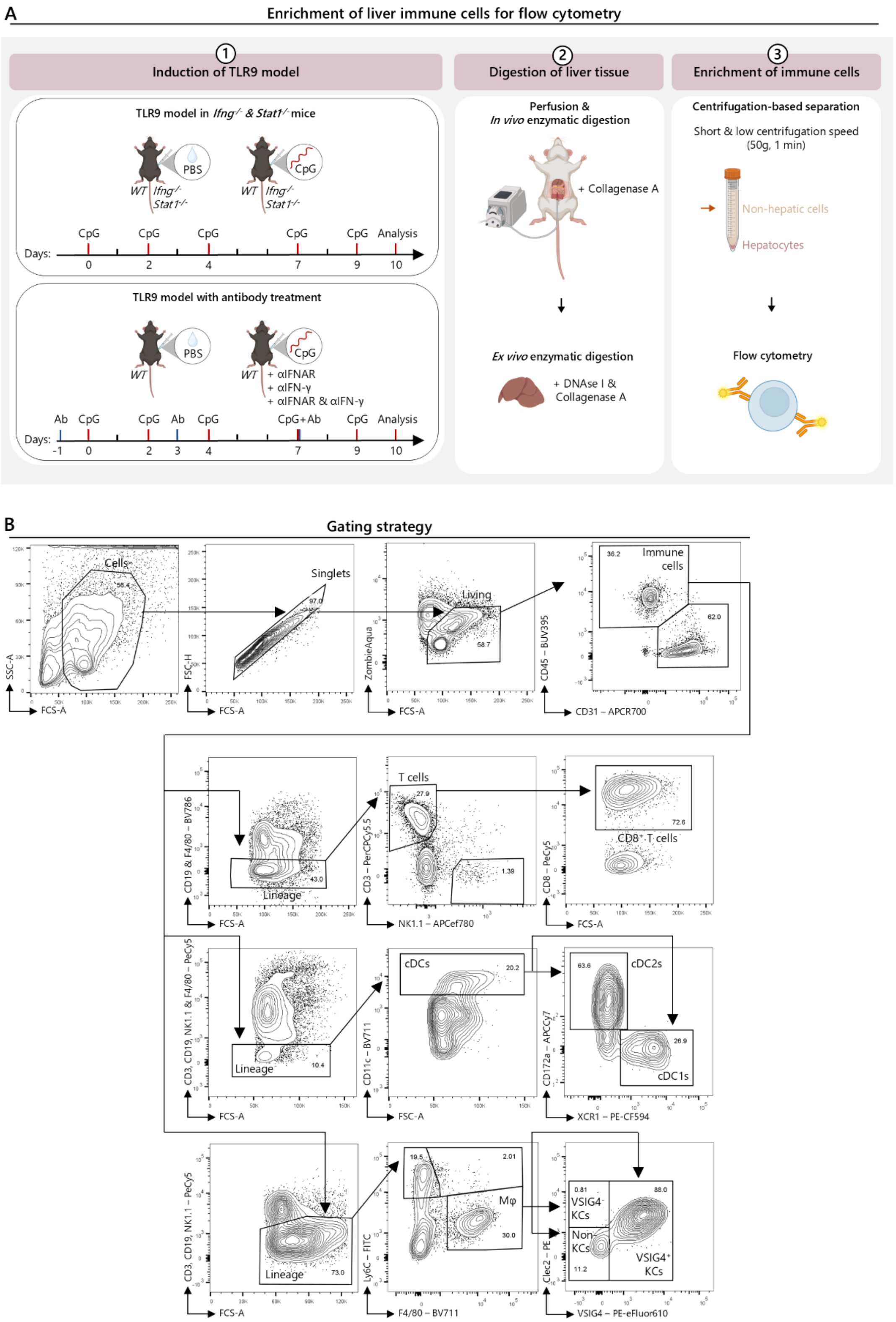

Fig. S6. Overview of the experimental setup for flow cytometry and gating strategies. (A) Experimental setup. (B) Representative gating strategy of the TLR9-associated populations.

Fig. S7

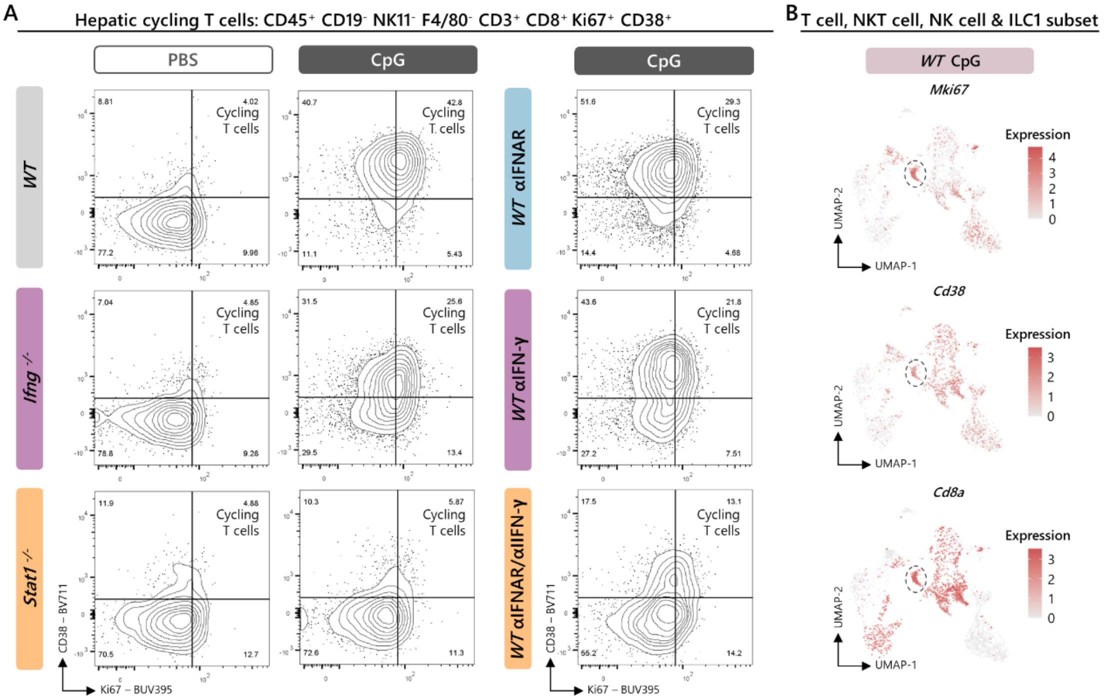

Suppl. Figure S7. Comparison of cycling T cells identified by flow cytometry *versus* CITE-seq. Experimental setup and gating strategy (**Fig. S6**). (A) Representative flow cytometry plots of cycling T cells (CD45$^+$CD19$^-$NK1.1$^-$F4/80$^-$CD3$^+$CD8$^+$Ki67$^+$CD38$^+$ cells). (B) Lymphoid subclustering of *WT* CpG. Color scale (average log-normalized gene expression).

Fig. S8

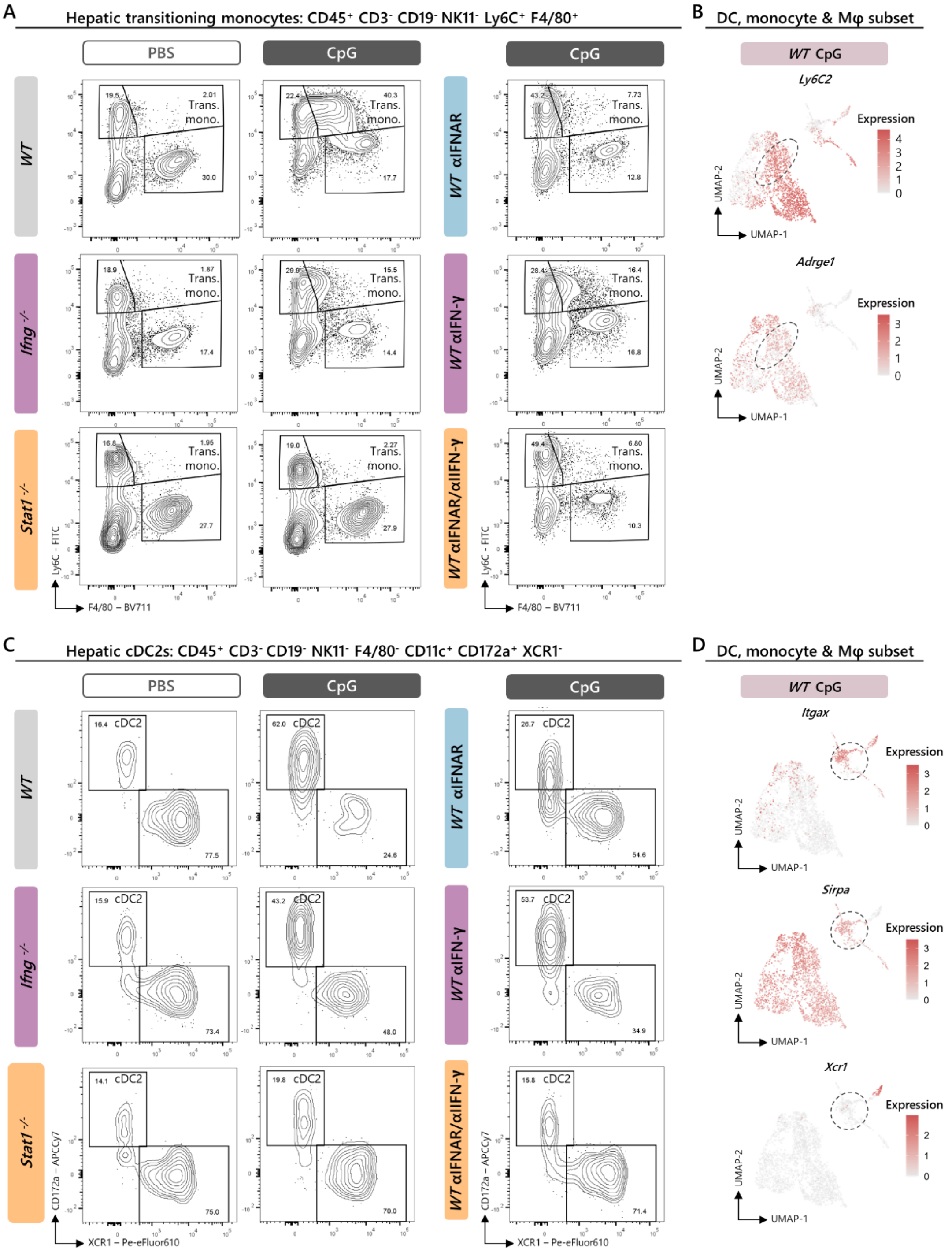

Fig. S8. Comparison of transitioning monocytes and type 2 conventional dendritic cells identified by flow cytometry *versus* CITE-seq. Experimental setup and gating strategy (**Fig. S6**). Representative flow cytometry plots of (A) transitioning monocytes ($CD45^{+}CD3^{-}CD19^{-}NK1.1^{-}$ $Ly6C^{+}F4/80^{+}$) and, (C) cDC2 ($CD45^{+}CD3^{-}CD19^{-}NK1.1^{-}Ly6C^{-}F4/80^{-}CD11c^{+}CD172a^{+}$ $XCR1^{-}$). (B) Lymphoid subclustering and (D) myeloid subclustering of *WT* CpG. Color scale (average log-normalized gene expression).

Fig. S9

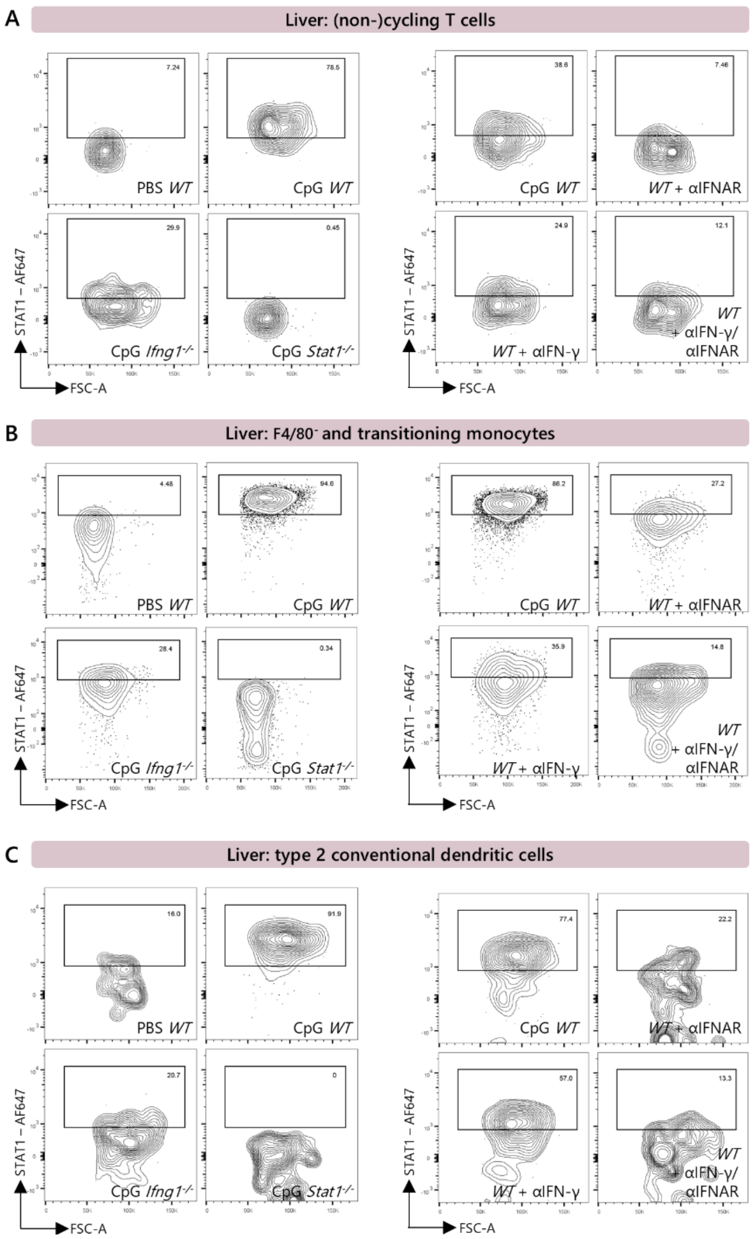

Fig. S9. Representative plots of STAT1 expression in T cells, monocytes, and DCs. Experimental setup and gating strategy (**Fig. S6A**). (A) Representative flow cytometry plots of STAT1 expression in non-cycling (*WT* PBS, *Stat1*$^{-/-}$ CpG) and cycling T cells (*WT* CpG, *Ifng*$^{-/-}$ CpG, *WT* + anti-IFNAR, *WT* + anti-IFN-γ, *WT* + anti-IFNAR and + anti-IFN-γ). (B) Representative flow cytometry plots of STAT1 expression in F4/80^{-} (*WT* PBS, *Stat1*$^{-/-}$ CpG, *WT*) and transitioning monocytes (*WT* CpG, *Ifng*$^{-/-}$ CpG, *WT* + anti-IFNAR, *WT* + anti-IFN-γ, *WT* + anti-IFNAR and + anti-IFN-γ). (C) Representative flow cytometry plots of STAT1 expression in cDC2.

Fig. S10

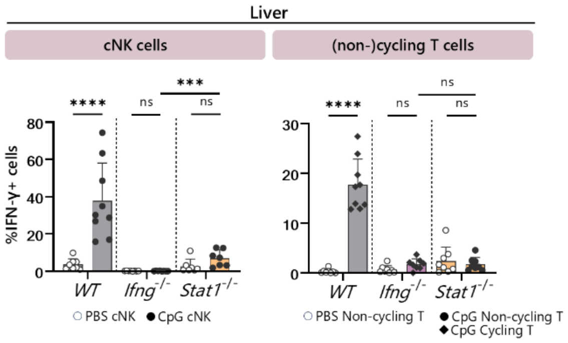

Fig. S10. Production of type II IFN by cycling T cells and cNK cells during TLR9-mediated liver inflammation. Experimental setup and gating strategy (**Fig. S6A**). (A) Percentage of IFN-γ^{+} cNK cells, and (non-)cycling T cells. Bars (mean) and error bars (standard deviation). ns, p>0.05, ***p<0.001 ****p<0.0001 [Student's t-test (*WT* in cNK cells), Mann-Whitney U-test (*WT* in T cells), Dunn's multiple-comparisons test (*Ifng*$^{-/-}$, *Stat1*$^{-/-}$ in cNK cells, T cells].

Fig. S11

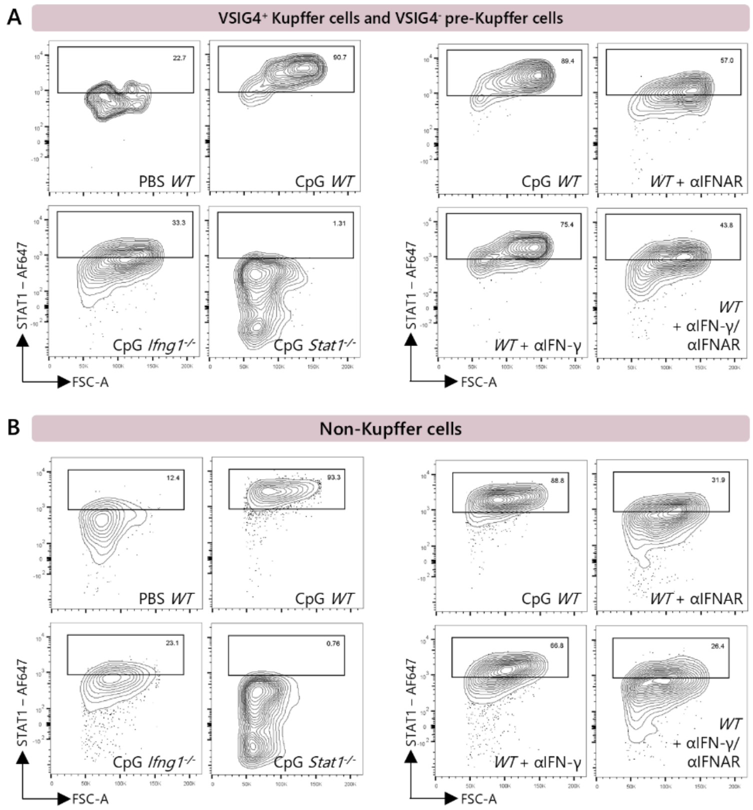

Fig. S11. Representative plots of STAT1 expression in macrophage populations. Experimental setup and gating strategy (**Fig. S6A**). (A) Representative plots of STAT1 expression in VSIG4^{+} Kupffer cells (*WT* PBS, *Stat1*$^{-/-}$ CpG, *WT* + anti-IFNAR, *WT* + anti-IFN- γ, *WT* + anti-IFNAR and + anti-IFN-γ) and VSIG4^{-} pre-Kupffer cells (*WT* CpG, *Ifng*$^{-/-}$ CpG, *Stat1*$^{-/-}$ CpG, *WT* + anti-IFNAR, *WT* + anti-IFN-γ, *WT* + anti-IFNAR and + anti-IFN-γ). (B) Representative plots of STAT1 expression in non-Kupffer cells.

Fig. S12

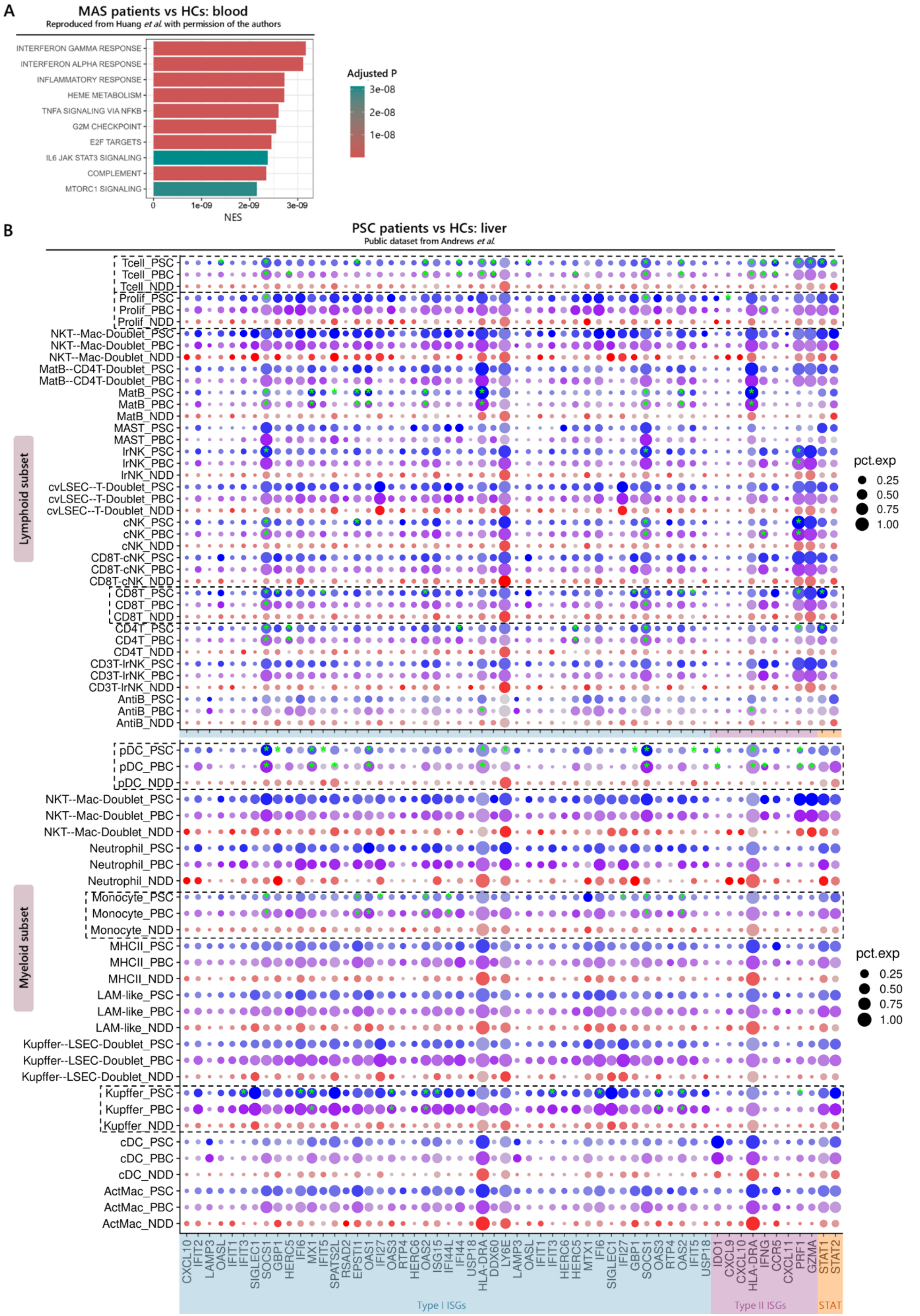

Fig. S12. STAT1 expression and a type I and II interferon signature in blood and liver of human

TLR9-implicated pathologies. (A) GSEA of patients with macrophage activation syndrome (MAS) versus healthy controls (HCs) reproduced from the publicly available dataset from Huang *et al.*[1] ($P_{corrected}$ <0.05). (B) Expression of type I and II ISGs in patients with primary sclerosing cholangitis (PSC, blue), patients with primary biliary cholangitis (PBC, purple), and HCs (NDD; neurologically deceased healthy donor, red) from the publicly available dataset from Andrews *et al.*[3] Populations discussed in the results are boxed. Stars indicate a significant difference (FDR<5%). Prolif; Proliferating cell, NKT; Natural killer T cell, Mac; Macrophage, MatB; Mature B cell, lrNK; Liver-resident NK cell, cNK; Conventional natural killer cell, antiB; Antibody-secreting B cell, pDC; Plasmacytoid dendritic cell, LAM; Lipid-associated macrophage, (cv)LSEC; (central venous) Sinusoidal endothelial cell, cDC; Conventional dendritic cell, ActMac; Activated macrophage.

Fig. S13 (page 15-18)

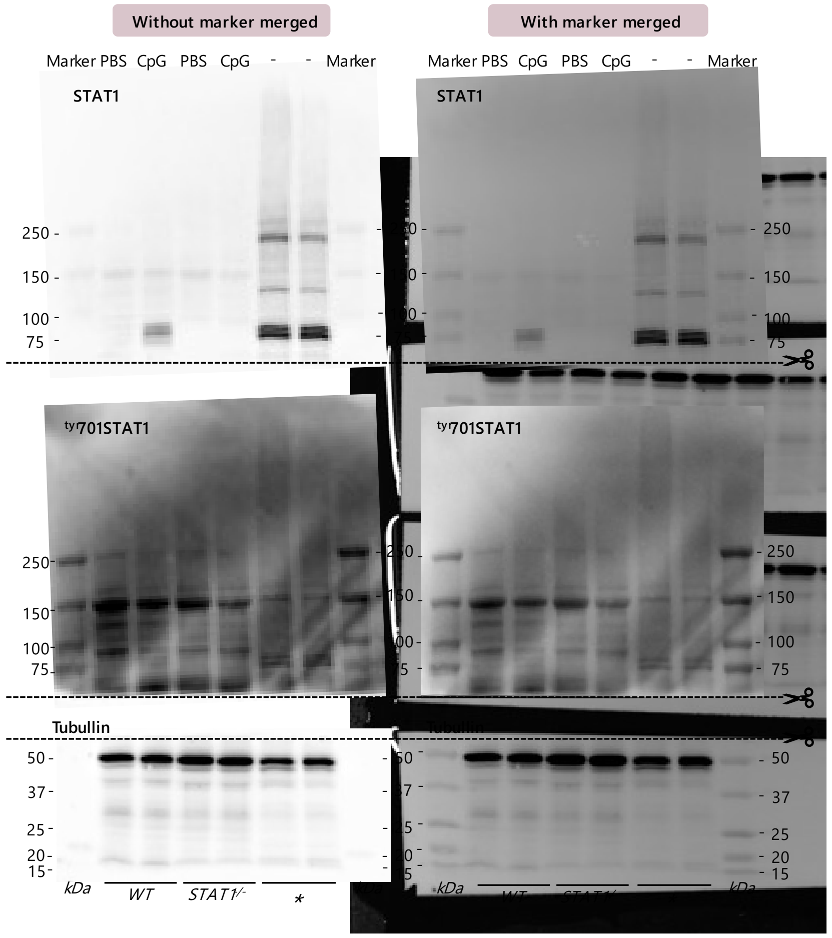

Fig. S13 (continuation)

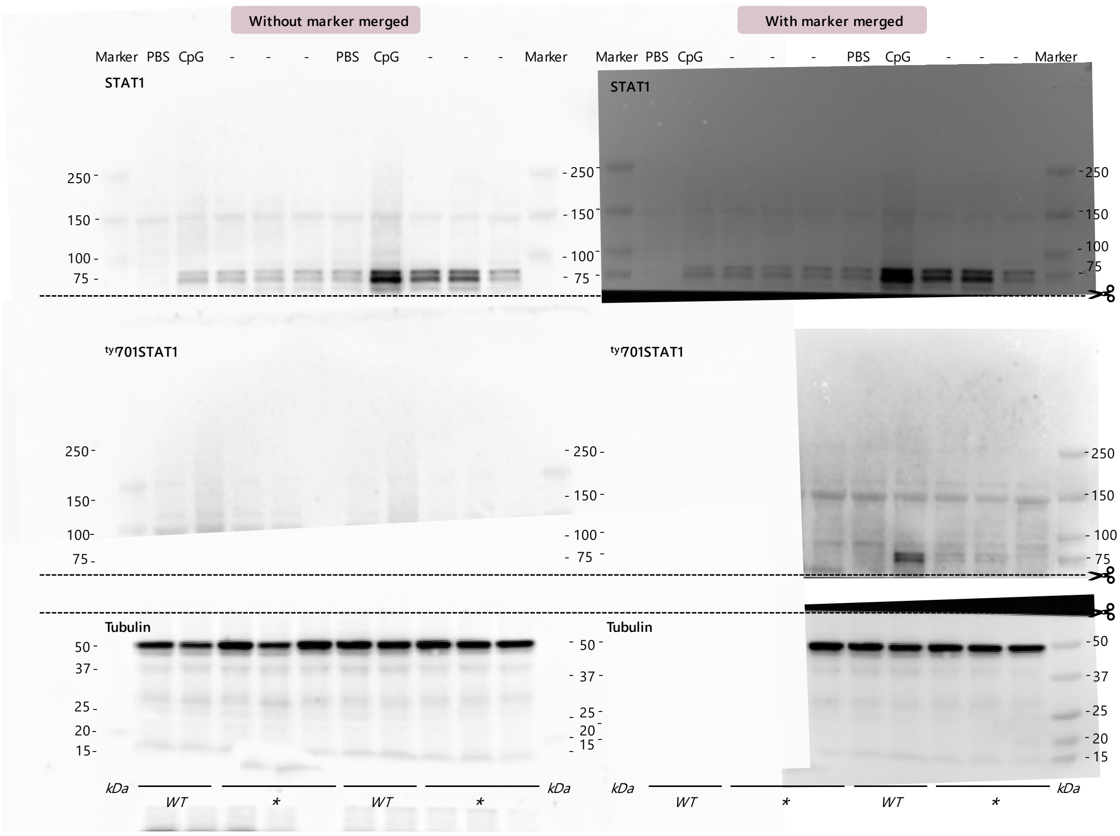

Fig. S13 (continuation)

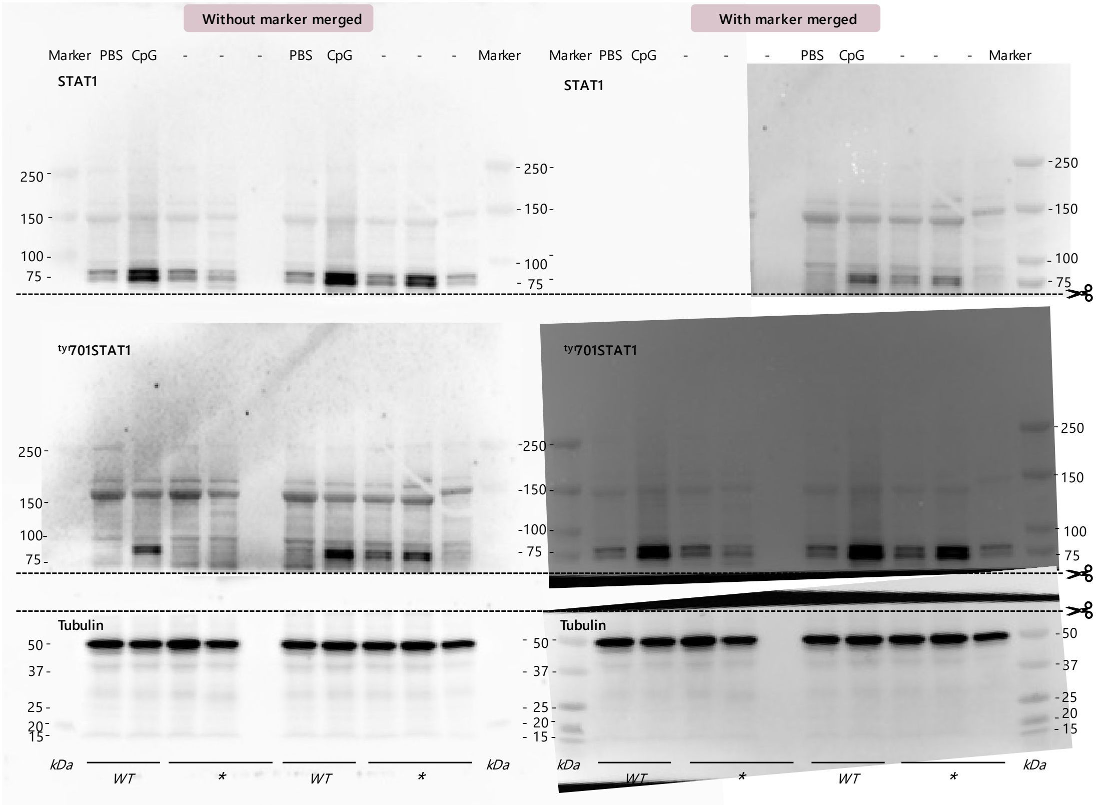

Fig. S13 (continuation)

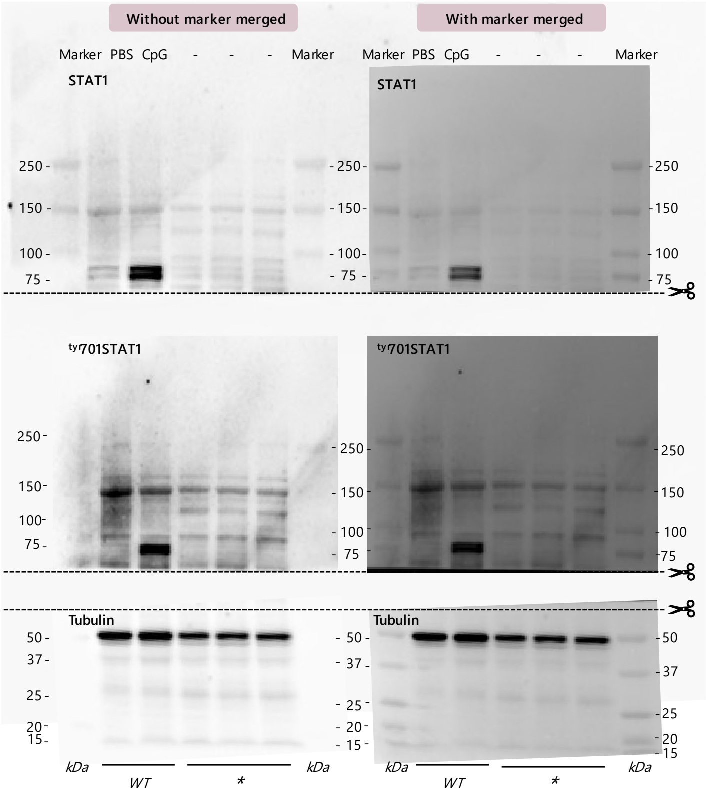

Fig. S13 (continuation)

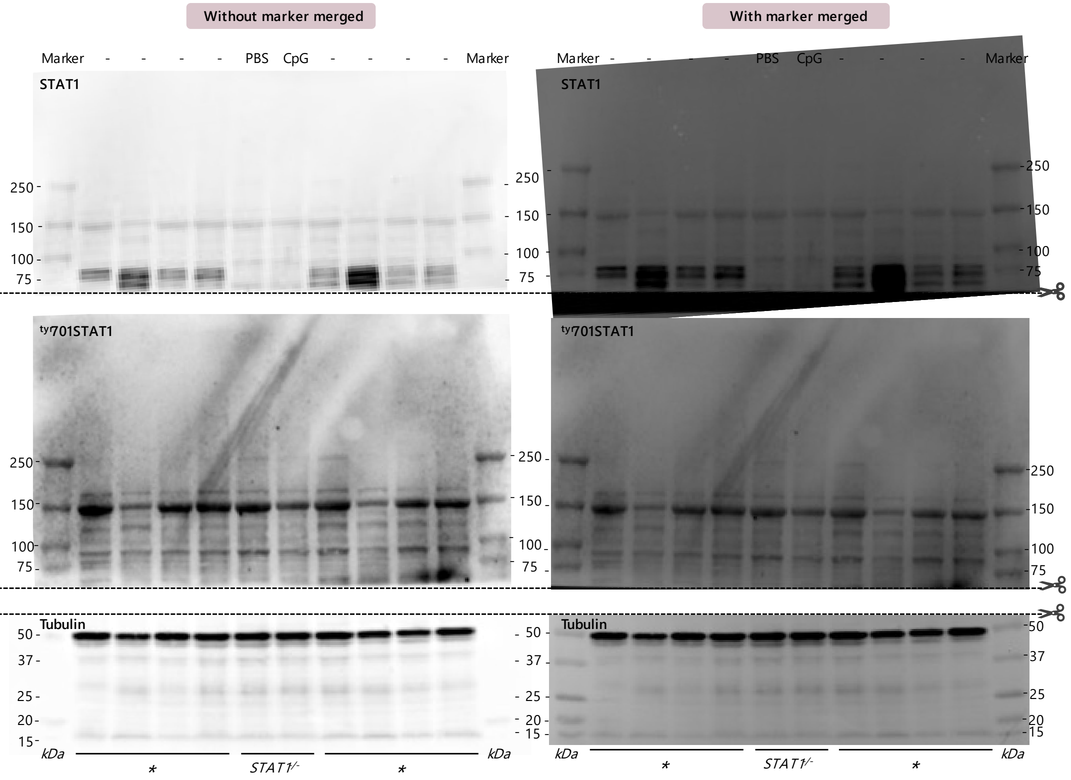

Fig. S 13 (continuation)

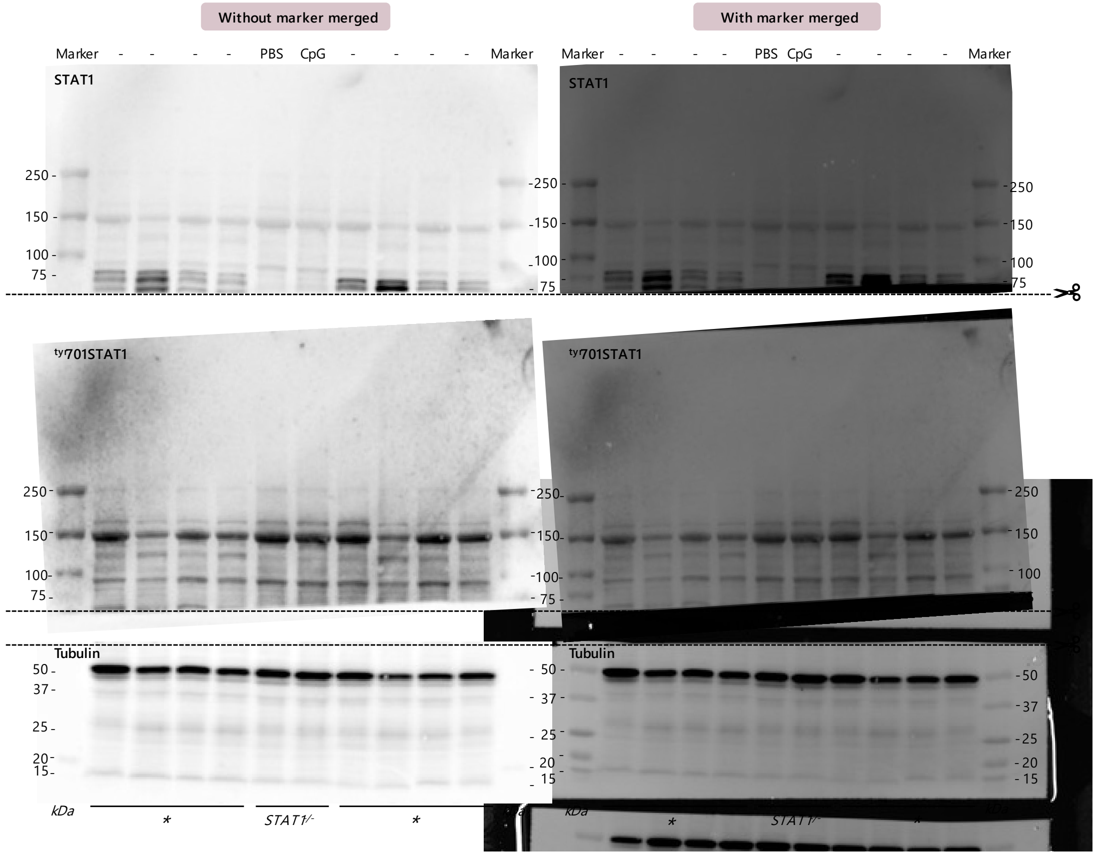

Fig. S13. Remaining original western blots of pSTAT1, STAT1, and Tubulin (un)merged with the marker/ladder (the first western blot is shown in Suppl. Fig. 2 D). Asterisks refer to lanes not relevant for the data presented in this manuscript. Blots without marker merged were used for formal analysis. Blots were analyzed with ImageJ. Values obtained for STAT and pSTAT were first normalized against Tubulin. Outliers in the pSTAT/STAT ratio were identified by the ROUT method (Q = 1%) and excluded from the analysis. The pSTAT/STAT ratio values of the 8+8 wildtype (WT) mice (PBS and CpG) and the 4+4 STAT1 knockout (STAT1$^{-/-}$) mice (PBS and CpG) are shown in **Fig. 2G**.

Supplementary tables

Table S1. RT-qPCR primers

Antigen	Exon location	Assay ID	Fluorescent dye	Company
Cxcl9	Exon 2-3	Mm.PT.58.57267	FAM™	IDT
Cxcl10	Exon 1-2	Mm.PT.58.43575827	FAM™	IDT
Ifit1	Exon 1-2	Mm.PT.58.32674307	FAM™	IDT
Ifng	Exon 1-2	Mm.PT.58.41769240	FAM™	IDT
Il6	Exon 4-5	Mm.PT.58.10005566	FAM™	IDT
Il12b	Exon 2-3	Mm.PT.58.12409997	FAM™	IDT

Il18	Exon 4-5	Mm.PT.58.42776691	FAM™	IDT
Irf7	Exon 2-3	Mm.PT.58.32394021.g	FAM™	IDT
Isg15	Exon 2-2	Mm.PT.58.41476392.g	FAM™	IDT
Siglec1	Exon 17-18	Mm.PT.58.8446763	FAM™	IDT
Tnfa	Exon 2-4	Mm.PT.58.12575861	FAM™	IDT

Table S2. Fluorochrome-labelled antibodies

Antigen	Clone	Fluorochrome	Company
FACS of CD45+ with enriched F4/80+ cells for CITE-seq			
CD45	30-F11	PerCP-Cy5.5	BioLegend
CD49a	Ha31/8	PE	BD Biosciences
CD3	145-2C11	BV786	BD Biosciences
CD19	6D5	BV785	BioLegend
F4/80	Bm8	BV711	BioLegend
Flow cytometric validation of TLR9-associated populations			
CD3	145-2C11	PerCP-Cy5.5, PE-Cy5, PE-Cy7	Invitrogen, BioLegend
CD8	53-6.7	PE-Cy5, FITC, BV786	Invitrogen, BioLegend
CD11c	N418	BV711, PerCP-Cy5.5	BioLegend
CD19	6D5	PE-Cy5	BioLegend
CD38	90/CD38	BV711	BD Biosciences
CD45	30-F11	BUV395	Invitrogen
CD172a	P84	APC-Cy7	BioLegend
CD317	q27	BV605	BioLegend
CLEC2	17D9/CLEC-2	PE	BioLegend
F4/80	BM8	PE, PE-Cy5	BioLegend
IFN-γ	XMG1.2	PE	BioLegend
Ki67	B56	BUV395	BD Biosciences
Ly6C	HK1.4	FITC	BioLegend
NK1.1	PK136	PE-Cy5, APC-eF780	BioLegend, Invitrogen
STAT1	1/STAT1	AF647	BD Biosciences
VSIG4	NLA14	PE-Dazzle594	Invitrogen
XCR1	ZET	PE-Dazzle594	BioLegend

Table S3. Oligo-labelled antibodies

Antigen	Clone	Identifier	Company
Allophycocyanin	APC003	TotalSeq-C0987	BioLegend
Biotin	1D4-C5	TotalSeq-C0436	BioLegend
CD1d	1B1	TotalSeq-C0851	BioLegend
CD2	RM2-5	TotalSeq-C0892	BioLegend
CD3	17A2	TotalSeq-C0182	BioLegend
CD5	53-7.3	TotalSeq-C0111	BioLegend
CD8a	53-6.7	TotalSeq-C0002	BioLegend
CD8b	YTS156.7.7	TotalSeq-C0230	BioLegend
CD9	MZ3	TotalSeq-C0813	BioLegend
CD11a	M17/4	TotalSeq-C0595	BioLegend
CD11b	M1/70	TotalSeq-C0014	BioLegend
CD11c	N418	TotalSeq-C0106	BioLegend
CD14	Sa14-2	TotalSeq-C0424	BioLegend
CD15	MC-480	TotalSeq-C0076	BioLegend
CD16/32	93	TotalSeq-C0109	BioLegend
CD19	6D5	TotalSeq-C0093	BioLegend

CD20	SA275A11	TotalSeq-C0192	BioLegend
CD21,CD35	7,00E+09	TotalSeq-C0107	BioLegend
CD22	OX-97	TotalSeq-C0827	BioLegend
CD23	B3B4	TotalSeq-C0108	BioLegend
CD24	M1/69	TotalSeq-C0212	BioLegend
CD25	PC61	TotalSeq-C0097	BioLegend
CD27	LG.3A10	TotalSeq-C0191	BioLegend
CD28	37.51	TotalSeq-C0204	BioLegend
CD29	HMβ1-1	TotalSeq-C0570	BioLegend
CD30	mCD30.1	TotalSeq-C1062	BioLegend
CD31	390	TotalSeq-C0904	BioLegend
CD34	SA376A4	TotalSeq-C0823	BioLegend
CD34	HM34	TotalSeq-C0857	BioLegend
CD38	90	TotalSeq-C0557	BioLegend
CD39	Duha59	TotalSeq-C0834	BioLegend
CD40	mrt/23	TotalSeq-C0903	BioLegend
CD41	MWReg30	TotalSeq-C0443	BioLegend
CD43	S11	TotalSeq-C0110	BioLegend
CD44	IM7	TotalSeq-C0073	BioLegend
CD45	30-F11	TotalSeq-C0096	BioLegend
CD45.1	A20	TotalSeq-C0178	BioLegend
CD45.2	104	TotalSeq-C0157	BioLegend
CD45R/B220	RA3-6B2	TotalSeq-C0103	BioLegend
CD45RB	C363-16A	TotalSeq-C1063	BioLegend
CD48	HM48-1	TotalSeq-C0429	BioLegend
CD49a	HMα1	TotalSeq-C0850	BioLegend
CD49b	HMα2	TotalSeq-C0421	BioLegend
CD49d	R1-2	TotalSeq-C0078	BioLegend
CD49f	GoH3	TotalSeq-C0070	BioLegend
CD54	YN1/1.7.4	TotalSeq-C0074	BioLegend
CD55	RIKO-3	TotalSeq-C0558	BioLegend
CD61	2C9.G2 (HMβ3-1)	TotalSeq-C0910	BioLegend
CD62L	MEL-14	TotalSeq-C0112	BioLegend
CD62P	RMP-1	TotalSeq-C0229	BioLegend
CD63	NVG-2	TotalSeq-C0559	BioLegend
CD64	X54-5/7.1	TotalSeq-C0202	BioLegend
CD68	FA-11	TotalSeq-C0560	BioLegend
CD69	H1.2F3	TotalSeq-C0197	BioLegend
CD71	RI7217	TotalSeq-C0441	BioLegend
CD73	TY/11.8	TotalSeq-C0077	BioLegend
CD79b	HM79-12	TotalSeq-C0561	BioLegend
CD80	16-10A1	TotalSeq-C0849	BioLegend
CD81	Eat-2	TotalSeq-C1064	BioLegend
CD83	Michel-19	TotalSeq-C0562	BioLegend
CD85k	H1.1	TotalSeq-C1007	BioLegend
CD86	GL-1	TotalSeq-C0200	BioLegend
CD88	20/70	TotalSeq-C1042	BioLegend
CD90.2	30-H12	TotalSeq-C0075	BioLegend
CD90/CD90.1	OX-7	TotalSeq-C0380	BioLegend
CD93	AA4.1	TotalSeq-C0113	BioLegend
CD94	18d3	TotalSeq-C1009	BioLegend
CD95	SA367H8	TotalSeq-C0917	BioLegend
CD96	3.3	TotalSeq-C0906	BioLegend

CD98	RL388	TotalSeq-C0989	BioLegend
CD103	2E7	TotalSeq-C0201	BioLegend
CD104	346-11A	TotalSeq-C1028	BioLegend
CD105	MJ7/18	TotalSeq-C0812	BioLegend
CD106	429 (MVCAM.A)	TotalSeq-C0226	BioLegend
CD115	AFS98	TotalSeq-C0105	BioLegend
CD117	2B8	TotalSeq-C0012	BioLegend
CD120b	TR75-89	TotalSeq-C0893	BioLegend
CD122	5H4	TotalSeq-C0227	BioLegend
CD124	I015F8	TotalSeq-C0916	BioLegend
CD127	A7R34	TotalSeq-C0198	BioLegend
CD134	OX-86	TotalSeq-C0195	BioLegend
CD135	A2F10	TotalSeq-C0098	BioLegend
CD137	17B5	TotalSeq-C0194	BioLegend
CD138	281-2	TotalSeq-C0810	BioLegend
CD140a	APA5	TotalSeq-C0573	BioLegend
CD146	P1H12	TotalSeq-C0134	BioLegend
CD150	TC15-12F12.2	TotalSeq-C0203	BioLegend
CD152	UC10-4B9	TotalSeq-C0388	BioLegend
CD155	TX56	TotalSeq-C1011	BioLegend
CD160	7H1	TotalSeq-C1006	BioLegend
CD163	S15049I	TotalSeq-C0417	BioLegend
CD169	3D6.112	TotalSeq-C0440	BioLegend
CD170	S17007L	TotalSeq-C0431	BioLegend
CD172a	P84	TotalSeq-C0422	BioLegend
CD178	MFL3	TotalSeq-C1012	BioLegend
CD182	SA044G4	TotalSeq-C0909	BioLegend
CD183	CXCR3-173	TotalSeq-C0228	BioLegend
CD185	L138D7	TotalSeq-C0846	BioLegend
CD186	SA051D1	TotalSeq-C0926	BioLegend
CD192	SA203G11	TotalSeq-C0426	BioLegend
CD193	J073E5	TotalSeq-C0808	BioLegend
CD194	2G12	TotalSeq-C0833	BioLegend
CD196	29-2L17	TotalSeq-C0225	BioLegend
CD197	4B12	TotalSeq-C0377	BioLegend
CD198	SA214G2	TotalSeq-C0907	BioLegend
CD200	OX-90	TotalSeq-C0079	BioLegend
CD200R	OX-110	TotalSeq-C0807	BioLegend
CD201	RCR-16	TotalSeq-C0439	BioLegend
CD204	1F8C33	TotalSeq-C0448	BioLegend
CD205	NLDC-145	TotalSeq-C1010	BioLegend
CD207	4C7	TotalSeq-C0437	BioLegend
CD210	1B1.3a	TotalSeq-C1032	BioLegend
CD223	C9B7W	TotalSeq-C0378	BioLegend
CD226	1,00E+06	TotalSeq-C0852	BioLegend
CD226	TX42.1	TotalSeq-C0949	BioLegend
CD252	RM134L	TotalSeq-C0924	BioLegend
CD270	HMHV-1B18	TotalSeq-C0885	BioLegend
CD272	6A6	TotalSeq-C0881	BioLegend
CD273	TY25	TotalSeq-C0914	BioLegend
CD274	MIH6	TotalSeq-C0190	BioLegend
CD278	C398.4A	TotalSeq-C0171	BioLegend
CD278	7E.17G9	TotalSeq-C0847	BioLegend

CD279	RMP1-30	TotalSeq-C0004	BioLegend
CD300LG	ZAQ5	TotalSeq-C0416	BioLegend
CD301b	URA-1	TotalSeq-C0566	BioLegend
CD304	3,00E+12	TotalSeq-C0552	BioLegend
CD309	Avas12	TotalSeq-C0553	BioLegend
CD314	CX5	TotalSeq-C0835	BioLegend
CD317	927	TotalSeq-C0811	BioLegend
CD326	G8.8	TotalSeq-C0449	BioLegend
CD335	29A1.4	TotalSeq-C0184	BioLegend
CD357	DTA-1	TotalSeq-C0193	BioLegend
CD366	RMT3-23	TotalSeq-C0003	BioLegend
CD370	7H11	TotalSeq-C0556	BioLegend
CD371	5D3/CLEC12A	TotalSeq-C0825	BioLegend
CX3CR1	SA011F11	TotalSeq-C0563	BioLegend
DLL1	HMD1-3	TotalSeq-C0884	BioLegend
DR3	4C12	TotalSeq-C0836	BioLegend
ESAM	1G8/ESAM	TotalSeq-C0596	BioLegend
F4/80	BM8	TotalSeq-C0114	BioLegend
FcεRIα	MAR-1	TotalSeq-C0115	BioLegend
FITC (FITC labeled anti-CD49a - Ha31/8)	FIT-22	TotalSeq-C0988	BioLegend
FR4	TH6	TotalSeq-C1058	BioLegend
GPR56	CG4	TotalSeq-C0912	BioLegend
H-2Kb	25-D1.16	TotalSeq-C0231	BioLegend
HA.11	16B12	TotalSeq-C1131	BioLegend
I-A/I-E	M5/114.15.2	TotalSeq-C0117	BioLegend
IgD	11-26c.2a	TotalSeq-C0571	BioLegend
IgG Isotype Ctrl	HTK888	TotalSeq-C0241	BioLegend
IgG1	RMG1-1	TotalSeq-C1167	BioLegend
IgG1, κ	MOPC-21	TotalSeq-C0090	BioLegend
IgG1, κ Isotype Ctrl	RTK2071	TotalSeq-C0236	BioLegend
IgG1, λ Isotype Ctrl	G0114F7	TotalSeq-C0237	BioLegend
IgG2a, κ	MOPC-173	TotalSeq-C0091	BioLegend
IgG2a, κ Isotype Ctrl	RTK2758	TotalSeq-C0238	BioLegend
IgG2b	RMG2b-1	TotalSeq-C1168	BioLegend
IgG2b κ	RTK4530	TotalSeq-C0095	BioLegend
IgG2b, κ Isotype Ctrl	MPC-11	TotalSeq-C0092	BioLegend
IgM	RMM-1	TotalSeq-C0450	BioLegend
IL-21R	4A9	TotalSeq-C0879	BioLegend
IL-33Rα	DIH9	TotalSeq-C0837	BioLegend
Integrin β7	FIB504	TotalSeq-C0214	BioLegend
JAML	4,00E+10	TotalSeq-C0877	BioLegend
KLRG1	2F1/KLRG1	TotalSeq-C0250	BioLegend
Ly108	330-AJ	TotalSeq-C0930	BioLegend
Ly49A	YE1/48.10.6	TotalSeq-C0842	BioLegend
Ly49D	4,00E+05	TotalSeq-C0841	BioLegend
Ly49G	AT8	TotalSeq-C0840	BioLegend
Ly49H	3D10	TotalSeq-C0839	BioLegend
Ly-51	6C3	TotalSeq-C1034	BioLegend
Ly-6A/E	D7	TotalSeq-C0130	BioLegend
Ly6C	HK1.4	TotalSeq-C0013	BioLegend
Ly6G	1A8	TotalSeq-C0015	BioLegend
Ly-6G/Ly-6C	RB6-8C5	TotalSeq-C0116	BioLegend

MAdCAM-1	MECA-367	TotalSeq-C0232	BioLegend
MERTK	2B10C42	TotalSeq-C0565	BioLegend
NK-1.1	PK136	TotalSeq-C0118	BioLegend
Notch 1	HMN1-12	TotalSeq-C0442	BioLegend
Notch 4	HMN4-14	TotalSeq-C0888	BioLegend
P2X7R	1F11	TotalSeq-C0824	BioLegend
Rat IgG2c, κ	RTK4174	TotalSeq-C0240	BioLegend
Siglec H	551	TotalSeq-C0119	BioLegend
TCR Va2	B20.1	TotalSeq-C0981	BioLegend
TCR Vα 8.3	B21.14	TotalSeq-C0982	BioLegend
TCR Vα 8.3	KT50	TotalSeq-C0983	BioLegend
TCR Vα11	RR8-1	TotalSeq-C0984	BioLegend
TCR Vβ5.1, 5.2	MR9-4	TotalSeq-C0354	BioLegend
TCR Vβ8.1,8.2	KJ16-133.18	TotalSeq-C0235	BioLegend
TCR Vγ1.1	2.11	TotalSeq-C0209	BioLegend
TCR Vγ2	UC3-10A6	TotalSeq-C0211	BioLegend
TCR Vγ3	536	TotalSeq-C0210	BioLegend
TCR β chain	H57-597	TotalSeq-C0120	BioLegend
TCR γ/δ	GL3	TotalSeq-C0121	BioLegend
TCR γ/δ	UC7-13D5	TotalSeq-C0986	BioLegend
TER-119	TER-119	TotalSeq-C0122	BioLegend
TIGIT	1G9	TotalSeq-C0848	BioLegend
Tim-4	RMT4-54	TotalSeq-C0567	BioLegend
VISTA	MIH63	TotalSeq-C0915	BioLegend
XCR1	ZET	TotalSeq-C0568	BioLegend

Supplementary references

1. **Huang Z**, **Brodeur KE**, **Chen L**, et al. Type I interferon signature and cycling lymphocytes in macrophage activation syndrome. J Clin Invest 2023;133(22):e165616.

2. **Andrews TS**, **Nakib D**, Perciani CT, et al. Single-cell, single-nucleus, and spatial transcriptomics characterization of the immunological landscape in the healthy and PSC human liver. J Hepatol 2024;80(5):730–43.

3. **Kim H**, **De Jesus AA**, **Brooks SR**, et al. Development of a Validated Interferon Score Using NanoString Technology. Journal of Interferon and Cytokine Research 2018;38(4):171–85

4. **Cui C**, **Xu C**, Yang W, et al. Ratio of the interferon-γ signature to the immunosuppression signature predicts anti-PD-1 therapy response in melanoma. NPJ Genom Med 2021;6(1).
